# Genome-Guided Discovery of an Antimicrobial Peptide Scaffold from an Undescribed Antarctic *Rhodococcus* Lineage: A Proof-of-Concept Study

**DOI:** 10.3390/ijms27156655

**Published:** 2026-07-25

**Authors:** Dayaimi González, Andrés Santos, Eulàlia Sans-Serramitjana, Fanny Guzmán, Carla Gallardo-Benavente, Dayani Hernández, Mercyleidi Díaz Reyes, Francisca Acevedo

**Affiliations:** 1 Programa de Doctorado en Ciencias Mención Biología Celular y Molecular Aplicada, Universidad de La Frontera, Temuco 4811230, Chile; d.gonzalez47@ufromail.cl; 2 Center of Excellence in Translational Medicine—Scientific and Technology Bioresource Nucleus (CEMT- BIOREN), Faculty of Medicine, Universidad de La Frontera, Avenida Alemania N° 0458, Temuco 4780000, Chile; andres.santos@ufrontera.cl (A.S.); eulalia.sans@ufrontera.cl (E.S.-S.); 3 Millennium Nucleus Bioproducts, Genomics and Environmental Microbiology (BioGEM), Avenida España 1680, Valparaíso 2390123, Chile; 4 Department of Basic Sciences, Faculty of Medicine, Universidad de La Frontera, Casilla 54-D, Temuco 4780000, Chile; 5 Center of Biomedical Informatics, Faculty of Medicine, Universidad de La Frontera, Avenida Alemania N° 0458, Temuco 4780000, Chile; 6 Department of Integral Adult Dentistry, Faculty of Dentistry, Universidad de La Frontera, Avenida Francisco Salazar 01145, Temuco 4780000, Chile; 7 Curauma Biotechnology Center, Pontificia Universidad Católica de Valparaíso, Valparaíso 2362807, Chile; fanny.guzman@pucv.cl; 8 Biotechnology Center for Microbial Studies (CEBEM), Universidad de La Frontera, Temuco 4811230, Chile; carlagallardo6@gmail.com; 9 Departamento de Cirugía General, Facultad de Medicina de Mayabeque, Universidad de Ciencias Médicas de La Habana, Mayabeque 32000, Cuba; dayanihernandezreyes@gmail.com; 10 Instituto de Biología Molecular y Celular de Rosario (IBR-CONICET), Universidad Nacional de Rosario (UNR), Rosario 2000, Argentina; diazreyes@ibr-conicet.gov.ar

**Keywords:** extremophilic microorganisms, phylogenomic analysis, secondary metabolite biosynthesis, nonribosomal peptide synthetases, antibiofilm activity

## Abstract

*Rhodococcus* species display remarkable metabolic versatility and adaptation to extreme environments, yet their antimicrobial potential remains largely unexplored, particularly in strains representing undescribed genomic lineages. This study investigated whether the genome of *Rhodococcus* sp. GB-02, a strain isolated from Antarctic soil, encodes antimicrobial peptides with demonstrable activity against clinically relevant pathogens. Hybrid genome assembly revealed a 6.58 Mb high-quality draft genome belonging to a previously undescribed genomic lineage within the genus *Rhodococcus*, based on average nucleotide identity and digital DNA–DNA hybridization analyses. Genome mining identified 20 biosynthetic gene clusters, predominantly non-ribosomal peptide synthetases, several of which showed low or no similarity to known pathways. Eight putative antimicrobial peptides (AMPs) were computationally predicted; among these, three lacked detectable homology to sequences in current databases, and experimental validation of one predicted peptide, designated Peptide 5764, demonstrated antibacterial activity at high micromolar concentrations against Gram-positive and Gram-negative pathogens and concentration-dependent inhibition of biofilm formation, particularly in *Pseudomonas aeruginosa*. However, Peptide 5764 exhibited considerable hemolytic activity at antibacterial concentrations, indicating that optimization for membrane selectivity will be required before therapeutic application. These findings establish *Rhodococcus* sp. GB-02 as an underexplored Antarctic lineage with a diverse biosynthetic repertoire and provide proof-of-concept that genome-guided AMP prediction can identify experimentally active peptide scaffolds from this lineage.

## 1. Introduction

Antarctica, with its extreme environmental conditions, has emerged as a unique and promising setting for uncovering remarkable bacterial diversity and for the discovery of novel natural products and antibacterial compounds [1]. Persistently low temperatures, intense UV radiation, limited nutrient availability, and recurrent freeze–thaw cycles impose strong selective pressures that shape microbial evolution, driving physiological adaptations and the emergence of specialized metabolic pathways [2]. These constraints often intensify competition for scarce resources, favoring the development of survival and defense strategies such as antagonistic activity and the production of antimicrobial secondary metabolites. To cope with multiple physiological stresses, including reduced enzyme activity, decreased membrane fluidity, oxidative stress, and inefficient nutrient transport, microorganisms evolve innovative metabolic capabilities and novel biosynthetic pathways responsible for the synthesis of chemically complex and structurally unique compounds [3]. Consequently, microorganisms from polar habitats are increasingly recognized as promising sources of chemically diverse and structurally novel antimicrobial compounds.

Among these microorganisms, the phylum *Actinomycetota* (commonly known as *Actinobacteria*) has received particular attention due to its capacity to synthesize a wide variety of bioactive compounds and secondary metabolites (SMs) with significant pharmacological and biotechnological applications [4,5]. Many of these compounds originate from biosynthetic gene clusters (BGCs) and represent a major source of antibiotics used in modern medicine [6]. Despite the contribution of SMs in the context of novel antibiotics, the continued rise of multidrug-resistant pathogens highlights the urgent need to identify new antimicrobial agents [7,8].

Antimicrobial peptides (AMPs) have emerged as attractive therapeutic candidates due to their broad-spectrum activity, rapid bactericidal mechanisms, and lower propensity for resistance development compared to conventional antibiotics [9,10,11,12]. *Actinomycetes* are increasingly recognized as a rich yet underexplored source of both ribosomally and non-ribosomally synthesized AMPs [13,14]. Thus, genome mining has become a powerful and rational strategy to explore the antimicrobial capacity of microorganisms by systematically identifying BGCs encoding antibiotics and AMPs, including silent or low-expression clusters that are not readily detected through activity-based screening approaches [15,16].

In this context, *Rhodococcus* is a genus within the family *Nocardiaceae* and the phylum *Actinomycetota* that has recently gained attention as a reservoir of untapped biosynthetic diversity with potential relevance for antimicrobial discovery [17,18]. First proposed by Zopf in 1891, the genus currently comprises 59 validly published species, according to the List of Prokaryotic Names with Standing in Nomenclature (LPSN) [19]. Members of this genus have been isolated from a wide variety of environments and hosts, including marine, freshwater, soil, animals, plants, and insects [20]. Although genomic analyses indicate that *Rhodococcus* species harbor numerous BGCs for SMs, only 24 SMs have been characterized to date. These include rhodopeptins [21], lariatins [22], aurachins [23], rhodostreptomycin [24], humimycins [25], and rhodochelin [26], which exhibit a range of bioactivities, including antibacterial, antifungal, antitrypanosomal, anticancer, and siderophore-related properties. Although *Rhodococcus* is well known for its capacity to degrade a broad spectrum of natural and xenobiotic compounds, its potential as a source of antimicrobial agents remains relatively unexplored [27].

Despite this biosynthetic richness, the vast majority of *Rhodococcus* species remain genomically uncharacterized, and their BGC repertoires have not been linked to functional antimicrobial activity. This gap is particularly acute for strains that represent undescribed lineages within the genus, where both taxonomic novelty and biosynthetic divergence from known species may coincide. Closing this gap requires moving beyond genome annotation toward experimental confirmation of predicted antimicrobial candidates. Therefore, this study aimed to explore the genome-encoded antimicrobial potential of *Rhodococcus* sp. GB-02, an Antarctic strain representing a previously undescribed lineage, through phylogenomic characterization, biosynthetic gene cluster analysis, genome-guided AMP prediction, and experimental validation of a prioritized peptide candidate. This study links genomic predictions with functional evidence, providing the first experimental support for the genome-encoded antimicrobial potential of this Antarctic *Rhodococcus* lineage.

## 2. Results

### 2.1. Genomic Features of Rhodococcus sp. GB-02

**Figure 1:**
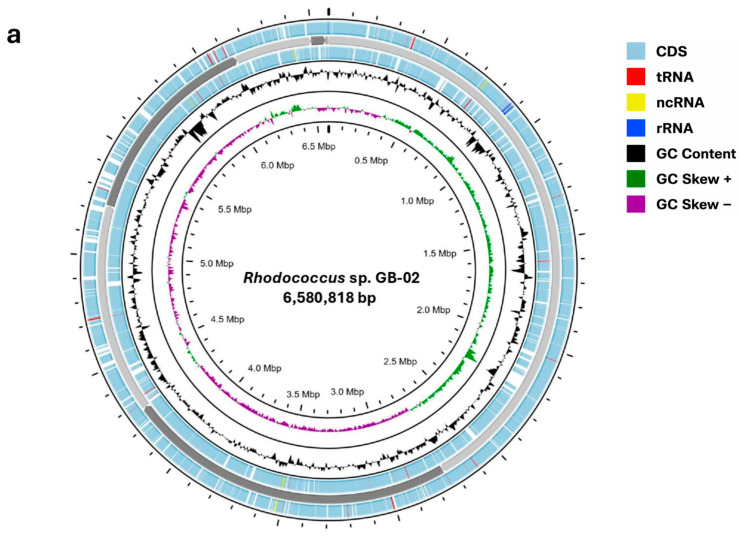

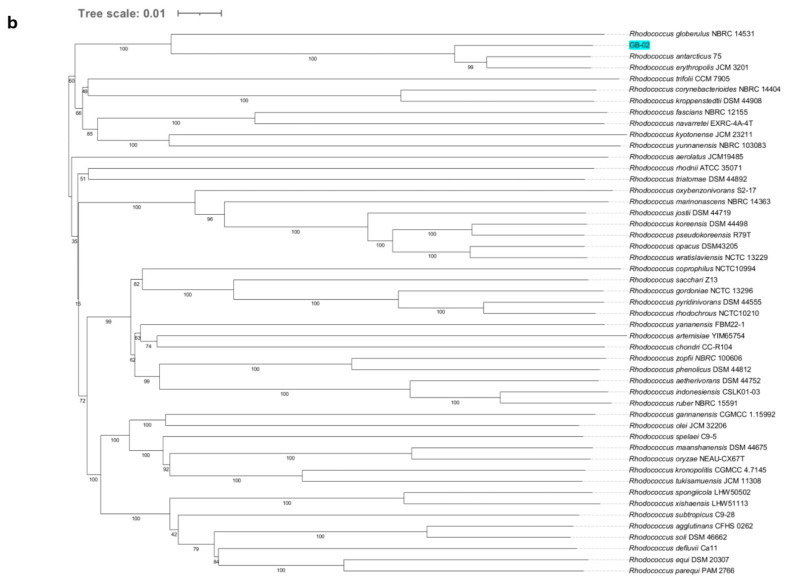
(**a**)**.** Schematic circular representation of the draft genome of strain GB-02 (15 contigs) generated using Proksee. Coding sequences (CDS, light blue), tRNAs (red), ncRNA (yellow), rRNAs (dark blue), GC content (black), and GC skew curves (+/−, green/purple). (**b**) Phylogenomic tree of genomes of strain GB-02 (highlighted in blue) and *Rhodococcus* type strains, inferred using the Type Strain Genome Server (TYGS). The numbers above the branches represent genome BLAST distance phylogeny (GBDP) pseudo-bootstrap values greater than 60% based on 100 replicates. The scale bar corresponds to 0.01 substitutions per nucleotide position.

The draft genome of strain GB-02, obtained through hybrid assembly, consisted of 15 contigs with an N50 of 1,524,266 bp, a total length of 6,580,818 bp, an average sequencing depth of 98.81×, and 62.3% GC content. A total of 6092 protein-encoding genes and 67 RNA genes (including 52 transfer RNA (tRNA), 12 ribosomal RNA (rRNA), and 3 non-coding RNA (ncRNA)) were predicted in the genome. A schematic visualization of the genome sequence is shown in Figure 1a.

Figure 1b shows that strain GB-02 is most closely related to two type strains, *Rhodococcus erythropolis* JCM 3201 and *Rhodococcus antarcticus* 75, confirming its placement within the genus *Rhodococcus* (family *Nocardiaceae,* phylum *Actinomycetota*). However, digital DNA–DNA hybridization (dDDH) and Average Nucleotide Identity (ANI) values between GB-02 and these type strains remained below the accepted species-level thresholds (70% for dDDH and 95–96% for ANI) (Table 1), indicating that GB-02 does not belong to any currently validly described species [28,29]. A genome-wide ANI comparison against publicly available *Rhodococcus* assemblies further revealed that only two genomes exceeded the 95–96% species delineation threshold when compared with GB-02, namely *Rhodococcus erythropolis* IEGM 1415 (GCF_027270535.1) and *Rhodococcus* sp. IEGM 1318 (GCF_033142465.1), showing ANI values of 98.1% and 98.7%, respectively (Supplementary Table S1). These genomes do not correspond to type strains of validly published species. Collectively, these results indicate that, although GB-02 belongs to the same genomic species as these isolates, it cannot be assigned to any currently validly described species and therefore represents a distinct genomic lineage within the genus that has not yet been formally described.

**Figure 2:**
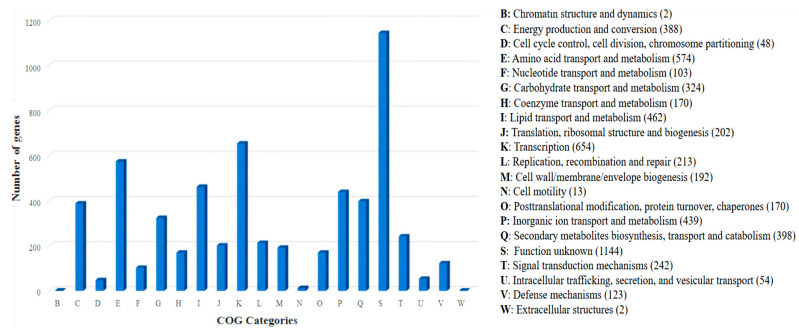
COG functional category distribution of *Rhodococcus* sp. GB-02.

Functional analysis based on Clusters of Orthologous Groups (COG) was performed using eggNOG mapper v2. For strain GB-02, 5917 genes out of 6261 (94.51%) were classified into 21 different COG categories (Figure 2). The function unknown (S: 1144) category had the highest number, implicating the yet-to-explore potential of this isolate. The highest number of annotated genes was involved in metabolism (2858 genes, 48.30%), followed by information storage and processing (1071 genes, 18.10%), and cellular processes and signaling (844 genes, 14.26%). Among them, 654 coding sequences (CDSs) were associated with transcription, highlighting the role of cells in regulating gene expression and protein synthesis. In addition, 574 CDSs were related to amino acid transport and metabolism, suggesting a strong metabolic capacity for synthesizing, degrading, and importing amino acids.

**Figure 3:**
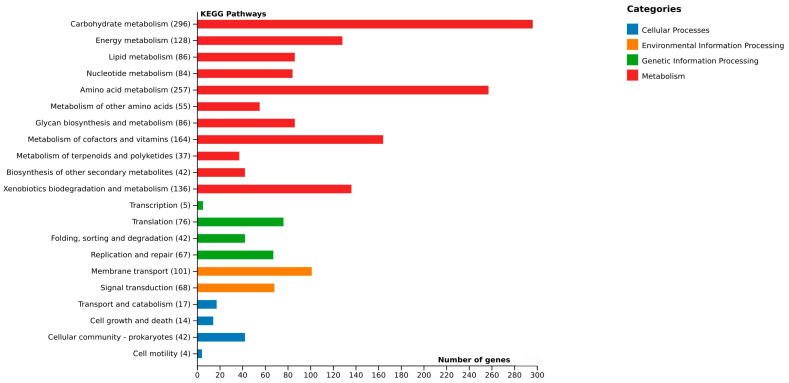
KEGG pathway and metabolic module reconstruction of *Rhodococcus* sp. GB-02.

KEGG pathway analysis (Figure 3) enabled the reconstruction of metabolic pathways and functional modules encoded in the GB-02 genome, providing insight into its metabolic architecture beyond global functional classification. A total of 1999 genes (31.93% of all CDSs) were assigned to 41 KEGG pathways, revealing a highly interconnected metabolic network. Rather than reflecting overall functional abundance, KEGG analysis highlighted the presence of complete and alternative metabolic routes, including central carbon metabolism, energy production, and specialized biosynthetic processes.

Specifically, GB-02 harbors pathways associated with carbohydrate, amino acid, and nucleotide metabolism, membrane transport, and specialized metabolic functions, including terpenoid and polyketide metabolism and the biosynthesis of secondary metabolites, following KEGG pathway classification. The enrichment of membrane transport pathways (101 genes, 5.05%) within the environmental information processing category suggests an enhanced capacity for substrate uptake and stress adaptation.

Beyond pathway abundance, functional module analysis (Supplementary Table S2) revealed a metabolically versatile central system integrating classical glycolysis, the pentose phosphate pathway (modules M00001, M00002, M00003, M00004, M00006 and M00007), and the Entner–Doudoroff pathway (M00008), the complete tricarboxylic acid cycle (M00009–M00011), and the glyoxylate shunt (M00012), indicating metabolic flexibility for carbon assimilation and energy generation. The genome also encoded complete pathways for the biosynthesis of essential amino acids, nucleotides, vitamins, and cofactors, including glutathione (M00118), pantothenate and coenzyme A (M00119–M00120), biotin (M00123), riboflavin (M00125), tetrahydrofolate (M00126), thiamine (M00127), pyridoxal phosphate (M00916), and menaquinone (M00116), supporting extensive biosynthetic autonomy.

Modules involved in respiratory energy metabolism were also well represented, including NADH dehydrogenase (M00144), succinate dehydrogenase (M00149), cytochrome oxidases (M00151, M00153 and M00155), and F-type ATP synthase (M00157), indicating a complete and efficient aerobic electron transport chain. Additional adaptive functions included multidrug efflux systems (M00649, M00700, M00702, M00705, M00714 and M00718), resistance to cationic antimicrobial peptides through the PgtE system (M00744), biosynthesis of the osmoprotectant betaine (M00555), trehalose (M00565), and ectoine degradation (M00919), together with siderophore biosynthesis (M00875), all of which are consistent with enhanced tolerance to environmental stress and nutrient limitation.

Furthermore, GB-02 encoded several specialized metabolic modules, including β-carotene biosynthesis (M00097), nicotine degradation (M00810), the biosynthesis of the C-1027 β-amino acid moiety (M00827), and the futalosine pathways for menaquinone biosynthesis (M00930 and M00931). Collectively, these modules suggest an expanded capacity for respiratory flexibility, oxidative stress protection, membrane adaptation, iron acquisition, and specialized metabolism, supporting the ecological fitness of GB-02 and providing a functional genomic framework consistent with its antimicrobial phenotype.

Comparative analysis against *Rhodococcus erythropolis* IEGM 1415 (GCF_027270535.1) and *Rhodococcus* sp. IEGM 1318 (GCF_033142465.1), identified 26 KEGG modules that were exclusively present in GB-02 (Supplementary Table S3), suggesting an expanded repertoire of metabolic and adaptive capabilities. These modules include pathways related to membrane remodeling and lipid metabolism (M00059, M00067, M00068 and M00090), antioxidant protection through β-carotene biosynthesis (M00097), glutathione biosynthesis (M00118), and one-carbon metabolism (M00140), all of which may contribute to cellular homeostasis under environmental stress. GB-02 also uniquely encoded the cytochrome bc1 respiratory complex (M00152) together with the futalosine and modified futalosine pathways for menaquinone biosynthesis (M00930 and M00931), indicating additional flexibility in respiratory electron transport. Several modules associated with environmental adaptation were also exclusive to GB-02, including multidrug efflux systems (M00649 and M00718), resistance to cationic antimicrobial peptides (M00744), siderophore biosynthesis through the staphyloferrin B pathway (M00875), nicotine degradation (M00810), pyocyanine-related secondary metabolite biosynthesis (M00835), and the biosynthesis of the C-1027 β-amino acid moiety (M00827), highlighting an enhanced capacity for competition and survival in nutrient-limited environments. Overall, the exclusive metabolic profile of GB-02 supports a genome enriched in respiratory versatility, stress tolerance, membrane adaptation, iron acquisition, and specialized metabolism, features that are consistent with ecological adaptation and may contribute to its antimicrobial potential. Together, these differences delineate a biosynthetic and adaptive repertoire in GB-02 that is distinct from its closest phylogenomic relatives, and which provides a genomic basis for the antimicrobial potential explored in this study.

### 2.2. Secondary Metabolite Analysis

#### 2.2.1. BGCs

Genome mining was performed to characterize the antimicrobial biosynthetic potential encoded within the genome of GB-02 through the identification of BGCs. Twenty gene clusters for SMs were identified in the genome, with a higher prevalence of non-ribosomal peptide synthetases, polyketides, and terpenes (Table 2). Most of the BGCs were non-ribosomal peptide synthetases (NRPS; 9 of 20), four of which were hybrid types (2 NRP-metallophore, NRPS, 1 NRPS, terpene, and 1 Arylpolyene/terpene), and one was classified as a non-ribosomal peptide synthetase-like fragment (NRPS-like). Each of the following clusters was also identified: post-translationally modified peptide product (RiPP-like), linear azol(in)e-containing peptides (LAP), type I polyketide synthase (T1PKS), non-alpha poly-amino acids like e-Polylysin (NAPAA), redox-cofactor, butyrolactone, lanthipeptide class III, heterocyst glycolipid synthase-like PKS (hglE-KS), terpene and ectoine.

Comparison of the BGC profile of GB-02 against the 68 *Rhodococcus* genomes analyzed in this study revealed that its overall BGC richness (20 clusters) is consistent with the genus-wide mean for both the Antarctic (19.3 ± 3.9) and Global (19.9 ± 6.3) groups (Supplementary Table S4). However, its qualitative composition is notably distinct. Whereas NRPS clusters dominate the profiles of the highest-richness strains in the dataset—*R. marinonascens* NBRC 14363, *R. wratislaviensis* NCTC13229, and *R. koreensis* DSM 44498 each carrying 14–17 NRPS clusters—GB-02 presents a more diversified architecture with a comparatively high proportion of hybrid BGCs (four clusters), placing it among the top 15% of the genus for hybrid cluster abundance. This structural diversity in hybrid BGCs is particularly relevant, as these architectures often produce structurally complex metabolites through the combined action of multiple biosynthetic machineries.

The heatmap-based clustering (Supplementary Material: Figure S1) further revealed that the three rarest BGC subclasses across the dataset—azole-containing RiPP (present in 10% of genomes), hglE-KS (10%), and lanthipeptide class III (16%)—co-occur exclusively in GB-02 and two Antarctic strains, *R. erythropolis* IEGM 1415 and *Rhodococcus* sp. IEGM 1318. Furthermore, lanthipeptide class III and hglE-KS show a marked enrichment in the Antarctic group compared to Global strains (45% vs. 2% and 25% vs. 2%, respectively), suggesting these rare cluster types may be more prevalent in bacteria adapted to cold environments. GB-02 is the only non-IEGM strain carrying this three-way combination, making its RiPP-related biosynthetic repertoire particularly noteworthy.

Four BGCs exhibited 100% similarity to known clusters with siderophore and antimicrobial properties (ε-poly-L-lysine, corynecin, heterobactin B, and aurachin RE). Additionally, three BGCs (RiPP-like, ectoine, and the hybrid NRP-metallophore/NRPS cluster) exhibited similarity values exceeding 50%. Although no universally accepted cutoff has been established to define similarity between BGCs, similarity values above 50% have been used as an operational criterion in comparative studies to identify related BGCs [8,18,30]. The remaining BGCs showed low or no similarity to reference clusters in the MIBiG database, highlighting their potential as candidates for previously unexplored biosynthetic diversity that remains to be experimentally validated. It is important to note that only one BGC (region 2.2) spans contig boundaries (Supplementary Table S5), which indicates a high level of completeness and a reduced probability of artifactual detection of lower similarity scores.

Multiple regulatory genes associated with the 20 BGCs identified in the GB-02 genome were detected using antiSMASH, including representatives of the TetR, LysR, GntR, AraC, LuxR, ArsR, and MerR families, as well as sigma factors (Supplementary Material: Table S6). Most BGCs harbored at least one regulatory gene, with particularly complex regulatory architectures observed in clusters involved in the biosynthesis of NRPS-derived compounds and siderophores. These included two-component systems composed of sensor histidine kinases and response regulators, in addition to sigma factors (σ24 and σ54), suggesting regulatory mechanisms associated with environmental signal perception and secondary metabolite biosynthesis. Notably, TetR family regulators were the most abundant, being detected in 10 out of the 20 BGCs identified in strain GB-02, highlighting their prominent role in the transcriptional regulation of secondary metabolism.

RiPP-associated regions were further analyzed using BAGEL4, which confirmed the antiSMASH predictions and refined the annotation of the RiPP-like cluster (Supplementary Material: Figure S2).

#### 2.2.2. Predicted Antimicrobial Peptide Candidates

**Figure 4:**
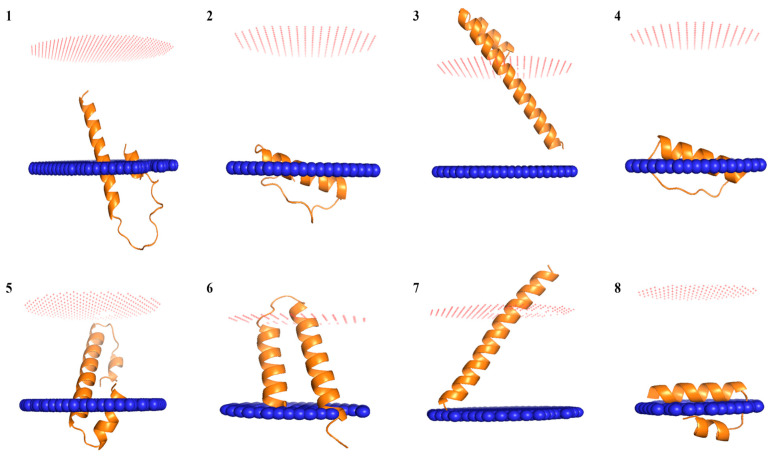
Structural modeling and computationally predicted orientations relative to a membrane-mimetic environment of antimicrobial peptides encoded in the GB-02 genome. Panels 1–8 correspond to AMPs 1–8 listed in Table 3, respectively.Antimicrobial peptides (orange), extracellular side of the membrane (red), and cytoplasmic side (blue).

The BGC analysis (Section 2.2.1) and crude extract bioassays (Supplementary Material: Table S7 and Figure S3) collectively indicated that a substantial fraction of the biosynthetic capacity of GB-02 is either structurally complex, cryptically regulated, or expressed below detectable levels (50 mg/mL) under standard laboratory conditions. To complement this analysis and access antimicrobial determinants independently of expression levels, a genome-wide scan for small peptides was performed. The peptide candidates described in this section are distinct from the RiPP clusters identified by antiSMASH and BAGEL (Section 2.2.2). Rather than arising from dedicated biosynthetic gene clusters with associated modification enzymes, these candidates correspond to small open reading frames (≤100 aa) distributed across the genome that were identified through machine learning and physicochemical property-based AMP prediction tools. They do not carry canonical RiPP precursor features such as leader peptides or RiPP Recognition Elements (RREs), and should therefore be interpreted as a complementary and independent layer of genomic antimicrobial potential. Genome-wide prediction of small peptides (≤100 aa) using AI4AMP, AMPA, and CAMPR4 identified eight candidate AMPs in the GB-02 genome (Table 3). Five candidates showed low-level similarity (<40%) to known peptides in the APD3 database; peptides 3, 4, and 7 showed no detectable homology to any characterized sequence, indicating they are absent from current AMP repositories. Structural modeling predicted predominantly α-helical conformations for all eight candidates, with variable membrane orientations (Figure 4). Among the three APD3-negative candidates, peptide 4 (hereafter Peptide 5764) was prioritized for experimental validation based on its strongly hydrophobic physicochemical profile (GRAVY index 1.178, hydrophobicity 63%) and predicted amphipathic α-helical organization.

Exploratory molecular docking analyses were also performed for peptides 3, 4, and 7 against proteins associated with quorum sensing, virulence regulation, adhesion, and biofilm formation in representative ESKAPEE pathogens (Supplementary Table S8 and Supplementary Figures S4–S7).

### 2.3. Antibacterial Activity of Peptide 5764

To experimentally validate the antimicrobial activity of peptide 4, hereafter referred to as Peptide 5764, the genome-predicted peptide was chemically synthesized and evaluated independently of the crude extract bioassays. The antibacterial activity of this peptide was evaluated against clinically relevant Gram-positive and Gram-negative bacterial strains by determining minimum inhibitory concentration (MIC) and minimum bactericidal concentration (MBC) values (Table 4). Peptide 5764 was selected for proof-of-concept experimental validation based on three explicit criteria. First, it showed no detectable homology to any characterized sequence in the APD3 database, establishing it as a genuinely novel candidate absent from current antimicrobial peptide repositories. Second, among the three APD3-negative candidates (peptides 3, 4, and 7), peptide 5764 presented the most favorable physicochemical profile for membrane-active antimicrobial activity: the shortest sequence (27 aa), the highest hydrophobicity (GRAVY index 1.178; hydrophobic residue content 63%), and a predicted amphipathic α-helical conformation, all of which are characteristics commonly associated with enhanced interactions with bacterial membranes and membrane-disrupting mechanisms of action. Third, its shorter length conferred greater tractability for chemical synthesis by solid-phase methods compared to peptides 3 and 7 (49 and 36 aa, respectively), which were instead prioritized for exploratory molecular docking analyses against quorum-sensing and biofilm-associated targets. Together, these criteria provide a structured and transparent basis for the selection of peptide 5764 and rule out arbitrary prioritization among the novel candidates identified.

Peptide 5764 is encoded within contig 1, at genomic coordinates from 1,411,644 to 1,412,021 bp, in a region not overlapping with any of the 20 BGCs predicted by antiSMASH, confirming that it represents a biosynthetic determinant independent of the characterized secondary metabolite clusters.

The peptide exhibited inhibitory activity against *S. aureus* ATCC 25923, *S. aureus* clinical isolate (STA003), *E. faecium* (EN201), *E. coli* ATCC 25922, and *P. aeruginosa* ATCC 27853, with MIC and MBC values of 284 μg/mL (≈98 μM)and 568 μg/mL (≈196 μM), respectively. In contrast, no detectable inhibitory activity was observed against the clinical isolate of *P. aeruginosa* or against *Klebsiella pneumoniae* strains at the highest concentration tested (MIC > 568 μg/mL). The MBC values were consistently two-fold higher than MIC values, indicating a concentration-dependent bactericidal effect of Peptide 5764. It should be noted, however, that the MIC values obtained for Peptide 5764 (284 μg/mL, ≈98 μM) are substantially higher than those of clinical antibiotics, and the observed activity should therefore be interpreted as an early-stage in vitro finding rather than evidence of therapeutic efficacy.

The susceptibility profiles obtained with conventional antibiotics demonstrated substantial variability among strains. Tobramycin exhibited strong antibacterial activity against *P. aeruginosa* strains (MIC < 0.5 μg/mL), whereas resistance was observed in *S. aureus* strains and *K. pneumoniae* isolates. Ciprofloxacin displayed high antibacterial potency against most strains but showed limited activity against *E. faecium* (EN201) and *K. pneumoniae* strains. Interestingly, Peptide 5764 retained activity against bacterial strains resistant to at least one conventional antibiotic, including *E. faecium* (EN201) and both *S. aureus* strains resistant to tobramycin.

### 2.4. Antibiofilm Effect of Peptide 5764

**Figure 5:**
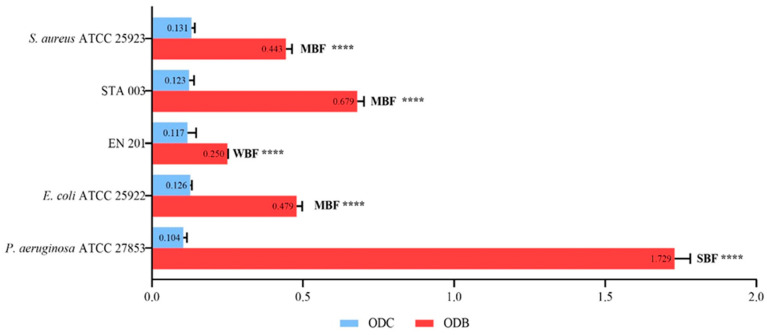
Evaluation of bacterial biofilm formation based on bacterial biofilm optical density (ODB) values relative to the optical density of the negative control (ODC), according to biofilm production criteria. Data are presented as mean ± SD (*n* = 3). Statistical analysis was performed using two-way ANOVA followed by Šídák’s multiple comparisons test (**** *p* < 0.0001). Based on ODB and ODC values, strains were classified as weak (WBF), moderate (MBF), or strong (SBF) biofilm formers.

The evaluated bacterial strains exhibited heterogeneous biofilm-forming capacities. Based on the ratio between the optical density of the bacterial biofilm (ODB) and the optical density cut-off (ODC), strains were classified as weak (ODC < ODB ≤ 2 × ODC), moderate (2 × ODC < ODB ≤ 4 × ODC), or strong (ODB > 4 × ODC) biofilm producers (Figure 5). Both *S. aureus* strains and *E. coli* ATCC 25922 were classified as moderate biofilm formers, whereas *P. aeruginosa* ATCC 27853 was categorized as a strong biofilm producer. In contrast, *E. faecium* EN201 exhibited weak biofilm formation.

**Figure 6:**
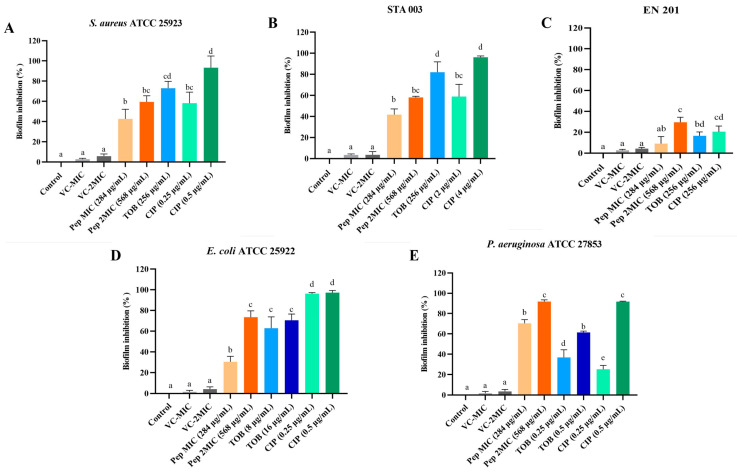
Biofilm inhibition in (**A**) *Staphylococcus aureus* ATCC 25923, (**B**) *S. aureus* STA003, (**C**) *Enterococcus faecium* EN201, (**D**) *Escherichia coli* ATCC 25922, and (**E**) *Pseudomonas aeruginosa* ATCC 27853 after treatment with peptide concentrations corresponding to MIC (284 μg/mL) and 2×MIC (568 μg/mL), as well as tobramycin (TOB) and ciprofloxacin (CIP) at the indicated concentrations.Data are presented as mean ± SD (*n* = 3). Statistical analysis was performed using one-way ANOVA followed by Tukey’s multiple comparisons test. Bars labeled with different lowercase letters indicate statistically significant differences among treatments within each bacterial strain (*p* < 0.05).

The antibiofilm activity of Peptide 5764 was evaluated at MIC (284 μg/mL) and 2 × MIC (568 μg/mL) concentrations (Figure 6). No statistically significant differences were observed between the untreated control, VC-MIC, and VC-2MIC groups for any evaluated strain, indicating that the vehicle solution did not interfere with biofilm formation and confirming that the observed antibiofilm effects were attributable exclusively to peptide treatment.

Peptide 5764 inhibited biofilm formation in all tested strains in a concentration-dependent manner, although susceptibility varied among species. *P. aeruginosa* ATCC 27853 exhibited the highest susceptibility, with inhibition percentages of 70.39% and 91.80% at MIC and 2 × MIC, respectively. Notably, despite being classified as the strongest biofilm producer, inhibition at 2 × MIC was comparable to ciprofloxacin treatment (91.75% inhibition at 0.5 μg/mL) and markedly higher than that obtained with tobramycin (36.83% and 61.40% inhibition at 0.25 and 0.5 μg/mL, respectively). However, this comparison should be interpreted with caution, as these inhibition percentages were achieved at substantially different concentrations (568 μg/mL for Peptide 5764 vs. 0.5 μg/mL for ciprofloxacin), reflecting a considerable difference in potency.

Both *S. aureus* strains exhibited moderate to high inhibition levels, reaching approximately 58–60% inhibition at 2 × MIC, with activities comparable to ciprofloxacin but slightly lower than those obtained with tobramycin. Similarly, *E. coli* ATCC 25922 showed a marked increase in inhibition from 30.5% at MIC to 69.2% at 2 × MIC, displaying activity comparable to tobramycin at the highest concentration tested. In contrast, *E. faecium* EN201 exhibited the lowest susceptibility, with inhibition percentages of 9.2% and 29.7% at MIC and 2 × MIC, respectively, although a concentration-dependent effect was still observed.

### 2.5. Hemolytic Activity and Peptide Toxicity

**Figure 7:**
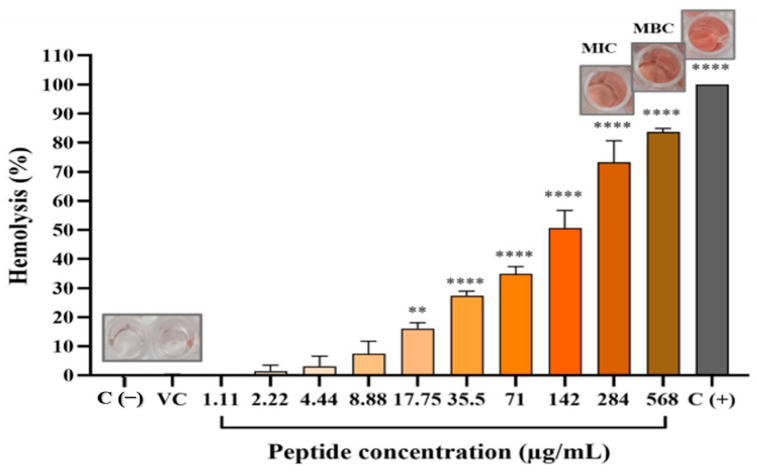
Hemolysis of erythrocytes induced by different concentrations of peptide 5764. PBS, ultrapure water supplemented with 5% acetic acid (5 mM final concentration), and 0.1% Triton X-100 were used as negative [C (−)], vehicle (VC), and positive [C (+)] controls, respectively. Representative images of erythrocyte suspensions at the concentrations corresponding to MIC and MBC values are shown. Data are presented as mean ± SD (*n* = 3). Statistical analysis was performed using one-way ANOVA followed by Dunnett’s multiple comparisons test using the negative control as reference. Asterisks indicate statistically significant differences compared with the negative control (** *p* < 0.01; **** *p* < 0.0001).

The hemolytic activity of Peptide 5764 was evaluated using erythrocytes exposed to increasing peptide concentrations, including concentrations corresponding to MIC and MBC values (Figure 7). No statistically significant differences were observed between the negative control [C (−)] and the vehicle control (VC), indicating that the solvent used for peptide dissolution did not induce erythrocyte lysis. At lower peptide concentrations (1.11–8.88 μg/mL), hemolysis levels remained low and did not significantly differ from the negative control. However, a concentration-dependent increase in hemolytic activity was observed at concentrations ≥ 17.75 μg/mL, with statistically significant differences relative to the negative control. Critically, concentrations corresponding to MIC (284 μg/mL) and MBC (568 μg/mL) values induced elevated hemolysis percentages of approximately 73% and 84%, respectively. Therefore, the hemolytic activity is therapeutically prohibitive at the concentrations required for antibacterial activity. These values approached the hemolytic effect observed for the positive control [C (+)], indicating substantial erythrocyte membrane disruption at antibacterial concentrations.

## 3. Discussion

*Rhodococcus* spp. are Gram-positive, aerobic, non-sporulating bacteria with high G+C content (61–71%) and large genomes (5–10 Mb), commonly found in soil, water, and marine sediments [31,32,33]. Strain GB-02, isolated from Antarctic soil, has a 6,580,818 bp genome with a G+C content of 62.3%, consistent with the genomic characteristics reported for members of the genus *Rhodococcus.* Importantly, the phylogenomic results confirm that strain GB-02 represents a previously undescribed genomic lineage within the genus. To investigate the antimicrobial potential encoded by this unique lineage, this study integrated genome mining, biosynthetic gene cluster characterization, antimicrobial peptide prediction, and experimental validation of a prioritized peptide candidate.

### 3.1. Genetic and Metabolic Versatility of the Strain GB-02

The genomic features of strain GB-02 indicate a high degree of metabolic versatility, a hallmark of *Rhodococcus* species adapted to extreme and competitive environments. The substantial fraction of genes assigned to the “function unknown” COG category (19.33%) is consistent with previous observations in Antarctic soil bacteria and suggests the presence of unexplored metabolic functions potentially linked to secondary metabolite biosynthesis [34].

The functional annotation of the GB-02 genome is consistent with a biosynthetically specialized strain rather than a metabolic generalist. Of the 6092 predicted protein-encoding genes, 654 CDSs (10.74%) were assigned to transcription-related functions (COG category K), and 101 genes (5.05% of KEGG-annotated CDSs) were associated with membrane transport pathways, suggesting both the regulatory complexity and the efflux infrastructure commonly associated with secondary metabolite biosynthesis and self-resistance in antibiotic-producing Actinobacteria [35,36,37]. Notably, 88 of these transport genes encode ABC-type transporters, which in Actinobacteria have been specifically implicated in the secretion of non-ribosomal peptides, polyketides, and other bioactive secondary metabolites [35,36]. The genome of GB-02 combines an extensive repertoire of biosynthetic gene clusters with metabolic features that are consistent with adaptation to nutrient-limited environments. AntiSMASH analysis identified multiple PKS and NRPS biosynthetic gene clusters together with tailoring enzymes involved in glycosylation, methylation, and other post-synthetic modifications, indicating the genetic potential to synthesize structurally diverse specialized metabolites. These findings are further supported by KEGG module reconstruction, which revealed exclusive metabolic functions relative to the closest genomes, including modules involved in respiratory electron transport (cytochrome bc1 complex and futalosine-dependent menaquinone biosynthesis), oxidative stress protection (β-carotene biosynthesis), one-carbon metabolism, siderophore production, antimicrobial peptide resistance, multidrug efflux, and specialized aromatic compound metabolism. Collectively, these features suggest that GB-02 possesses an expanded capacity for respiratory flexibility, environmental adaptation, and specialized metabolism, traits that are likely advantageous in Antarctic ecosystems and may contribute to the production of bioactive secondary metabolites.

Nevertheless, the scope of the current analysis is limited to two reference strains, both of which fall well below the species delineation threshold relative to GB-02. Broader comparative analyses encompassing a representative set of the 59 currently described *Rhodococcus* species will be necessary to establish whether these exclusive modules reflect lineage-level biosynthetic divergence or a more widely distributed but unannotated capacity within the genus. Within the constraints of the present comparison, GB-02 harbors a metabolic module configuration that is distinguishable from both its phylogenetically closest non-Antarctic and Antarctic relatives, a finding that is coherent with, but not sufficient to confirm, biosynthetic specialization at the lineage level.

### 3.2. NRPS as the Most Promising BGC in the Strain GB-02

NRPS are key enzymes involved in the biosynthesis of structurally diverse natural products, many of which exhibit potent antimicrobial activity [38]. Their modular architecture enables the incorporation of diverse amino acids, generating metabolites with enhanced stability and unique modes of action [39]. In the GB-02 genome, NRPS represented the most abundant BGC class, consistent with the biosynthetic profiles reported for other *Rhodococcus* species [40,41,42]. The biomedical relevance of NRPS is underscored by their role in the biosynthesis of clinically important antibiotics, such as actinomycin, penicillin, and vancomycin [43], as well as numerous antimicrobial compounds isolated and characterized from *Actinobacteria* [44].

Additionally, seven BGCs in GB-02 showed >50% similarity to known clusters. Among them, NRPS and PKS clusters such as NAPAA (region 1.4), NRPS (region 1.7), NRP-metallophore, NRPS (region 3.3), and arylpolyene (region 4.6) were 100% similar to BGCs producing ε-poly-L-lysine, corynecin, heterobactin, and aurachin RE, respectively. ε-Poly-L-lysine and corynecins are recognized antimicrobials [45,46], while heterobactins, as high-affinity siderophores, limit iron availability to competitors, indirectly inhibiting growth [47]. In addition, aurachin RE, a prenylated quinoline antibiotic originally isolated from *Rhodococcus*, exhibits strong activity against Gram-positive bacteria [48]. These results highlight GB-02’s potential to produce known bioactive metabolites.

### 3.3. Regulatory Capacity of SMs Biosynthesis in the Strain GB-02

BGCs and their metabolites help bacteria adapt to habitat-specific pressures. In soil *Actinobacteria*, TetR/AcrR family regulatory genes within BGCs respond to stresses such as metals and antibiotics [6,49]. In GB-02, the identification of regulatory genes within several BGCs suggests that the expression of these biosynthetic pathways may be responsive to environmental and physiological cues.

Regulatory genes within GB-02 BGCs—including TetR/ArsR repressors, LuxR-family activators, LysR-type dual regulators, and ECF sigma factors—indicate a sophisticated transcriptional control architecture capable of silencing or inducing secondary metabolite production in response to environmental cues. This regulatory complexity is consistent with the low bioactivity observed in crude extract assays under standard laboratory conditions and suggests that much of GB-02’s biosynthetic capacity is cryptic rather than absent.

### 3.4. Cryptic Antimicrobial Potential in the Strain GB-02

Although GB-02 harbors numerous BGCs for antimicrobial biosynthesis, crude extracts showed limited activity under laboratory conditions, likely due to low expression or metabolite levels. This aligns with the presence of transcriptional regulators, particularly TetR-type, which can keep BGCs silent or lowly expressed unless specific environmental cues are present [50].

Therefore, the low observed bioactivity does not contradict the antimicrobial potential inferred from genome mining but rather suggests that much of this potential may remain cryptic. Accordingly, strategies such as optimization of culture parameters, co-cultivation with competing microorganisms, or genetic manipulation of regulatory elements to relieve transcriptional repression may be required to activate silent BGCs and fully access the antimicrobial repertoire encoded by the GB-02 genome [51,52,53].

In this context, genome-guided AMP prediction represented a complementary strategy to explore the antimicrobial potential of GB-02 independently of metabolite expression levels. This approach is particularly well-suited to the biological profile of GB-02: a strain with a complex regulatory architecture that likely suppresses BGC expression under laboratory conditions, a draft genome of high completeness that enables reliable ORF detection, and membership in an undescribed lineage where the absence of prior experimental data makes expression-independent screening especially valuable. By scanning the full proteome for small peptides with predicted physicochemical profiles compatible with membrane-active antimicrobial activity, this strategy identified candidates that would not have been detected through BGC-based genome mining alone, as they lack the hallmarks of canonical biosynthetic clusters. The experimental confirmation of activity for Peptide 5764—a peptide identified through this complementary layer of analysis—directly validates the rationale for integrating both approaches in the study of GB-02’s antimicrobial potential.

### 3.5. Strain GB-02 as a Promising Source of Novel AMPs

Experimental validation of Peptide 5764 demonstrated antibacterial activity at high micromolar concentrations (MIC ≈ 98 μM) against five of the eight strains tested, and concentration-dependent inhibition of biofilm formation across all evaluated strains. These results should be interpreted within the limitations of the current experimental scope: the MIC values obtained are substantially higher than those of clinical antibiotics, and the peptide exhibited considerable hemolytic activity at antibacterial concentrations, precluding direct therapeutic application in its current form. The pronounced inhibition observed against *P. aeruginosa* is especially relevant because *P. aeruginosa* exhibited the highest biofilm-forming capacity among the evaluated strains and is strongly associated with persistent biofilm-mediated infections and elevated antimicrobial tolerance [54,55]. Notably, the antibiofilm activity of Peptide 5764 against *P. aeruginosa* was comparable to that observed for ciprofloxacin and exceeded that obtained with tobramycin, suggesting that the peptide may interfere with processes associated with biofilm establishment and maturation rather than acting exclusively through inhibition of planktonic bacterial growth [56,57].

The concentration-dependent hemolytic behavior observed for Peptide 5764 is consistent with the amphipathic and membrane-active characteristics predicted from its physicochemical properties and molecular modeling. The predominance of hydrophobic amino acids, together with the elevated GRAVY index and predicted α-helical conformation, likely facilitates strong interactions with lipid bilayers, contributing both to antimicrobial activity and to reduced selectivity toward eukaryotic membranes. Similar relationships between elevated hydrophobicity, amphipathic helicity, and erythrocyte toxicity have been widely reported for membrane-active AMPs [58,59].

Although Peptide 5764 demonstrated antibacterial and particularly notable antibiofilm activity against *P. aeruginosa*, its considerable hemolytic activity at antibacterial concentrations indicates that therapeutic application would require optimization of membrane selectivity. Potential strategies include modulation of hydrophobicity, incorporation of helix-disrupting residues, or adjustment of the cationic/hydrophobic balance. In addition, synergistic interactions with conventional antibiotics could improve the therapeutic applicability of this peptide by reducing the concentrations required for antibacterial activity. Peptide 5764 should therefore be regarded as a validated antimicrobial scaffold for future development rather than a direct drug candidate. The experimental confirmation of activity for this genome-mining-derived peptide provides proof-of-concept support for the approach; the remaining predicted AMP candidates identified in this study require independent experimental validation before any conclusions about their antimicrobial activity can be drawn.

Among the remaining predicted AMP candidates, peptides 3 and 7 showed no detectable homology to characterized sequences in the APD3 database and exhibited favorable physicochemical profiles. Exploratory molecular docking analyses, presented here strictly as hypothesis-generating tools with no claim of demonstrated biological activity, suggested that peptide 3 may interact with the FsrA quorum-sensing regulator of *E. faecium*, and peptide 7 with the LasR regulator of *P. aeruginosa*, through combinations of electrostatic, hydrogen bond, and hydrophobic contacts. These predicted interactions warrant experimental follow-up but do not constitute evidence of antimicrobial or antibiofilm function.

The experimental confirmation of activity for Peptide 5764 closes the analytical loop from genome sequence to functional evidence: a peptide identified computationally from an uncharacterized Antarctic lineage, selected based on physicochemical criteria derived from its genomic sequence, and validated experimentally against clinically relevant pathogens. This outcome directly supports the premise that genome-guided AMP prediction can serve as a productive discovery strategy for lineages where expression-based approaches are hampered by cryptic BGC regulation—precisely the situation encountered in GB-02. The remaining seven computationally predicted AMP candidates identified in this study require independent experimental validation before any conclusions about their antimicrobial activity can be drawn.

## 4. Materials and Methods

### 4.1. Culture and DNA Extraction of an Antarctic Bacterial Strain

An Antarctic bacterial strain (ID GB-02), previously isolated from soil, was used in this study. The soil sample was collected from Greenwich Island (Prat Base) during the 53rd Chilean Antarctic Expedition (ECA), organized by the Chilean Antarctic Institute (INACH). For bacterial isolation, 100 mg of soil was suspended in 1 mL sterile distilled water (final volume) and vigorously stirred by vortexing (30 s). After that, 10 μL of the suspension was inoculated into 990 μL of Luria–Bertani (LB) broth (Tryptone 10.0 g/L, yeast extract 5.0 g/L, and sodium chloride 10.0 g/L) and incubated at 15 °C for 24 h. Subsequently, aliquots were spread onto LB agar plates (bacteriological agar 15.0 g/L, tryptone 10.0 g/L, sodium chloride 10.0 g/L, and yeast extract 5.0 g/L) and incubated at the same temperature for seven days. Pure bacterial cultures were obtained by successive transfers of single colonies to LB agar and then cryopreserved at −80 °C with 20% glycerol.

The culture for DNA extraction was obtained by inoculating the isolated bacterial strain on LB agar at 15 °C for 7 days. Genomic DNA extraction was performed using the ZymoBIOMICS™ DNA Extraction Kit (D4300, ZYMO RESEARCH, Irvine Tustin, CA, USA) following the manufacturer’s protocol. DNA concentration was quantified with a Qubit dsDNA HS Assay Kit (Thermo Fisher Scientific, Waltham, MA, USA), ensuring a minimum concentration of ≥50 ng/µL. DNA integrity was evaluated by electrophoresis on a 0.9% (*w*/*v*) agarose gel prepared in 1× TAE buffer.

*Rhodococcus* sp. GB-02 was handled under BSL-2 conditions following standard laboratory procedures. Genomic analysis using the Comprehensive Antibiotic Resistance Database (CARD) [60] revealed no known virulence factors.

### 4.2. Library Preparation and Whole Genome Sequencing

The genome sequencing was carried out using a hybrid approach combining Illumina and Oxford Nanopore Technologies (ONT) platforms. The Illumina library was prepared with paired-end 150 bp reads using the Nextera XT DNA Library Prep Kit (Illumina, San Diego, CA, USA) and sequenced on the Illumina NovaSeq system. ONT sequencing was carried out in the Scientific and Technological Bioresource Nucleus (BIOREN), Universidad de La Frontera, Chile. The ONT library was prepared using the Rapid Sequencing kit SQK-RBK004, according to the manufacturer’s recommendations, and sequenced on the MinION Mk1C platform using a R9.6 flow cell. The basecalling of the resulting reads was conducted with Guppy v5.0.12 in fast mode (MinKNOW software v.4.3.7).

### 4.3. Hybrid Genome Assembly

Assembly was conducted using both Illumina and ONT sequencing datasets. Illumina read quality was initially assessed with FastQC v0.11.9 [61] followed by adapter trimming and quality filtering using Fastp v0.20.0 with default settings [62]. ONT reads were evaluated with Nanoplot v1.40.0 [63], adapters were removed using Porechop v0.2.4, and reads were further quality filtered (Q > 10) with Nanofilt v2.8.0 [63]. For de novo hybrid assembly, high-quality short and long reads were assembled using Unicycler v0.4.8 [64], and the resulting assemblies were polished with Bowtie2 v2.4.2 and Pilon v1.24 [65,66]. Finally, the quality and potential contamination of the refined hybrid genomes were assessed using CheckM v1.1.3 [67]. The genome assembly generated in this study is available in the National Center for Biotechnology Information (NCBI) under the Bioproject accession number PRJNA1294779.

### 4.4. Phylogenomic Analysis

To determine the phylogenetic position and relationships of GB-02, all available genome assemblies for the type strains of each species of the genus *Rhodococcus* with status name ‘correct’ in the LPSN repository (https://lpsn.dsmz.de/genus/rhodococcus (accessed on 15 February 2026)) were retrieved and included in a phylogenomic analysis performed using the Type Strain Genome Server (TYGS) [68] (https://tygs.dsmz.de (accessed on 15 February 2026)). This tool uses an integrative approach based on the MASH algorithm [69] for genome phylogeny, combined with 16S rRNA phylogeny and digital DDH estimations. Closely related genomes were initially identified using the MASH algorithm. Subsequently, 16S rRNA gene sequences were extracted and compared using BLAST v2.16.0 (NCBI) to confirm phylogenetic affiliations [70]. All pairwise comparisons among all genomes were conducted using the Genome BLAST Distance Phylogeny approach (GBDP), and accurate intergenomic distances were inferred under the algorithm ‘trimming’ and distance formula d5 [71]. Afterwards, a balanced minimum evolution tree was obtained via FASTME 2.1.6.1, including SPR postprocessing [72] and branch support based on 100 pseudo-bootstrap. We used a 70% dDDH radius for species clustering, and a 79% dDDH threshold for subspecies clustering [73]. Average Nucleotide Identity (ANI) was calculated using the JSpeciesWS Online Service [74] (https://jspecies.ribohost.com/jspeciesws/ (accessed on 17 February 2026)). Finally, the Interactive Tree Of Life (iTOL) v5 [75] (https://itol.embl.de/ (accessed on 17 February 2026)) was used for the phylogenomic tree construction. To further evaluate the genomic relatedness of strain GB-02 beyond type material, ANI-based comparative analysis was conducted, including all publicly available *Rhodococcus* genome assemblies deposited in NCBI. Genomes were filtered according to assembly level (contig, scaffold, chromosome, and complete genome) and assembly quality, retaining only those with ≤100 contigs for downstream analyses. In addition, an ANI comparison of the GB-02 genome was carried out using a collection of Antarctic *Rhodococcus* strains to contextualize GB-02 within environmentally related strains.

### 4.5. Genome Annotation and Metabolic Reconstruction

Genome annotation was performed using the Bakta web server v1.11.0 [76,77] and a circular map of GB-02 was constructed using the PROKSEE server v1.1.2 [78], providing a visual overview of its genomic features. Functional categories based on the COG (Clusters of Orthologous Groups) classification were obtained using the online version of eggNOG-mapper v.2 [79]. Furthermore, metabolic pathways were reconstructed using the integrative IA-based tool Ibis v3.1 [80] through the “complete genome annotation” protocol. In addition, data were integrated with pathway information from the Kyoto Encyclopedia of Genes and Genomes (KEGG) [81] and iPath3.0 tool [82] was used to compare the GB-02 metabolic pathways with the closest species *Rhodococcus erythropolis* IEGM 1415 (GCF_027270535.1) and *Rhodococcus* sp. IEGM 1318 (GCF_033142465.1).

### 4.6. BGCs and Antimicrobial Peptides Predictions

The antiSMASH v7.1.0 was used to predict biosynthesis-related gene clusters based on a secondary metabolic analysis with relaxed detection parameters, including the following prediction features: “KnownClusterBlast”, “MIBiG cluster comparison”, “Cluster Pfam analysis”, “TFBS Analysis”, “ClusterBlast”, “ActiveSiteFinder”, “Pfam-based GO term annotation”, “SubClusterBlast”, “RREFinder”, and “TIGRFam analysis” [83]. In addition, genes encoding bacteriocins and ribosomally synthesized and post-translationally modified peptides (RiPPs) were identified using BAGEL 4 [84]. In addition, a BGCs comparison was carried out using a genome collection of *Rhodococcus* type strains, Antarctic *Rhodococcus,* and the two non-type *Rhodococcus* genomes identified as the closest available genomes in databases (Table S4).

Furthermore, small peptides in the genome of GB-02 not covered in the previous analyses were identified. To further explore the biosynthetic potential of *Rhodococcus* sp. GB-02, small peptides (≤100 amino acids) were analyzed to identify potential antimicrobial peptides (AMPs) that were not detected in the previous BGC analysis. The prediction was performed using the complementary tools AI4AMP [85] (http://symbiosis.iis.sinica.edu.tw/PC_6/ (accessed on 5 January 2026)), CAMPR4 [86] (http://camp.bicnirrh.res.in/ (accessed on 6 January 2026)), and AMPA [87] (https://tcofee.crg.cat/apps/ampa/do (accessed on 10 January 2026)), which employ artificial intelligence and physicochemical property-based models to predict and classify AMPs. The criteria for a positive conclusion for possible antimicrobial activity prediction in AI4AMP, CAMPR4, and AMPA were defined as >0.5 in AI4AMP, >0.5 in CAMPR4, and >0.225 in AMPA. In addition, the APD3 database [88] (https://aps.unmc.edu/) was queried to determine whether the identified peptides correspond to previously reported sequences or represent novel AMPs. Finally, the structures of the predicted AMPs were modeled using the SWISS-MODEL [89] (https://swissmodel.expasy.org/ (accessed on 3 February 2026)), PEP-FOLD 3 [90] (http://bioserv.rpbs.univ-paris-diderot.fr/services/PEP-FOLD3 (accessed on 3 February 2026)), and PPM 3.0 [91] (https://opm.phar.umich.edu/ppm_server3) servers, and subsequently visualized with PyMOL Molecular Graphics System v3.0 (Schrödinger, LLC, New York, NY, USA).

Finally, a molecular docking approach was carried out for the predicted extracellular AMP and established protein therapeutic targets. Proteins used in this study were associated with biofilm formation in ESKAPEE pathogens and were obtained from [92].

Docking analysis was performed using the HDOCK web server v1.1 [93], which applies a hierarchical algorithm to predict protein-peptide binding modes based on their three-dimensional structures. Protein structures were retrieved from the RCSB PDB, UniProt, or AlphaFold databases using the same IDs published in [94] while the previously modeled AMP structures were prepared as input files.

The structural analysis and interaction characterization of the protein-peptide complexes were conducted through a multi-software pipeline. The best docking models were obtained from HPEPDOCK and merged using PyMOL v 3.1.6 (https://pymol.org/2/ (accessed on 8 February 2026)). Subsequently, the molecular interactions within the most specific AMPs-protein models were mapped using the MAPIYA server [95] using a cutoff of 3 Ångströms to specifically highlight electrostatic and hydrophobic interaction patterns.

### 4.7. Antibacterial Activity Screening

Crude extracts from *Rhodococcus* sp. GB-02 were evaluated for antibacterial activity against representative Gram-positive and Gram-negative bacteria. The assay was performed to preliminarily assess the antimicrobial potential of the strain. Detailed experimental procedures and data are provided in the Supplementary Materials (Supplementary Methods).

### 4.8. Peptide Synthesis and Purification

Among the predicted antimicrobial peptides, peptide 4, hereafter referred to as peptide 5764, was selected for experimental validation due to its lack of homology with known antimicrobial peptides in the APD3 database and its favorable physicochemical characteristics. Peptide 5764 was chemically synthesized by Fmoc/tBu solid-phase synthesis (Rink amide resin, Iris Biotech GmbH, Marktredwitz, Germany) using a Liberty Blue automatic synthesizer (CEM Corporation, Matthews, NC, USA). The peptide was cleaved with TFA:TIS:EDT:H_2_O (92.5:2.5:2.5:2.5, *v*/*v*), and then purified using a preparative Clean-Up^®^ CEC18153 C-18 column (UCT, Bristol, PA, USA) eluted in a 0 to 100% (*v*/*v*) acetonitrile/water gradient. The peptide was analyzed by RP-HPLC (Waters Corp., Milford, MA, USA, XBridgeTM BEH C18, 0–70% ACN *v*/*v* gradient) and confirmed by electrospray ionization mass spectrometry, ESI-MS (Shimadzu, Kyoto, Japan). The purity of the peptide was higher than 95%. The peptide was lyophilized and stored until use [96].

### 4.9. Bacterial Strains

Gram-positive bacteria, including *Staphylococcus aureus* ATCC 25923, *S. aureus* clinical isolate (STA003), and *Enterococcus faecium* clinical isolate (EN201), as well as Gram-negative bacteria, including *Escherichia coli* ATCC 25922, *Pseudomonas aeruginosa* ATCC 27853, *P. aeruginosa* clinical isolate, *Klebsiella pneumoniae* ATCC 13883, and *K. pneumoniae* clinical isolate (KPN361), were selected to evaluate the antibacterial activity of the peptide. Reference strains and clinical isolates were provided by the Pharmaceutical and Cosmetic Bioproducts Laboratory, Center of Excellence in Translational Medicine, Faculty of Medicine, Universidad de La Frontera. All strains were recovered from stock cultures preserved at −80 °C in Tryptic Soy Broth (TSB; Merck, Darmstadt, Germany) supplemented with 20% glycerol.

### 4.10. The Peptide 5764 Antibacterial Test

The minimum inhibitory concentration (MIC) of the peptide was determined by the broth microdilution method in 96-well microplates according to Clinical and Laboratory Standards Institute guidelines [97]. Briefly, 150 µL of the appropriate culture medium was added to each well, using Brain Heart Infusion (BHI) broth for strain EN 201 and Mueller–Hinton broth (MHB; Merck, Darmstadt, Germany) for the remaining strains. The peptide was dissolved in ultrapure water containing 5% acetic acid to obtain a 5 mM stock solution. Subsequently, 50 µL of the peptide solution was added to the first well, and two-fold serial dilutions were performed to obtain final concentrations ranging from 195 to 0.4 µM. A vehicle control containing the peptide solvent was included under the same experimental conditions.

Bacterial inocula prepared from refreshed overnight cultures were adjusted to approximately 1 × 10^7^ CFU/mL, and 5 µL was added to each well. Ciprofloxacin (CIP) and Tobramycin (TOB) were used as reference antibiotics at concentrations ranging from 256 to 0.5 µg/mL. Positive and negative controls consisted of inoculated culture medium and sterile medium, respectively. Plates were incubated at 37 °C for 16–18 h, and MIC values were determined by visual inspection of bacterial growth inhibition.

For determination of the minimum bactericidal concentration (MBC), 5 µL aliquots from wells showing no visible growth were plated in triplicate onto solid culture medium and incubated at 37 °C for 16–18 h. The lowest concentration showing no colony formation was considered the MBC.

### 4.11. Antibiofilm Assay

The antibiofilm activity of the peptide was evaluated using the crystal violet staining method as previously described [98], with minor modifications. This assay is one of the most widely used methods for in vitro biofilm evaluation due to its simplicity and adaptability [99]. In this study, bacterial suspensions prepared from refreshed overnight cultures were inoculated into 96-well microplates containing Tryptic Soy Broth (TSB; Merck, Darmstadt, Germany) supplemented with 1% glucose to promote biofilm formation. Treatments were evaluated at concentrations corresponding to the MIC and 2 × MIC values of the peptide. A vehicle control containing the peptide solvent at concentrations equivalent to those used for the MIC and 2 × MIC peptide treatments was included under the same experimental conditions. Reference antibiotics, including CIP and TOB, were tested at concentrations corresponding to their respective MIC values and, when applicable, at 1/2 × MIC or 2 × MIC. For strains in which the MIC exceeded or was below the evaluated concentration range, the highest or lowest tested concentration was used, respectively.

After incubation at 37 °C for 24 h under shaking conditions, planktonic cells were removed, and wells were washed three times with phosphate-buffered saline (PBS) to eliminate non-adherent bacteria. Biofilms were fixed with 100% methanol for 15 min and stained with crystal violet solution for 5 min. Excess stain was removed by washing with PBS, and the retained dye was solubilized with 96% ethanol under shaking for 30 min. Biofilm biomass was quantified by measuring the optical density at 595 nm using an Infinite^®^ M200 PRO microplate reader (Tecan, Maennedorf, Switzerland).

The optical density cut-off value (ODc) was determined by summing “mean absorption of the negative control” and “triple deviation of absorption standard of the negative control”. Biofilm production was classified as follows: no biofilm producer (OD ≤ ODc), weak biofilm producer (ODc < OD ≤ 2 × ODc), moderate biofilm producer (2 × ODc < OD ≤ 4 × ODc), and strong biofilm producer (OD > 4 × ODc). The percentage of biofilm inhibition was calculated according to the following equation:

$$\mathrm{Biofilm}\;\mathrm{inhibition}\;(\%)=\frac{\left[\left(OD_{B}-OD_{C})-(OD_{T}-OD_{C}\right)\right]}{\left(OD_{B}-OD_{C}\right)}\times 100$$

where $OD_{B}$ corresponds to the untreated biofilm control, $OD_{C}$ to the negative control, and $OD_{T}$ to the treated sample.

### 4.12. Peptide Hemolysis Assay

To analyze the hemolytic activity of the peptide 5764, 3 mL of the human blood sample was collected in a K2EDTA tube and centrifuged at 5000 rpm for 5 min. After removal of the supernatant, the RBC pellet was washed three times with PBS. The final pellet was resuspended in PBS to obtain a 2% RBC suspension. Aliquots of the RBC suspension (50 µL) were incubated with peptide concentrations ranging from 0.4 to 195 µM (0.4, 1, 2, 3, 6, 12, 24, 49, 98, and 195 µM). A vehicle control corresponding to the peptide solvent was included under the same experimental conditions. PBS and 0.1% Triton X-100 were used as negative and positive controls, respectively. Samples were incubated at 37 °C for 15 min and subsequently centrifuged at 3000 rpm for 5 min. The optical density of the supernatants was measured at 540 nm using an Infinite M200 PRO microplate reader (Tecan, Männedorf, Switzerland).

The percentage of hemolysis was calculated using the following equation:

$$\mathrm{Hemolysis}\;(\%)=\frac{OD_{test}-OD_{PBS}}{OD_{Triton\;X\text{-}100}-OD_{PBS}}\times 100$$

### 4.13. Statistical Analysis

All experiments were performed in triplicate (*n* = 3), and data are presented as mean ± SD. Statistical analyses were conducted using GraphPad Prism 9 software. One-way or two-way ANOVA was used as appropriate, followed by post hoc multiple comparison tests. Tukey’s test was applied for pairwise comparisons among treatments, while Šídák’s multiple comparisons test was used for specific comparisons in selected datasets. In hemolysis assays, Dunnett’s test was applied using the negative control as reference. Statistical significance was considered at *p* < 0.05.

## 5. Conclusions

Comprehensive genomic analyses confirmed that *Rhodococcus* sp. GB-02 belongs to the genus *Rhodococcus* and likely represents a genomic species not yet formally described. Although species-level thresholds were not reached against available type strains, GB-02 showed high genomic similarity to two non-type genomes, suggesting a distinct lineage within the genus. The genome displayed high completeness, broad metabolic versatility, and a complex regulatory network.

Genome mining revealed 20 biosynthetic gene clusters, mainly non-ribosomal peptide synthetases, several of which showed low similarity to known pathways in the MIBiG database, indicating a biosynthetic repertoire that warrants further experimental investigation but for which novelty cannot be confirmed without product-level characterization. Antimicrobial assays of the crude extract revealed measurable but limited activity under the evaluated conditions, supporting the presence of bioactive compounds within the extract while suggesting that additional optimization of cultivation conditions or chemical characterization is required to identify the responsible metabolites.

Multiple putative antimicrobial peptides were predicted; three candidates lacking homology in current databases were selected for exploratory molecular docking analyses, which identified candidate interactions with quorum-sensing and biofilm-associated proteins as hypotheses for future experimental testing. However, these analyses do not constitute evidence of antimicrobial function for these peptides. Experimental validation demonstrated that Peptide 5764 inhibited clinically relevant Gram-positive and Gram-negative bacteria and reduced biofilm formation in a concentration-dependent manner, particularly in *P. aeruginosa*. However, its considerable hemolytic activity at antibacterial concentrations indicates that optimization of membrane selectivity is required before therapeutic application can be considered. Peptide 5764 should therefore be regarded as a validated antimicrobial scaffold rather than a direct drug candidate. The remaining seven computationally predicted AMP candidates identified in this study have not been experimentally tested, and their antimicrobial activity remains to be established through independent validation.

Future research directions include: (i) scaffold optimization of Peptide 5764 through sequence engineering, modulation of hydrophobicity, and adjustment of the cationic/hydrophobic balance to improve selectivity; (ii) experimental validation of the remaining predicted AMP candidates; (iii) activation of cryptic BGCs through targeted strategies informed by the regulatory architecture identified in GB-02—specifically, inactivation of TetR/ArsR local repressors, overexpression of LuxR-type and LysR-type activators, and manipulation of ECF sigma factors—under conditions that mimic the competitive pressures of Antarctic soil environments. Together, these findings establish *Rhodococcus* sp. GB-02 as an Antarctic lineage with a diverse and largely unexplored biosynthetic repertoire and demonstrate the value of integrating genome-guided analyses with experimental validation to uncover antimicrobial potential in previously undescribed *Rhodococcus* lineages.

## Figures and Tables

**Table 1:** dDDH and ANI analyses of strain GB-02 against closely related *Rhodococcus* genomes, including type and Antarctic strains.

Strain	Closest Genome (TYGS)	dDDH (%)	ANI (%)
GB-02	*Rhodococcus erythropolis* JCM 3201 ^T^	54.3	93.11
	*Rhodococcus antarcticus* 75 ^T^*	54.3	91.30
	*Rhodococcus* sp. ADH *	54.7	91.03
	*Rhodococcus* sp. NJ-530 *	54.0	91.01
	*Rhodococcus qingshengii* Ray *	54.3	90.98
	*Rhodococcus* sp. WY5 *	54.9	90.96
	*Rhodococcus qingshengii* A29-k1 *	54.2	90.96
	*Rhodococcus* sp. AQ5-07 *	54.0	90.92
	*Rhodococcus globerulus* NBRC 14531 ^T^	23.8	79.67
	*Rhodococcus opacus* DSM 43205 ^T^	20.5	73.78
	*Rhodococcus defluvii* Ca11 ^T^	20.2	72.80

^T^: type strain, *: Antarctic strain.

**Table 2:** Predicted secondary metabolite biosynthetic gene clusters in strain GB-02 identified by antiSMASH.

Region ID	Type	From (bp)	To (bp)	Regulatory Genes	Most Similar Known Cluster		Similarity
1.1	RiPP-like	272,228	284,158	AsnC family transcriptional regulator	Branched-chain fatty acids	Other	75%
1.2	LAP	434,515	464,622	LuxR family DNA-binding response regulator Sensor histidine kinase TetR family transcriptional regulator	Diisonitrile antibiotic SF2768	NRP	11%
1.3	T1PKS	554,979	599,928	GntR family transcriptional regulator Autoinducer-binding transcriptional regulator LysR family transcriptional regulator	N/A	N/A	N/A
1.4	NAPAA	884,871	918,764	GntR family transcriptional regulator	ε-Poly-L-lysine	NRP	100%
1.5	NRP-metallophore, NRPS	1,280,099	1,338,272	Response regulator Sensor histidine kinase	Erythrochelin	NRP	57%
1.6	Redox-cofactor	2,111,967	2,134,764	LuxR family DNA-binding response regulator TetR family transcriptional regulator	Tetronasin	Polyketide	3%
1.7	NRPS	2,150,479	2,248,839	TetR family transcriptional regulator	CorynecinIII/ corynecinI/ corynecin II	Other	100%
1.8	NRPS-like	2,639,305	2,681,839	TetR family transcriptional regulator	N/A	N/A	N/A
2.1	Butyrolactone	1,290,675	1,301,562	TetR family transcriptional regulator ArsR family transcriptional regulator	N/A	N/A	N/A
2.2	Lanthipeptide-class-iii	1,509,634	1,524,266	TetR family transcriptional regulator	N/A	N/A	N/A
3.1	NRPS	126,273	192,834	Sensor histidine kinase LuxR family DNA-binding response regulator ArsR family transcriptional regulator	Coelichelin	NRP	27%
3.2	NRPS, terpene	216,265	270,544	TetR family transcriptional regulator	SF2575	Polyketide: Type II polyketide + Saccharide: Hybrid/tailoring saccharide	6%
3.3	NRP-metallophore, NRPS	603,001	660,933	RNA polymerase, sigma-24 subunit, ECF subfamily LysR family transcriptional regulator AraC family transcriptional regulator Sigma-54-dependent transcriptional regulator	Heterobactin B/Heterobactin S2	NRP	100%
4.1	NRPS	90,858	157,668	Sensor histidine kinase LuxR family DNA-binding response regulator	Rifamorpholine A–E	Polyketide	3%
4.2	hglE-KS	164,549	210,941	LysR family transcriptional regulator	Petrichorin A/B	NRP	2%
4.3	NRPS	267,756	312,129	TetR family transcriptional regulator Sensor histidine kinase Response regulator ArsR family transcriptional regulator	N/A	N/A	N/A
4.4	NRPS	314,271	370,506	AraC family transcriptional regulator LysR family transcriptional regulator TetR family transcriptional regulator	Monensin	Polyketide	5%
4.5	Terpene	449,372	470,313	Serine/threonine protein kinase	Isorenieratene	Terpene	42%
4.6	Arylpolyene/terpene-precursor	555,200	596,282	TetR family transcriptional regulator	Aurachin RE	Terpene + Polyketide	100%
4.7	Ectoine	766,781	777,179	Transcriptional regulator, MerR family	Ectoine	Other: Ectoine	75%

N/A: not available.

**Table 3:** Physicochemical characteristics and BLAST results of predicted peptides in strain GB-02 against the APD3 database.

Sequence		Aa No.	+ aa	– aa	Mol. Wt (kDa)	APD (%)	Net Charge	GRAVY	Hydroph. (%)	Similarity/APD ID	Peptide Name	Organism	Activity
1	LFWAFGFGILLGAGAITTGIFALRRHPRKSGGHLLSNARQLPWDAIVGL	49	7	1	5.274	51	+4.5	0.502	51	32.31%/AP00932	Acidocin B	*Lactobacillus acidophilus* M46	Anti-Gram+ & −
2	IVPATVVASAIVAIAWVVRSGHPSGAPLGHGKVR	34	5	0	3.374	53	+3.2	0.818	52.9	38.24%/AP03163	Peptide 5	*Lactobacillus fermentum* strain HF-D1	Anti-Gram–
3	IVTVVVLCIGGVLAVSMQVRCVDAAREAARLVARGDRESAVATARRIAP	49	7	4	5.091	59	+3	0.755	59.2	N/A	N/A	N/A	N/A
4	VATLVLGAFLLGWRALAPLVVKIRTKS	27	4	0	2.894	63	+4	1.178	63	N/A	N/A	N/A	N/A
5	AIGGLAGPTRAGKPLCRRILVIVSGVFAVLQIALVVAGISTVLALVVPV	49	4	0	4.850	63	+4	1.590	63.3	33.33%/AP01168	Carnocyclin A	*Carnobacterium maltaromaticum* UAL307	Anti-Gram+
6	GPGTTSITAHIAASVVALAAQIWCDRRRGIAAVAGSVVVLVVAGLLLVT	49	4	1	4.826	61	+2.25	1.263	61.2	32.26%/AP04500	Avidumicin	*Cutibacterium avidum*	Anti-Gram+
7	PLGITILLSAIAGALIMALAGGVRIIQIRRAAKRQM	36	5	0	3.758	61	+5	1.042	61.1	N/A	N/A	N/A	N/A
8	ELVKVATYVLAFVVGMQKSTLKRAS	25	4	1	2.739	52	+3	0.584	56	38.46%/AP03315	SyCPA 153	*Brevibacillus brevis* NBRC 100599	Anti-Gram+, MRSA

Total number of amino acids (Aa No.), number of positively (+ aa) and negatively (− aa) charged residues, molecular weight (Mol. Wt in kDa), amino acid percentage distribution (APD), hydropathicity index (GRAVY), hydrophobic residue content (Hydroph. %), similarity to known peptides from the APD3 database with their corresponding ID (APD ID), not available in APD3 database (N/A), and Methicillin-Resistant *Staphylococcus aureus* (MRSA). Peptide charges were calculated at pH 7.0, reflecting physiological conditions. The contribution of histidine residues to the net charge may vary due to its pK ≈ 7.

**Table 4:** Minimum inhibitory concentration (MIC) and minimum bactericidal concentration (MBC) values of Peptide 5764 and reference antibiotics against pathogenic bacterial strains.

Strain	Peptide 5764		Tobramycin	Ciprofloxacin
	MIC (μg/mL)	MBC (μg/mL)	MIC (μg/mL)	MIC (μg/mL)
**EN 201 (clinical isolate)**	284	568	>256	>256
***Staphylococcus aureus* ATCC 25923**	284	568	>256	<0.5
**STA 003 (clinical isolate)**	284	568	>256	2
***Escherichia coli* ATCC 25922**	284	568	8	<0.5
***Pseudomonas aeruginosa* ATCC 27853**	284	568	<0.5	<0.5
***Pseudomonas aeruginosa* (clinical isolate)**	>568	ND	<0.5	<0.5
***Klebsiella pneumoniae* ATCC 13883**	>568	ND	32	16
**KPN 361 (clinical isolate)**	>568	ND	32	16

EN 201: *Enterococcus faecium*, STA 003: *Staphylococcus aureus*, KPN 361: *Klebsiella pneumoniae*; ND: not determined. The symbols “<” and “>” indicate that the MIC was below the lowest or above the highest concentration tested, respectively, and therefore denote the limits of the concentration range evaluated. Peptide concentrations were originally evaluated in micromolar (µM) and subsequently converted to µg/mL according to the peptide molecular weight (2892.6 Da). Accordingly, MIC and MBC values of 284 and 568 μg/mL correspond to approximately 98 and 196 μM, respectively.

## Data Availability

The genome assembly generated in this study is available in the National Center for Biotechnology Information (NCBI) under the Bioproject accession number PRJNA1294779.

## Supplementary Materials

The following supporting information can be downloaded at: https://www.mdpi.com/article/10.3390/ijms27156655/s1.

## Abbreviations

The following abbreviations are used in this manuscript:

AI4AMP	Artificial intelligence–based predictor for antimicrobial peptides
AMP	Antimicrobial peptide
AMPA	Antimicrobial Peptide Analyzer
ANI	Average nucleotide identity
antiSMASH	Antibiotics and Secondary Metabolite Analysis Shell
APE	Arylpolyene
BGC	Biosynthetic gene cluster
BHI	Brain Heart Infusion broth
BiG-SCAPE	Biosynthetic Gene Similarity Clustering and Prospecting Engine
CAMPR4	Collection of Anti-Microbial Peptides database (release 4)
CARD	Comprehensive Antibiotic Resistance Database
CDSs	Coding sequences
CIP	Ciprofloxacin
COG	Clusters of Orthologous Groups
dDDH	Digital DNA–DNA hybridization
ESKAPEE	*Enterococcus faecium*, *Staphylococcus aureus*, *Klebsiella pneumoniae*, *Acinetobacter baumannii*, *Pseudomonas aeruginosa*, *Enterobacter* spp., and *Escherichia coli*
GBDP	Genome BLAST distance phylogeny
GCF	Gene cluster family
HDOCK	Hybrid docking
hglE-KS	Heterocyst glycolipid synthase-like PKS
HPEPDOCK	Hierarchical Peptide–Protein Docking
iTOL	Interactive Tree Of Life
KEGG	Kyoto Encyclopedia of Genes and Genomes
K2EDTA	Dipotassium ethylenediaminetetraacetic acid
LAP	Linear azol(in)e-containing peptide
LB	Luria–Bertani
LPSN	List of Prokaryotic Names with Standing in Nomenclature
MBC	Minimum Bactericidal Concentration
MBF	Moderate biofilm formation
MHB	Mueller–Hinton broth
MIBiG	Minimum Information about a Biosynthetic Gene cluster
MIC	Minimum inhibitory concentration
NAPAA	Non-alpha poly-amino acids
NRPS	Nonribosomal peptide synthetase
ODB	Optical Density of control sample with Bacteria
ODC	Optical Density of Control sample without bacteria
ODT	Optical Density of peptide-Treated sample
ONT	Oxford Nanopore Technologies
PBS	Phosphate-Buffered saline
Pfam	Protein families
PKS	Polyketide synthase
PROKSEE	Prokaryotic Sequence and Expression Explorer
QS	Quorum sensing
RBC	Red Blood Cell
RCSB PDB	Research Collaboratory for Structural Bioinformatics Protein Data Bank
RiPP	Post-translationally modified peptide
ROS	Reactive Oxygen Species
RREs	RiPP Recognition Elements
SAM	S-adenosyl-L-methionine
SBF	Strong biofilm formation
SMs	Secondary metabolites
T1PKS	Type I polyketide synthase
TAE	Tris–Acetate–EDTA
TFBS	Transcription Factor Binding Site
TIGRFam	The Institute for Genomic Research protein families
TOB	Tobramycin
TSB	Tryptic Soy Broth
TYGS	Type Strain Genome Server
WBF	Weak biofilm formation
