# Supplementary material for: Genome-Guided Discovery of an Antimicrobial Peptide Scaffold from an Undescribed Antarctic Rhodococcus Lineage: A Proof-of-Concept Study
Source: Int J Mol Sci. 2026 Jul 25;27(15):6655. doi: 10.3390/ijms27156655 (PMC13467173; doi:10.3390/ijms27156655)
Supplement: Supplementary file 1 [file ijms-27-06655-s001.zip › Supplementary material (ijms-4245959) FINAL.pdf]

Supplementary Materials

Genome-guided discovery of an antimicrobial peptide scaffold from an undescribed Antarctic *Rhodococcus* lineage: a proof-of-concept study

Dayaimi González [1,2,3], Andrés Santos [2,4,5], Eulàlia Sans-Serramitjana [2,3,6], Fanny Guzmán [7], Carla Gallardo-Benavente [8], Dayani Hernández [9], Mercyleidi Díaz Reyes[10], and Francisca Acevedo [2,3,4,*]

[1] Programa de Doctorado en Ciencias Mención Biología Celular y Molecular Aplicada, Universidad de La Frontera, Temuco 4811230, Chile; d.gonzalez47@ufromail.cl (D.G.)

[2] Center of Excellence in Translational Medicine – Scientific and Technology Bioresource Nucleus (CEMT- BIOREN), Faculty of Medicine, Universidad de La Frontera, Avenida Alemania N° 0458, Temuco, Chile; andres.santos@ufrontera.cl (A.S.); eulalia.sans@ufrontera.cl (E.S-S.)

[3] Millennium Nucleus Bioproducts, Genomics and Environmental Microbiology (BioGEM), Avenida España 1680, 2390123, Valparaíso, Chile

[4] Department of Basic Sciences, Faculty of Medicine, Universidad de La Frontera, Casilla 54-D, Temuco, Chile

[5] Center of Biomedical Informatics, Faculty of Medicine, Universidad de La Frontera, Avenida Alemania N° 0458, Temuco, Chile

[6] Department of Integral Adult Dentistry, Faculty of Dentistry, Universidad de La Frontera, Avenida Francisco Salazar 01145, Temuco, Chile

[7] Curauma Biotechnology Center, Pontificia Universidad Católica de Valparaíso, Valparaíso 2362807, Chile; fanny.guzman@pucv.cl (F.G.)

[8] Biotechnology Center for Microbial Studies (CEBEM), Universidad de La Frontera, Temuco 4811230, Chile; carlagallardo6@gmail.com (C.G-B.)

[9] Departamento de Cirugía General, Facultad de Medicina de Mayabeque, Universidad de Ciencias Médicas de La Habana, Mayabeque 32000, Cuba; dayanihernandezreyes@gmail.com (D.H.)

[10] Instituto de Biología Molecular y Celular de Rosario (IBR-CONICET), Universidad Nacional de Rosario (UNR), 2000 Rosario, Argentina; diazreyes@ibr-conicet.gov.ar (M.D.R.)

* Correspondence: francisca.acevedo@ufrontera.cl (F.A.)

Supplementary Methods

Antibacterial activity screening of organic extracts from strain GB-02

The GB-02 strain was reconstituted onto LB agar plates at 15 °C for one week and used to inoculate 10 mL of LB broth, incubated at 15 °C with shaking at 120 rpm as a starter culture. The culture was then scaled up to 100 and 250 mL under the same conditions to obtain sufficient biomass for crude extract preparation.

Organic crude extracts were obtained from bacterial cultures by solvent extraction with ethyl acetate. The mixtures of crude culture:solvent (1:1 ratio) were vigorously shaken for 10 min and kept stationary from 15 to 30 min until separation of aqueous and organic phases. Organic phases were collected and concentrated in a rotary evaporator (model RE100–Pro; SCILOGEX, LLC, CT, USA) at 40 °C and 80 rpm. The final concentrated extracts (approximately 2 mL) were transferred to pre–weighed tubes and dried at room temperature for at least 24 h. The dried crude extracts were then weighed, dissolved in 200 µL of methanol, and finally stored at −20 °C until assay their antimicrobial activity.

In vitro microbiological assays were conducted to ascertain the minimum inhibitory concentration (MIC) and minimum bactericidal concentration (MBC) of the *Rhodococcus* extract against aerobic bacterial strains (*Escherichia coli* ATCC 25922 and *Staphylococcus aureus* ATCC 25923) using the broth microdilution method in accordance with the Clinical Laboratory Standards Institute (CLSI). MIC and MBC values were determined in triplicate across at least three independent experiments.

Serial dilutions of the *Rhodococcus* extract in culture medium were prepared in 96-well microplates, filling consecutive wells to a final volume of 100 µL. Subsequently, 5 µL of microbial suspensions adjusted to 1.0×10^7 CFU/mL were added to each well. Bacterial suspensions without *Rhodococcus* extract served as controls. Microbial concentrations were estimated by measuring absorbance at 600 nm using an Infinite® M200 PRO microplate reader (Tecan, Maennedorf, Switzerland). The plates were then incubated for 16–18 h at 37°C. After incubation, MIC values were determined by visual inspection of wells for the presence or absence of bacterial growth. The concentrations showing potential microbial inhibition were selected for the MBC assay.

For the MBC assay, 5 µL from MIC wells showing bacterial inhibition were plated onto solid medium in Petri dishes, in triplicate. Plates were incubated for 16–18 h at 37 °C, after which bacterial growth was assessed. Wells showing no bacterial growth were further analyzed. The assay was validated using medium-only controls and positive controls containing bacterial growth.

Rhodococcus extracts were also screened for antimicrobial activity against *E. coli* ATCC 25922 and *S. aureus* ATCC 25923 using the disk diffusion assay (DDA) according to CLSI guidelines. For this purpose, 20 µL of each organic crude extract at varying concentrations (5, 10, 20, 35, and 50 mg/mL) was applied to 6 mm Oxoid™ Blank Antimicrobial Susceptibility Disks (Thermo Fisher Scientific, Waltham, MA, USA), allowed to dry, and then placed on Mueller–Hinton agar plates (BD Difco™, Becton, Dickinson and Company, NY, USA) previously inoculated with bacterial suspensions

adjusted to 0.5 McFarland turbidity (~1.5 × 10^8 CFU/mL). The diameters of inhibition zones were measured after 20 h of incubation at 37 °C.

Supplementary Figures

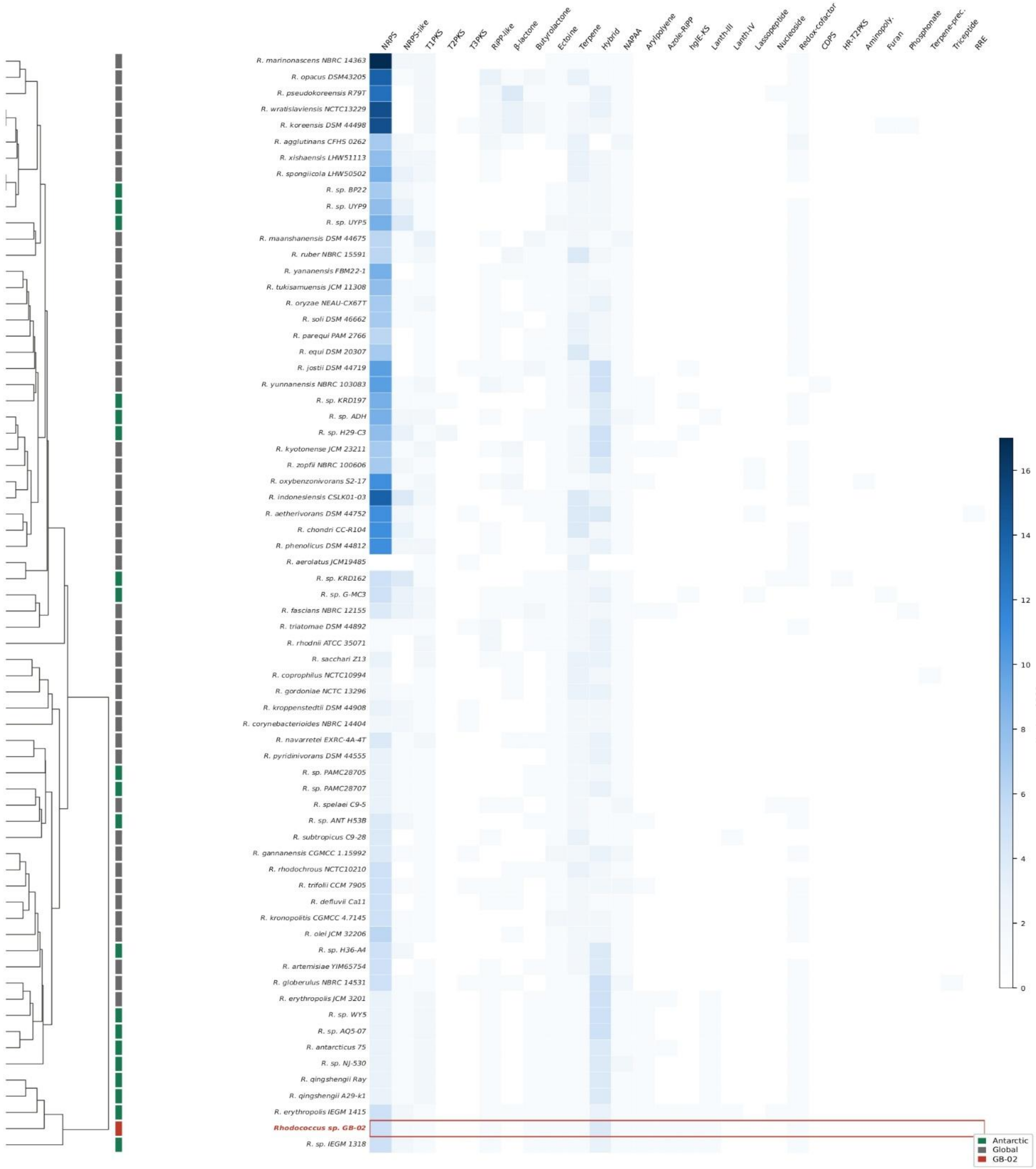

Figure S1. Heatmap showing the distribution of biosynthetic gene cluster (BGC) subclasses across Antarctic and global *Rhodococcus* genomes, with strain GB-02 highlighted in red.

A

AOI	Start	End	Class
**2.0.AOI-01**	1511129	1531798	Lanthipeptide_class_IV
**1.1.AOI-01**	268396	289155	86.3;putative_Bacteriocin_family_protein
**1.1.AOI-02**	444113	464113	LAPs

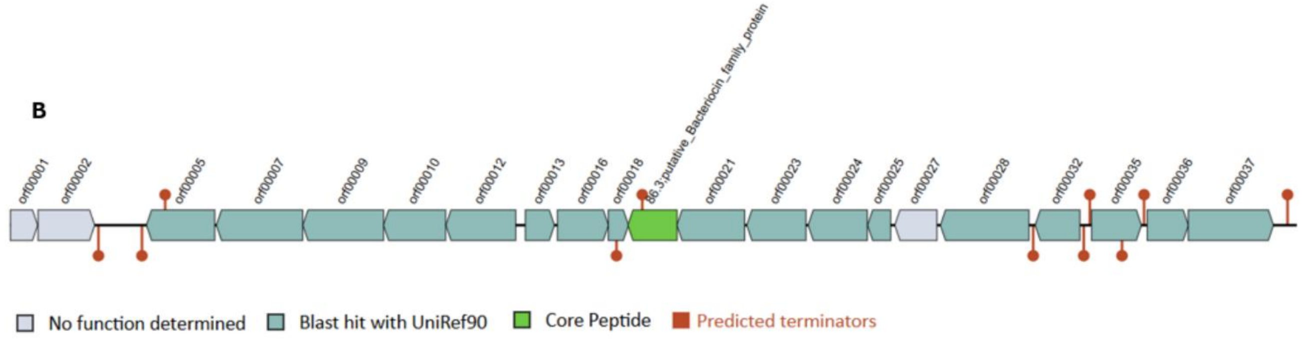

Figure S2. A) List of identified RiPPs in the GB-02 genome using BAGEL4 server. **B)** Bacteriocin cluster in the GB-02 genome. The arrows indicate the transcription direction of each gene.

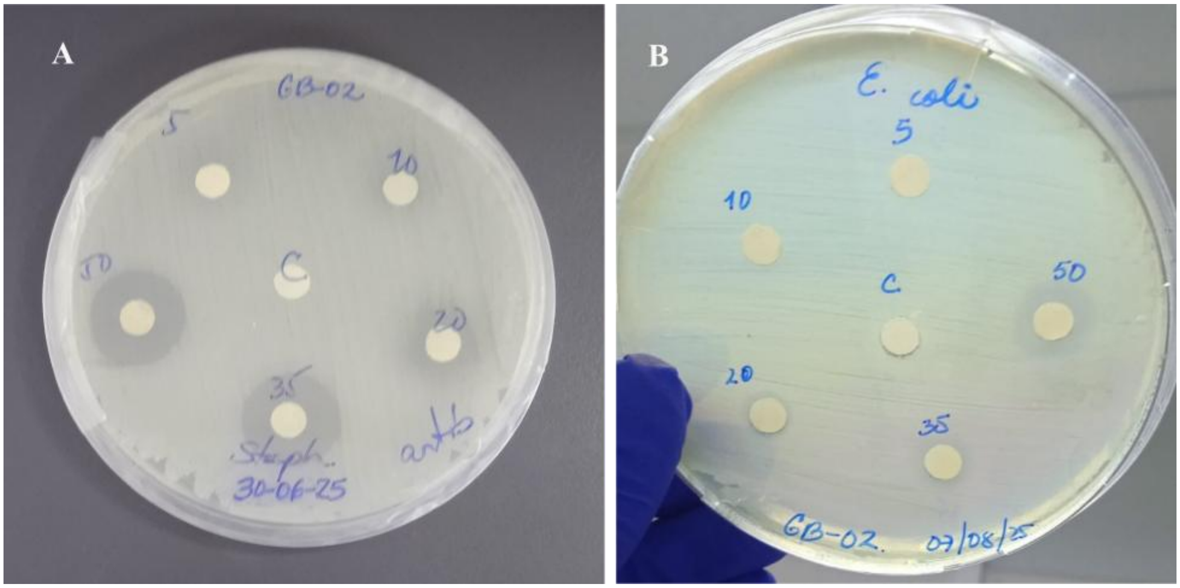

Figure S3. Antimicrobial activity of crude extracts from *Rhodococcus* sp. GB-02 against pathogenic bacteria assessed by disc diffusion assay. Petri dishes show the inhibition zones generated by different concentrations of the extract (5, 10, 20, 35, and 50 mg/mL) against (A) *Staphylococcus aureus* ATCC 6538 and (B) *Escherichia coli* ATCC 22925. The central disc labeled "C" corresponds to the negative control (solvent only).

a

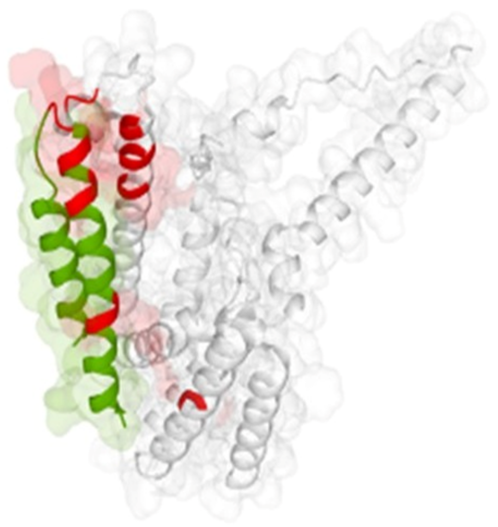

b

FsrA protein *Enterococcus faecium*	Peptide3	Distance (A)	Possible interaction forces
ILE:10	GLN:18	2.982	induction + dispersion
ASP:12	VAL:19	2.151	induction + dispersion
ASP:12	ARG:20	2.562	salt bridge, hydrogen bond
LYS:21	ASP:23	2.686	salt bridge, hydrogen bond
TRP:24	ARG:26	2.171	cation-π stacking
THR:25	ARG:26	1.917	electrostatic: ion-dipole, hydrogen bond
TYR:28	ARG:26	2.431	cation-π stacking,π-π stacking, hydrogen bond
TYR:32	ILE:9	2.592	hydrophobic
TYR:32	VAL:32	2.112	hydrophobic
LEU:36	ILE:9	2.168	hydrophobic
PHE:39	VAL:6	1.937	hydrophobic
LEU:40	VAL:6	2.848	hydrophobic
LEU:43	VAL:2	2.513	hydrophobic
PHE:56	ILE:1	2.407	hydrophobic
ILE:63	ALA:40	2.47	hydrophobic

Figure S4. Molecular interactions between AMP peptide 3 and the FsrA protein of *Enterococcus faecium*. (a) Docking complex representation showing the peptide in green and hydrogen bonds areas in red. (b) Predicted residue-residue interactions and interaction forces within the docking interface.

Specific analysis of the best docking affinities revealed that peptide 3 possess a complex binding interface stabilized by multiple non-covalent forces at distances below 3 Å with the FsrA protein from *E. faecium* reveals (Figure S4). Key polar interactions include a salt bridge and hydrogen bond between ASP:12 of the protein and ARG:20 of the peptide (2.562 Å), and another between LYS:21 and ASP:23 (2.686 Å). Significant cation-π stacking interactions are observed involving TRP:24 and ARG:26 (2.171 Å) as well as TYR:28 and ARG:26 (2.431 Å), the latter also involving π-π stacking and hydrogen bonding. A strong hydrogen bond (1.917 Å) is formed between THR:25 and ARG:26. The interface is further consolidated by extensive hydrophobic contacts, including PHE:39 with VAL:6 (1.937 Å), LEU:36 with ILE:9 (2.168 Å), and PHE:56 with ILE:1 (2.407 Å). This multifaceted interaction profile, combining specific polar/ionic contacts with broad hydrophobic packing, likely underpins the high-affinity binding.

Similarly, peptide 4 established an interaction network with the FimH protein from *E. cloacae* characterized predominantly by short-range hydrophobic contacts (Figure S5). A total of 17 residue-residue interactions were identified, with hydrophobic forces predominating in 12 cases. Interaction distances are relatively short, ranging from 2.227 Å to 2.938 Å. Notably, the TYR:328-THR:3 pair exhibits a complex electrostatic interaction combining a hydrogen bond with dipole-dipole and dipole-π stacking components, being the sole interaction labeled as hydrophilic. Induction and dispersion forces are observed in four pairs, including VAL:206-THR:3 and ASP:209-GLY:7. Additionally, a single case of anion-π stacking is identified between ASP:212 and PHE:9. Collectively, these results indicate a clear predominance of short-range hydrophobic interactions.

Peptide 4 also exhibited a diverse interaction profile with the LasA protein from *P. aeruginosa* (Figure S6). The complex involved 19 residue-residue interactions combining hydrophobic contacts, induction and dispersion forces, and directional electrostatic interactions. Induction and dispersion forces are the most prevalent, occurring in nine pairs such as THR:26-LEU:6 and GLN:66-VAL:5. Several complex electrostatic interactions, including hydrogen bonds and ion-dipole contributions, are observed (e.g., GLU:112-THR:3 and ASN:150-ARG:14), all annotated with a hydrophilic filter. Specialized interactions such as cation-π and π-π stacking are identified in HIS:122-PHE:9, along with dipole-π stacking. Distances are

notably short, reaching a minimum of 1.847 Å for ASN:153-GLY:12. Overall, the results reveal a balance between hydrophobic contacts, induction/dispersion forces, and complex directional electrostatic interactions.

a

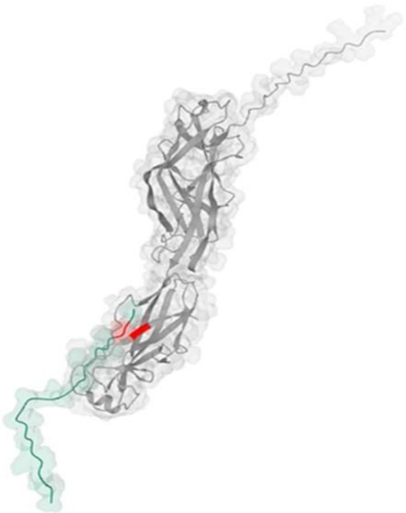

b

FimH *Enterobacter cloacae*	Peptide 4	Distance (A)	Possible interaction forces
ILE:200	ALA:2	2.502	hydrophobic
VAL:206	THR:3	2.565	induction + dispersion
VAL:206	LEU:4	2.625	hydrophobic
PHE:208	LEU:4	2.33	hydrophobic
PHE:208	GLY:7	2.578	hydrophobic
ASP:209	GLY:7	2.342	induction + dispersion
PHE:210	GLY:7	2.663	hydrophobic
GLY:211	PHE:9	2.717	hydrophobic
ASP:212	PHE:9	2.466	anion-π stacking
ILE:213	PHE:9	2.442	hydrophobic
ILE:213	LEU:10	2.75	hydrophobic
ILE:213	LEU:11	2.227	hydrophobic
GLY:214	LEU:11	2.769	hydrophobic
PHE:218	LEU:10	2.905	hydrophobic
SER:325	LEU:6	2.938	induction + dispersion
TYR:328	THR:3	2.921	electrostatic: dipole-dipole, hydrogen bond, dipole-π stacking
LEU:329	ALA:2	2.752	hydrophobic

Figure S5. Molecular interactions between AMP peptide 4 and the FimH protein of *Enterobacter cloacae*. (a) Docking complex representation showing the peptide in green and hydrogen bonds areas in red. (b) Predicted residue-residue interactions and interaction forces within the docking interface.

a

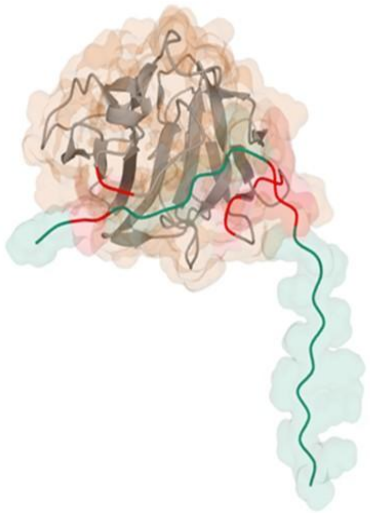

b

LasA *Pseudomonas aeruginosa*	Peptide 4	Distance (A)	Possible interaction forces
THR:26	LEU:6	2.853	induction + dispersion
THR:26	GLY:7	2.831	induction + dispersion
THR:26	ALA:8	2.829	induction + dispersion
GLN:66	VAL:5	2.442	induction + dispersion
GLU:112	THR:3	2.266	electrostatic: ion-dipole, hydrogen bond, interaction filter: hydrophilic
GLU:112	VAL:5	2.813	induction + dispersion
SER:115	GLY:7	2.902	induction + dispersion
SER:115	PHE:9	2.919	dipole-π stacking
THR:117	PHE:9	2.851	dipole-π stacking
HIS:122	PHE:9	2.294	cation-π stacking, π-π stacking
THR:148	LEU:16	2.676	induction + dispersion
ASN:150	ARG:14	2.399	electrostatic: ion-dipole, hydrogen bond, interaction filter: hydrophilic
TYR:151	ALA:8	1.867	hydrophobic
TYR:151	PHE:9	2.58	π-π stacking, hydrophobic, dipole-π stacking
ASN:153	GLY:12	1.847	induction + dispersion
ASN:153	TRP:13	2.408	hydrogen bond, π-π stacking, dipole-π stacking, interaction filter: hydrophilic
ASN:153	ARG:14	2.915	electrostatic: ion-dipole, hydrogen bond, interaction filter: hydrophilic
PHE:172	LEU:11	2.407	hydrophobic
PHE:172	GLY:12	2.523	hydrophobic

Figure S6. Molecular interactions between AMP peptide 4 and the LasA protein of *Pseudomonas aeruginosa*. (a) Docking complex representation showing the peptide in green and hydrogen bonds areas in red. (b) Predicted residue-residue interactions and interaction forces within the docking interface.

The binding interface between peptide 7 and the LasR protein of *P. aeruginosa* is characterized by a network of specific polar and hydrophobic interactions (Figure S7). A reciprocal salt bridge and hydrogen bond pair is established between LYS:42 and ASP:43 of the protein, measured at 2.849 Å and 2.86 Å, respectively. A prominent anion-π stacking interaction, accompanied by π-π stacking and hydrogen bonding, occurs between TRP:152 and ASP:156 (2.85 Å), with a reinforcing salt bridge between ASP:156 and LYS:155 (2.656 Å). An additional salt bridge connects ARG:24 with GLU:168 (2.672 Å). Induction and dispersion forces are observed between VAL:83 and GLN:160 (2.93 Å). Hydrophobic packing is contributed by the interaction between MET:17 and PHE:7 (2.915 Å), which also exhibits dipole-π stacking character.

a

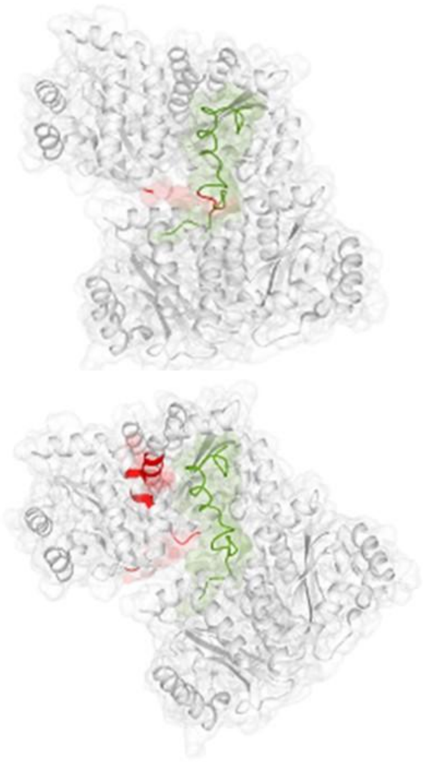

b

LasR *Pseudomonas aeruginosa*	Peptide7	Distance (A)	Possible interaction forces
**LYS:42**	ASP:43	2.849	salt bridge, hydrogen bond
**ASP:43**	LYS:42	2.86	salt bridge, hydrogen bond
**VAL:83**	GLN:160	2.93	induction + dispersion
**TRP:152**	ASP:156	2.85	anion-π stacking,π-π stacking, hydrogen bond
**LYS:155**	ASP:156	2.656	salt bridge, hydrogen bond
**ASP:156**	TRP:152	2.866	anion-π stacking,π-π stacking, hydrogen bond
**ASP:156**	LYS:155	2.762	salt bridge, hydrogen bond
**GLN:160**	VAL:83	2.986	induction + dispersion
**MET:17**	PHE:7	2.915	hydrophobic,dipole-π stacking
**ARG:24**	GLU:168	2.672	salt bridge, hydrogen bond

Figure S7. Molecular interactions between AMP peptide 7 and the LasR protein of *Pseudomonas aeruginosa*. (a) Docking complex representations showing the peptide in green and hydrogen bond regions in red for different LasR chains. (b) Predicted residue-residue interactions and interaction forces within the docking interface.

Supplementary Tables

Table S1. Average Nucleotide Identity (ANI) of *Rhodococcus* sp. GB-02 against high-quality *Rhodococcus* genomes (≤100 contigs) from NCBI.

Query genome	Subject genome	ANI (%)	Aligned fragments	Total query fragments
**GB02.fasta**	GCF_042738325.1_ASM4273832v1_genomic.fna	94,7990	1788	2188
**GB02.fasta**	GCF_015643565.1_ASM1564356v1_genomic.fna	94,2876	1885	2188
**GB02.fasta**	GCF_002501585.1_ASM250158v1_genomic.fna	94,1685	1818	2188
**GB02.fasta**	GCF_008728655.1_ASM872865v1_genomic.fna	94,1385	1872	2188
**GB02.fasta**	GCF_018588185.1_ASM1858818v1_genomic.fna	94,1382	1884	2188
**GB02.fasta**	GCF_015034605.1_ASM1503460v1_genomic.fna	94,1293	1876	2188
**GB02.fasta**	GCF_003444715.1_ASM344471v1_genomic.fna	94,1126	1856	2188
**GB02.fasta**	GCF_016722835.1_ASM1672283v2_genomic.fna	94,1121	1890	2188
**GB02.fasta**	GCF_003797745.1_ASM379774v1_genomic.fna	94,0995	1836	2188
**GB02.fasta**	GCF_017910955.1_ASM1791095v1_genomic.fna	94,0945	1838	2188
**GB02.fasta**	GCF_018729485.1_ASM1872948v1_genomic.fna	94,0791	1832	2188
**GB02.fasta**	GCF_001552595.1_ASM155259v1_genomic.fna	94,0789	1795	2188
**GB02.fasta**	GCF_013277835.1_ASM1327783v1_genomic.fna	94,0717	1822	2188
**GB02.fasta**	GCF_009863335.1_ASM986333v1_genomic.fna	94,0662	1848	2188
**GB02.fasta**	GCF_003860625.1_ASM386062v1_genomic.fna	94,0529	1877	2188
**GB02.fasta**	GCF_010119425.1_ASM1011942v1_genomic.fna	94,0455	1795	2188
**GB02.fasta**	GCF_976989135.1_ASM97698913v1_genomic.fna	94,0450	1870	2188
**GB02.fasta**	GCF_003990875.1_ASM399087v1_genomic.fna	94,0432	1826	2188
**GB02.fasta**	GCF_009707305.1_ASM970730v1_genomic.fna	94,0416	1844	2188
**GB02.fasta**	GCF_018587985.1_ASM1858798v1_genomic.fna	94,0408	1795	2188
**GB02.fasta**	GCF_015678935.1_ASM1567893v1_genomic.fna	94,0396	1816	2188
**GB02.fasta**	GCF_017068035.1_ASM1706803v1_genomic.fna	94,0334	1810	2188
**GB02.fasta**	GCF_018283555.1_ASM1828355v1_genomic.fna	94,0297	1814	2188
**GB02.fasta**	GCF_015099595.1_ASM1509959v1_genomic.fna	94,0234	1844	2188
**GB02.fasta**	GCF_017795725.1_ASM1779572v1_genomic.fna	94,0229	1894	2188
**GB02.fasta**	GCF_008306195.1_ASM830619v1_genomic.fna	94,0210	1841	2188
**GB02.fasta**	GCF_002893965.1_ASM289396v1_genomic.fna	94,0159	1824	2188
**GB02.fasta**	GCF_003313445.1_ASM331344v1_genomic.fna	94,0089	1839	2188
**GB02.fasta**	GCF_017876985.1_ASM1787698v1_genomic.fna	94,0081	1843	2188
**GB02.fasta**	GCF_011290655.1_ASM1129065v1_genomic.fna	94,0018	1867	2188
**GB02.fasta**	GCF_011611865.1_ASM1161186v1_genomic.fna	93,9955	1839	2188
**GB02.fasta**	GCF_019266045.1_ASM1926604v1_genomic.fna	93,9953	1831	2188
**GB02.fasta**	GCF_019734155.1_ASM1973415v1_genomic.fna	93,9721	1832	2188
**GB02.fasta**	GCF_006384225.1_ASM638422v1_genomic.fna	93,9616	1868	2188
**GB02.fasta**	GCF_017459785.1_ASM1745978v1_genomic.fna	93,9538	1838	2188
**GB02.fasta**	GCF_019266085.1_ASM1926608v1_genomic.fna	93,9230	1859	2188
**GB02.fasta**	GCF_004803645.2_ASM480364v2_genomic.fna	93,9180	1866	2188
**GB02.fasta**	GCF_019834405.1_ASM1983440v1_genomic.fna	93,9063	1877	2188
**GB02.fasta**	GCF_019048925.1_ASM1904892v1_genomic.fna	93,8974	1866	2188
**GB02.fasta**	GCF_016598535.1_ASM1659853v1_genomic.fna	93,5467	1800	2188

**GB02.fasta**	GCF_018615075.1_ASM1861507v1_genomic.fna	93,4568	1811	2188
**GB02.fasta**	GCF_002303875.1_ASM230387v1_genomic.fna	93,4476	1800	2188
**GB02.fasta**	GCF_005938745.2_ASM593874v2_genomic.fna	83,5180	1253	2188
**GB02.fasta**	GCF_002300185.1_ASM230018v1_genomic.fna	83,4372	1221	2188
**GB02.fasta**	GCF_013277795.1_ASM1327779v1_genomic.fna	83,4179	1192	2188
**GB02.fasta**	GCF_003023755.1_ASM302375v1_genomic.fna	83,3648	1216	2188
**GB02.fasta**	GCF_019334125.1_ASM1933412v1_genomic.fna	83,3612	1222	2188
**GB02.fasta**	GCF_003097035.1_ASM309703v1_genomic.fna	83,3440	1216	2188
**GB02.fasta**	GCF_003797085.1_ASM379708v1_genomic.fna	83,2820	1223	2188
**GB02.fasta**	GCF_001894805.1_ASM189480v1_genomic.fna	83,2163	1227	2188
**GB02.fasta**	GCF_007558985.1_ASM755898v1_genomic.fna	79,4180	735	2188
**GB02.fasta**	GCF_017068395.1_ASM1706839v1_genomic.fna	79,4112	744	2188
**GB02.fasta**	GCF_019378875.1_ASM1937887v1_genomic.fna	79,3810	725	2188
**GB02.fasta**	GCF_017875695.1_ASM1787569v1_genomic.fna	79,3366	743	2188
**GB02.fasta**	GCF_003688915.1_ASM368891v1_genomic.fna	79,3022	469	2188
**GB02.fasta**	GCF_017068375.1_ASM1706837v1_genomic.fna	79,2995	740	2188
**GB02.fasta**	GCF_002300155.1_ASM230015v1_genomic.fna	79,2933	735	2188
**GB02.fasta**	GCF_910591545.1_ASM91059154v1_genomic.fna	79,2775	749	2188
**GB02.fasta**	GCF_014256275.1_ASM1425627v1_genomic.fna	79,2738	737	2188
**GB02.fasta**	GCF_014138705.1_ASM1413870v1_genomic.fna	79,2684	750	2188
**GB02.fasta**	GCF_012931745.1_ASM1293174v1_genomic.fna	79,2673	750	2188
**GB02.fasta**	GCF_003385055.1_ASM338505v1_genomic.fna	79,2599	721	2188
**GB02.fasta**	GCF_003626495.1_ASM362649v1_genomic.fna	79,2529	762	2188
**GB02.fasta**	GCF_017916235.1_ASM1791623v1_genomic.fna	79,2408	741	2188
**GB02.fasta**	GCF_014230115.1_ASM1423011v1_genomic.fna	79,2368	740	2188
**GB02.fasta**	GCF_016772255.1_ASM1677225v1_genomic.fna	79,2123	782	2188
**GB02.fasta**	GCF_002300195.1_ASM230019v1_genomic.fna	79,1861	760	2188
**GB02.fasta**	GCF_013348805.1_ASM1334880v1_genomic.fna	79,0809	643	2188
**GB02.fasta**	GCF_003350365.1_ASM335036v1_genomic.fna	79,0178	630	2188
**GB02.fasta**	GCF_008369235.1_ASM836923v1_genomic.fna	79,0005	456	2188
**GB02.fasta**	GCF_021166575.1_ASM2116657v1_genomic.fna	78,9493	609	2188
**GB02.fasta**	GCF_004328705.1_ASM432870v1_genomic.fna	78,9390	549	2188
**GB02.fasta**	GCF_017656075.1_ASM1765607v1_genomic.fna	78,9379	620	2188
**GB02.fasta**	GCF_005484805.1_ASM548480v1_genomic.fna	78,9040	578	2188
**GB02.fasta**	GCF_006704125.1_ASM670412v1_genomic.fna	78,9011	550	2188
**GB02.fasta**	GCF_017833315.1_ASM1783331v1_genomic.fna	78,8598	577	2188
**GB02.fasta**	GCF_003130705.1_ASM313070v1_genomic.fna	78,8415	631	2188
**GB02.fasta**	GCF_009859915.1_ASM985991v1_genomic.fna	78,7964	642	2188
**GB02.fasta**	GCF_003051005.1_ASM305100v1_genomic.fna	78,7676	617	2188
**GB02.fasta**	GCF_014217785.1_ASM1421778v1_genomic.fna	78,7467	506	2188
**GB02.fasta**	GCF_001894865.1_ASM189486v1_genomic.fna	78,7205	572	2188
**GB02.fasta**	GCF_014217765.1_ASM1421776v1_genomic.fna	78,7153	518	2188
**GB02.fasta**	GCF_005049235.1_ASM504923v1_genomic.fna	78,6889	580	2188
**GB02.fasta**	GCF_019095205.1_ASM1909520v1_genomic.fna	78,6650	478	2188
**GB02.fasta**	GCF_003050885.1_ASM305088v1_genomic.fna	78,6383	594	2188

**GB02.fasta**	GCF_002269365.1_ASM226936v1_genomic.fna	78,6185	496	2188
**GB02.fasta**	GCF_003004765.2_ASM300476v2_genomic.fna	78,5911	505	2188
**GB02.fasta**	GCF_900187265.1_52295_G01_genomic.fna	78,5783	501	2188
**GB02.fasta**	GCF_005944105.1_ASM594410v1_genomic.fna	78,5653	501	2188
**GB02.fasta**	GCF_019317345.1_ASM1931734v1_genomic.fna	78,5583	504	2188
**GB02.fasta**	GCF_002259485.1_ASM225948v1_genomic.fna	78,5265	470	2188
**GB02.fasta**	GCF_963517305.1_ERR9968740_bin.19_MetaWRAP_v1.3_MAG_genomic.fna	78,5214	440	2188
**GB02.fasta**	GCF_017068295.1_ASM1706829v1_genomic.fna	78,5209	543	2188
**GB02.fasta**	GCF_002258915.1_ASM225891v1_genomic.fna	78,5160	476	2188
**GB02.fasta**	GCF_001894965.1_ASM189496v1_genomic.fna	78,5073	508	2188
**GB02.fasta**	GCF_006148895.1_ASM614889v1_genomic.fna	78,5041	487	2188
**GB02.fasta**	GCF_003288095.1_ASM328809v1_genomic.fna	78,5037	499	2188
**GB02.fasta**	GCF_001047055.1_ASM104705v1_genomic.fna	78,5027	495	2188
**GB02.fasta**	GCF_016839185.1_ASM1683918v1_genomic.fna	78,5002	543	2188
**GB02.fasta**	GCF_019835185.1_ASM1983518v1_genomic.fna	78,4971	512	2188
**GB02.fasta**	GCF_019834795.1_ASM1983479v1_genomic.fna	78,4958	516	2188
**GB02.fasta**	GCF_019834855.1_ASM1983485v1_genomic.fna	78,4935	522	2188
**GB02.fasta**	GCF_015169995.1_ASM1516999v1_genomic.fna	78,4914	506	2188
**GB02.fasta**	GCF_003336925.1_ASM333692v1_genomic.fna	78,4856	497	2188
**GB02.fasta**	GCF_017068255.1_ASM1706825v1_genomic.fna	78,4838	540	2188
**GB02.fasta**	GCF_014854695.1_ASM1485469v1_genomic.fna	78,4791	516	2188
**GB02.fasta**	GCF_019835205.1_ASM1983520v1_genomic.fna	78,4731	515	2188
**GB02.fasta**	GCF_019834785.1_ASM1983478v1_genomic.fna	78,4727	511	2188
**GB02.fasta**	GCF_019834905.1_ASM1983490v1_genomic.fna	78,4671	512	2188
**GB02.fasta**	GCF_019834875.1_ASM1983487v1_genomic.fna	78,4668	517	2188
**GB02.fasta**	GCF_019835085.1_ASM1983508v1_genomic.fna	78,4584	517	2188
**GB02.fasta**	GCF_900455725.1_50477_H01_genomic.fna	78,4582	472	2188
**GB02.fasta**	GCF_019834825.1_ASM1983482v1_genomic.fna	78,4562	530	2188
**GB02.fasta**	GCF_002259035.1_ASM225903v1_genomic.fna	78,4529	469	2188
**GB02.fasta**	GCF_050920855.1_ASM5092085v1_genomic.fna	78,4519	533	2188
**GB02.fasta**	GCF_009883715.1_ASM988371v1_genomic.fna	78,4477	507	2188
**GB02.fasta**	GCF_009831075.1_ASM983107v1_genomic.fna	78,4440	499	2188
**GB02.fasta**	GCF_015654185.1_ASM1565418v1_genomic.fna	78,4355	558	2188
**GB02.fasta**	GCF_017875715.1_ASM1787571v1_genomic.fna	78,4337	530	2188
**GB02.fasta**	GCF_017832185.1_ASM1783218v1_genomic.fna	78,4289	394	2188
**GB02.fasta**	GCF_002863905.1_ASM286390v1_genomic.fna	78,4274	542	2188
**GB02.fasta**	GCF_016907155.1_ASM1690715v1_genomic.fna	78,4186	425	2188
**GB02.fasta**	GCF_019633605.1_ASM1963360v1_genomic.fna	78,4169	509	2188
**GB02.fasta**	GCF_003586525.1_ASM358652v1_genomic.fna	78,4164	546	2188
**GB02.fasta**	GCF_004563845.1_ASM456384v1_genomic.fna	78,4071	482	2188
**GB02.fasta**	GCF_002741725.1_ASM274172v1_genomic.fna	78,4027	544	2188
**GB02.fasta**	GCF_042720095.1_ASM4272009v1_genomic.fna	78,4015	503	2188
**GB02.fasta**	GCF_012272955.1_ASM1227295v1_genomic.fna	78,3961	557	2188
**GB02.fasta**	GCF_014230025.1_ASM1423002v1_genomic.fna	78,3879	558	2188
**GB02.fasta**	GCF_014905595.1_ASM1490559v2_genomic.fna	78,3869	462	2188

**GB02.fasta**	GCF_003086595.1_ASM308659v1_genomic.fna	78,3864	524	2188
**GB02.fasta**	GCF_006351985.2_ASM635198v2_genomic.fna	78,3823	547	2188
**GB02.fasta**	GCF_012726205.1_ASM1272620v1_genomic.fna	78,3750	491	2188
**GB02.fasta**	GCF_965118195.1_Rhodococcus_VITGK2_genomic.fna	78,3589	503	2188
**GB02.fasta**	GCF_019834925.1_ASM1983492v1_genomic.fna	78,3406	510	2188
**GB02.fasta**	GCF_019834985.1_ASM1983498v1_genomic.fna	78,3312	514	2188
**GB02.fasta**	GCF_019835125.1_ASM1983512v1_genomic.fna	78,3254	502	2188
**GB02.fasta**	GCF_016804345.1_ASM1680434v1_genomic.fna	78,3095	535	2188
**GB02.fasta**	GCF_019835095.1_ASM1983509v1_genomic.fna	78,3001	511	2188
**GB02.fasta**	GCF_004011825.1_ASM401182v1_genomic.fna	78,2917	417	2188
**GB02.fasta**	GCF_004011835.1_ASM401183v1_genomic.fna	78,2853	431	2188
**GB02.fasta**	GCF_004153645.1_ASM415364v1_genomic.fna	78,2670	462	2188
**GB02.fasta**	GCF_012843405.1_ASM1284340v1_genomic.fna	78,2538	408	2188
**GB02.fasta**	GCF_008011915.1_ASM801191v1_genomic.fna	78,2284	446	2188
**GB02.fasta**	GCF_012844225.1_ASM1284422v1_genomic.fna	78,2112	401	2188
**GB02.fasta**	GCF_013602415.1_ASM1360241v2_genomic.fna	78,1371	500	2188
**GB02.fasta**	GCF_003641205.1_ASM364120v1_genomic.fna	78,0956	428	2188
**GB02.fasta**	GCF_004345605.1_ASM434560v1_genomic.fna	78,0431	439	2188
**GB02.fasta**	GCF_950073115.1_SRR3965585_bin.8_MetaWRAP_v1.3_MAG_genomic.fna	78,0344	424	2188
**GB02.fasta**	GCF_950067665.1_SRR3960580_bin.6_MetaWRAP_v1.3_MAG_genomic.fna	78,0340	413	2188
**GB02.fasta**	GCF_950071205.1_SRR3961604_bin.6_MetaWRAP_v1.3_MAG_genomic.fna	78,0301	417	2188
**GB02.fasta**	GCF_950062165.1_SRR3960581_bin.1_MetaWRAP_v1.3_MAG_genomic.fna	78,0207	421	2188
**GB02.fasta**	GCF_950073755.1_SRR3960684_bin.1_MetaWRAP_v1.3_MAG_genomic.fna	78,0007	422	2188
**GB02.fasta**	GCF_950061175.1_SRR3964650_bin.8_MetaWRAP_v1.3_MAG_genomic.fna	77,9959	420	2188
**GB02.fasta**	GCF_012726195.1_ASM1272619v1_genomic.fna	77,9795	423	2188
**GB02.fasta**	GCF_950062875.1_SRR3962771_bin.1_MetaWRAP_v1.3_MAG_genomic.fna	77,9659	420	2188
**GB02.fasta**	GCF_950062835.1_SRR3962772_bin.3_MetaWRAP_v1.3_MAG_genomic.fna	77,9581	417	2188
**GB02.fasta**	GCF_950063225.1_SRR3965809_bin.3_MetaWRAP_v1.3_MAG_genomic.fna	77,9500	418	2188
**GB02.fasta**	GCF_950071545.1_SRR3962508_bin.4_MetaWRAP_v1.3_MAG_genomic.fna	77,9358	418	2188
**GB02.fasta**	GCF_014281965.1_ASM1428196v1_genomic.fna	77,8210	376	2188
**GB02.fasta**	GCF_004795875.1_ASM479587v1_genomic.fna	77,8077	381	2188
**GB02.fasta**	GCF_004795915.1_ASM479591v1_genomic.fna	77,8036	379	2188
**GB02.fasta**	GCF_004006015.1_ASM400601v1_genomic.fna	77,3925	253	2188
**GB02.fasta**	**GCF_033142465.1_ASM3314246v1_genomic.fna** *	**98,6258**	**1929**	**2188**
**GB02.fasta**	GCF_042670865.1_ASM4267086v1_genomic.fna	94,9690	1802	2188
**GB02.fasta**	GCF_042794655.1_ASM4279465v1_genomic.fna	94,7764	1779	2188
**GB02.fasta**	GCF_042728675.1_ASM4272867v1_genomic.fna	94,6973	1788	2188
**GB02.fasta**	GCF_053608585.1_ASM5360858v1_genomic.fna	94,6362	1790	2188
**GB02.fasta**	GCF_040637765.1_ASM4063776v1_genomic.fna	94,3245	1941	2188
**GB02.fasta**	GCF_047468735.1_ASM4746873v1_genomic.fna	94,2329	1891	2188
**GB02.fasta**	GCF_900094765.1_IMG-taxon_2615840625_annotated_assembly_genomic.fna	94,2073	1869	2188
**GB02.fasta**	GCF_937770285.1_FRAM_RAS_EGC1617_bin27_genomic.fna	94,1789	1805	2188
**GB02.fasta**	GCF_036790505.1_ASM3679050v1_genomic.fna	94,1764	1874	2188
**GB02.fasta**	GCF_046271885.1_ASM4627188v1_genomic.fna	94,1714	1889	2188
**GB02.fasta**	GCF_041464245.1_ASM4146424v1_genomic.fna	94,1461	1887	2188

**GB02.fasta**	GCF_036790485.1_ASM3679048v1_genomic.fna	94,1443	1856	2188
**GB02.fasta**	GCF_044908235.1_ASM4490823v1_genomic.fna	94,1374	1881	2188
**GB02.fasta**	GCF_900455855.1_52601_H01_genomic.fna	94,1266	1849	2188
**GB02.fasta**	GCF_051615655.1_ASM5161565v1_genomic.fna	94,1145	1799	2188
**GB02.fasta**	GCF_040608595.1_ASM4060859v1_genomic.fna	94,1070	1802	2188
**GB02.fasta**	GCF_029457415.1_ASM2945741v1_genomic.fna	94,1062	1825	2188
**GB02.fasta**	GCF_036964505.1_ASM3696450v1_genomic.fna	94,1030	1801	2188
**GB02.fasta**	GCF_046097995.1_ASM4609799v1_genomic.fna	94,0994	1843	2188
**GB02.fasta**	GCF_036888805.1_ASM3688880v1_genomic.fna	94,0962	1861	2188
**GB02.fasta**	GCF_030208925.1_ASM3020892v1_genomic.fna	94,0883	1829	2188
**GB02.fasta**	GCF_052906325.1_ASM5290632v1_genomic.fna	94,0848	1825	2188
**GB02.fasta**	GCF_026153295.1_ASM2615329v1_genomic.fna	94,0742	1824	2188
**GB02.fasta**	GCF_032936355.1_ASM3293635v1_genomic.fna	94,0742	1824	2188
**GB02.fasta**	GCF_048575435.1_ASM4857543v1_genomic.fna	94,0736	1823	2188
**GB02.fasta**	GCF_029958325.1_ASM2995832v2_genomic.fna	94,0727	1814	2188
**GB02.fasta**	GCF_030063705.1_ASM3006370v1_genomic.fna	94,0724	1834	2188
**GB02.fasta**	GCF_030057155.1_ASM3005715v1_genomic.fna	94,0719	1813	2188
**GB02.fasta**	GCF_054189245.1_ASM5418924v1_genomic.fna	94,0705	1816	2188
**GB02.fasta**	GCF_037463925.1_ASM3746392v1_genomic.fna	94,0705	1816	2188
**GB02.fasta**	GCF_043079145.1_ASM4307914v1_genomic.fna	94,0678	1866	2188
**GB02.fasta**	GCF_044390185.1_ASM4439018v1_genomic.fna	94,0646	1867	2188
**GB02.fasta**	GCF_044761305.1_ASM4476130v1_genomic.fna	94,0606	1865	2188
**GB02.fasta**	GCF_025962035.1_ASM2596203v1_genomic.fna	94,0605	1831	2188
**GB02.fasta**	GCF_043077445.1_ASM4307744v1_genomic.fna	94,0585	1859	2188
**GB02.fasta**	GCF_052907065.1_ASM5290706v1_genomic.fna	94,0563	1833	2188
**GB02.fasta**	GCF_044390915.1_ASM4439091v1_genomic.fna	94,0552	1872	2188
**GB02.fasta**	GCF_044390285.1_ASM4439028v1_genomic.fna	94,0538	1865	2188
**GB02.fasta**	GCF_044330745.1_ASM4433074v1_genomic.fna	94,0523	1866	2188
**GB02.fasta**	GCF_900101565.1_IMG-taxon_2593339195_annotated_assembly_genomic.fna	94,0511	1790	2188
**GB02.fasta**	GCF_032423855.1_ASM3242385v1_genomic.fna	94,0496	1825	2188
**GB02.fasta**	GCF_038096185.1_ASM3809618v1_genomic.fna	94,0488	1832	2188
**GB02.fasta**	GCF_044390665.1_ASM4439066v1_genomic.fna	94,0482	1862	2188
**GB02.fasta**	GCF_050321945.1_ASM5032194v1_genomic.fna	94,0445	1794	2188
**GB02.fasta**	GCF_042880415.1_ASM4288041v1_genomic.fna	94,0444	1809	2188
**GB02.fasta**	GCF_054130455.1_ASM5413045v1_genomic.fna	94,0430	1792	2188
**GB02.fasta**	GCF_044755865.1_ASM4475586v1_genomic.fna	94,0430	1838	2188
**GB02.fasta**	GCF_044390705.1_ASM4439070v1_genomic.fna	94,0408	1868	2188
**GB02.fasta**	GCF_042883745.1_ASM4288374v1_genomic.fna	94,0386	1795	2188
**GB02.fasta**	GCF_047918755.1_ASM4791875v1_genomic.fna	94,0381	1785	2188
**GB02.fasta**	GCF_044390245.1_ASM4439024v1_genomic.fna	94,0341	1865	2188
**GB02.fasta**	GCF_030063725.1_ASM3006372v1_genomic.fna	94,0311	1839	2188
**GB02.fasta**	GCF_048573875.1_SB1D_genomic.fna	94,0267	1829	2188
**GB02.fasta**	GCF_044390105.1_ASM4439010v1_genomic.fna	94,0228	1871	2188
**GB02.fasta**	GCF_052618535.1_ASM5261853v1_genomic.fna	94,0226	1789	2188
**GB02.fasta**	GCF_040546695.1_ASM4054669v1_genomic.fna	94,0207	1838	2188

**GB02.fasta**	GCF_025246495.1_ASM2524649v1_genomic.fna	94,0196	1831	2188
**GB02.fasta**	GCF_051311045.1_ASM5131104v1_genomic.fna	94,0177	1848	2188
**GB02.fasta**	GCF_041875035.1_ASM4187503v1_genomic.fna	94,0158	1805	2188
**GB02.fasta**	GCF_031763325.1_ASM3176332v1_genomic.fna	94,0094	1822	2188
**GB02.fasta**	GCF_052916325.1_ASM5291632v1_genomic.fna	94,0069	1837	2188
**GB02.fasta**	GCF_042721755.1_ASM4272175v1_genomic.fna	93,9944	1802	2188
**GB02.fasta**	GCF_042266695.1_ASM4226669v1_genomic.fna	93,9904	1812	2188
**GB02.fasta**	GCF_033042395.1_ASM3304239v1_genomic.fna	93,9872	1829	2188
**GB02.fasta**	GCF_044543075.1_ASM4454307v1_genomic.fna	93,9833	1821	2188
**GB02.fasta**	GCF_030056935.1_ASM3005693v1_genomic.fna	93,9827	1778	2188
**GB02.fasta**	GCF_053616015.1_ASM5361601v1_genomic.fna	93,9819	1832	2188
**GB02.fasta**	GCF_033142365.1_ASM3314236v1_genomic.fna	93,9767	1815	2188
**GB02.fasta**	GCF_047468745.1_ASM4746874v1_genomic.fna	93,9670	1815	2188
**GB02.fasta**	GCF_029269915.1_ASM2926991v1_genomic.fna	93,9667	1802	2188
**GB02.fasta**	GCF_036669995.1_ASM3666999v1_genomic.fna	93,9524	1873	2188
**GB02.fasta**	GCF_042882325.1_ASM4288232v1_genomic.fna	93,9503	1808	2188
**GB02.fasta**	GCF_044460645.1_ASM4446064v1_genomic.fna	93,9347	1838	2188
**GB02.fasta**	GCF_042566215.1_ASM4256621v1_genomic.fna	93,9285	1807	2188
**GB02.fasta**	GCF_042795195.1_ASM4279519v1_genomic.fna	93,9247	1812	2188
**GB02.fasta**	GCF_027583855.1_ASM2758385v1_genomic.fna	93,9221	1816	2188
**GB02.fasta**	GCF_054489645.1_ASM5448964v1_genomic.fna	93,9093	1784	2188
**GB02.fasta**	GCF_037104865.1_ASM3710486v1_genomic.fna	93,8812	1808	2188
**GB02.fasta**	GCF_036541985.1_ASM3654198v1_genomic.fna	93,8259	1806	2188
**GB02.fasta**	GCF_030056955.1_ASM3005695v1_genomic.fna	93,8228	1795	2188
**GB02.fasta**	GCF_040489395.1_ASM4048939v1_genomic.fna	93,6102	1839	2188
**GB02.fasta**	GCF_937864515.1_SRR5881626_bin.57_CONCOCT_v1.1_MAG_genomic.fna	93,6066	1832	2188
**GB02.fasta**	GCF_040674095.1_ASM4067409v1_genomic.fna	93,6057	1843	2188
**GB02.fasta**	GCF_040489295.1_ASM4048929v1_genomic.fna	93,5979	1831	2188
**GB02.fasta**	GCF_040489335.1_ASM4048933v1_genomic.fna	93,5959	1830	2188
**GB02.fasta**	GCF_040489235.1_ASM4048923v1_genomic.fna	93,5788	1844	2188
**GB02.fasta**	GCF_040489275.1_ASM4048927v1_genomic.fna	93,5551	1844	2188
**GB02.fasta**	GCF_040489365.1_ASM4048936v1_genomic.fna	93,5522	1845	2188
**GB02.fasta**	GCF_040489215.1_ASM4048921v1_genomic.fna	93,5515	1851	2188
**GB02.fasta**	GCF_040489315.1_ASM4048931v1_genomic.fna	93,5426	1851	2188
**GB02.fasta**	GCF_042881175.1_ASM4288117v1_genomic.fna	93,4842	1808	2188
**GB02.fasta**	GCF_044452125.1_ASM4445212v1_genomic.fna	93,4121	1821	2188
**GB02.fasta**	GCA_039535945.1_ASM3953594v1_genomic.fna	93,3746	1872	2188
**GB02.fasta**	GCF_050586165.1_ASM5058616v1_genomic.fna	83,4100	1210	2188
**GB02.fasta**	GCF_042790355.1_ASM4279035v1_genomic.fna	83,4018	1211	2188
**GB02.fasta**	GCF_047715495.1_AX7B_genomic.fna	83,3749	1223	2188
**GB02.fasta**	GCF_033142425.1_ASM3314242v1_genomic.fna	83,2847	1236	2188
**GB02.fasta**	GCF_004211705.1_ASM421170v1_genomic.fna	83,1382	1224	2188
**GB02.fasta**	GCF_049739425.1_ASM4973942v1_genomic.fna	81,6958	931	2188
**GB02.fasta**	GCF_038447315.2_DK176_genomic.fna	79,2762	747	2188
**GB02.fasta**	GCF_900105905.1_IMG-taxon_2634166362_annotated_assembly_genomic.fna	79,2723	745	2188

**GB02.fasta**	GCF_900455735.1_55312_C01_genomic.fna	79,2703	717	2188
**GB02.fasta**	GCF_048288345.1_ASM4828834v1_genomic.fna	79,2629	756	2188
**GB02.fasta**	GCF_030656675.1_ASM3065667v1_genomic.fna	79,2606	767	2188
**GB02.fasta**	GCF_038447005.2_DK180_genomic.fna	79,2545	751	2188
**GB02.fasta**	GCF_033842605.1_ASM3384260v1_genomic.fna	79,2337	761	2188
**GB02.fasta**	GCF_040961855.1_ASM4096185v1_genomic.fna	79,2332	772	2188
**GB02.fasta**	GCF_038447505.2_DK17_genomic.fna	79,2232	751	2188
**GB02.fasta**	GCF_033042225.1_ASM3304222v1_genomic.fna	79,2067	460	2188
**GB02.fasta**	GCF_040702585.1_ASM4070258v1_genomic.fna	79,2061	453	2188
**GB02.fasta**	GCF_030058635.1_ASM3005863v1_genomic.fna	79,2052	733	2188
**GB02.fasta**	GCF_041228765.1_ASM4122876v1_genomic.fna	79,2015	754	2188
**GB02.fasta**	GCF_900105375.1_IMG-taxon_2642422554_annotated_assembly_genomic.fna	79,1682	735	2188
**GB02.fasta**	GCF_029893305.1_ASM2989330v1_genomic.fna	79,1564	758	2188
**GB02.fasta**	GCF_042682305.1_ASM4268230v1_genomic.fna	79,1385	742	2188
**GB02.fasta**	GCF_033142395.1_ASM3314239v1_genomic.fna	79,1048	450	2188
**GB02.fasta**	GCF_033142385.1_ASM3314238v1_genomic.fna	79,0866	465	2188
**GB02.fasta**	GCF_050320375.1_KRD162_genomic.fna	79,0496	415	2188
**GB02.fasta**	GCF_040702765.1_ASM4070276v1_genomic.fna	79,0388	487	2188
**GB02.fasta**	GCF_042720775.1_ASM4272077v1_genomic.fna	78,9863	529	2188
**GB02.fasta**	GCF_046042785.1_ASM4604278v1_genomic.fna	78,9846	628	2188
**GB02.fasta**	GCF_042754885.1_ASM4275488v1_genomic.fna	78,9790	533	2188
**GB02.fasta**	GCF_050433545.1_ASM5043354v1_genomic.fna	78,9628	654	2188
**GB02.fasta**	GCF_044542515.1_ASM4454251v1_genomic.fna	78,9615	427	2188
**GB02.fasta**	GCF_030056895.1_ASM3005689v1_genomic.fna	78,9570	427	2188
**GB02.fasta**	GCF_055231055.1_ASM5523105v1_genomic.fna	78,9489	501	2188
**GB02.fasta**	GCF_042720435.1_ASM4272043v1_genomic.fna	78,9377	546	2188
**GB02.fasta**	GCF_040686915.1_ASM4068691v1_genomic.fna	78,9138	464	2188
**GB02.fasta**	GCF_042718675.1_ASM4271867v1_genomic.fna	78,9056	537	2188
**GB02.fasta**	GCF_042654025.1_ASM4265402v1_genomic.fna	78,8992	577	2188
**GB02.fasta**	GCF_900101735.1_IMG-taxon_2675903015_annotated_assembly_genomic.fna	78,8966	606	2188
**GB02.fasta**	GCF_033042195.1_ASM3304219v1_genomic.fna	78,8805	468	2188
**GB02.fasta**	GCF_050477185.1_ASM5047718v1_genomic.fna	78,8709	654	2188
**GB02.fasta**	GCF_055231015.1_ASM5523101v1_genomic.fna	78,8542	425	2188
**GB02.fasta**	GCF_033101215.1_ASM3310121v1_genomic.fna	78,8317	589	2188
**GB02.fasta**	GCF_040702225.1_ASM4070222v1_genomic.fna	78,8293	468	2188
**GB02.fasta**	GCF_042719695.1_ASM4271969v1_genomic.fna	78,8289	476	2188
**GB02.fasta**	GCF_043197445.1_ASM4319744v1_genomic.fna	78,7838	591	2188
**GB02.fasta**	GCF_044389245.1_ASM4438924v1_genomic.fna	78,7820	621	2188
**GB02.fasta**	GCF_044389455.1_ASM4438945v1_genomic.fna	78,7795	622	2188
**GB02.fasta**	GCF_044389235.1_ASM4438923v1_genomic.fna	78,7757	620	2188
**GB02.fasta**	GCF_042665615.1_ASM4266561v1_genomic.fna	78,7619	575	2188
**GB02.fasta**	GCF_033101395.1_ASM3310139v1_genomic.fna	78,7478	584	2188
**GB02.fasta**	GCF_044389185.1_ASM4438918v1_genomic.fna	78,7281	621	2188
**GB02.fasta**	GCF_033142305.1_ASM3314230v1_genomic.fna	78,7243	606	2188
**GB02.fasta**	GCF_033101195.1_ASM3310119v1_genomic.fna	78,7228	592	2188

**GB02.fasta**	GCF_055230895.1_ASM5523089v1_genomic.fna	78,7226	431	2188
**GB02.fasta**	GCF_900109405.1_IMG-taxon_2675903023_annotated_assembly_genomic.fna	78,7219	578	2188
**GB02.fasta**	GCF_033101255.1_ASM3310125v1_genomic.fna	78,7217	600	2188
**GB02.fasta**	GCF_033142325.1_ASM3314232v1_genomic.fna	78,7200	444	2188
**GB02.fasta**	GCF_033101295.1_ASM3310129v1_genomic.fna	78,7169	606	2188
**GB02.fasta**	GCF_035918275.1_ASM3591827v1_genomic.fna	78,6840	503	2188
**GB02.fasta**	GCF_044457085.1_ASM4445708v1_genomic.fna	78,6630	597	2188
**GB02.fasta**	GCF_040548995.1_ASM4054899v1_genomic.fna	78,6619	595	2188
**GB02.fasta**	GCF_900215125.1_IMG-taxon_2737472030_annotated_assembly_genomic.fna	78,6581	594	2188
**GB02.fasta**	GCF_042672205.1_ASM4267220v1_genomic.fna	78,6547	573	2188
**GB02.fasta**	GCF_042672245.1_ASM4267224v1_genomic.fna	78,6487	589	2188
**GB02.fasta**	GCF_053604015.1_ASM5360401v1_genomic.fna	78,6380	562	2188
**GB02.fasta**	GCF_053589555.1_ASM5358955v1_genomic.fna	78,6241	567	2188
**GB02.fasta**	GCF_053589375.1_ASM5358937v1_genomic.fna	78,6228	549	2188
**GB02.fasta**	GCF_050907905.1_ASM5090790v1_genomic.fna	78,6177	504	2188
**GB02.fasta**	GCF_041393875.1_ASM4139387v1_genomic.fna	78,6165	493	2188
**GB02.fasta**	GCF_048319925.1_ASM4831992v1_genomic.fna	78,6138	479	2188
**GB02.fasta**	GCF_016598695.1_ASM1659869v1_genomic.fna	78,6061	498	2188
**GB02.fasta**	GCF_040256695.1_ASM4025669v1_genomic.fna	78,6023	500	2188
**GB02.fasta**	GCF_053604605.1_ASM5360460v1_genomic.fna	78,5886	499	2188
**GB02.fasta**	GCF_900099725.1_IMG-taxon_2675903013_annotated_assembly_genomic.fna	78,5865	511	2188
**GB02.fasta**	GCF_050321925.1_ASM5032192v1_genomic.fna	78,5803	480	2188
**GB02.fasta**	GCF_033101435.1_ASM3310143v1_genomic.fna	78,5789	480	2188
**GB02.fasta**	GCF_042873335.1_ASM4287333v1_genomic.fna	78,5707	494	2188
**GB02.fasta**	GCF_053604585.1_ASM5360458v1_genomic.fna	78,5680	484	2188
**GB02.fasta**	GCF_900105195.1_IMG-taxon_2630969012_annotated_assembly_genomic.fna	78,5664	489	2188
**GB02.fasta**	GCF_019835465.1_ASM1983546v1_genomic.fna	78,5650	510	2188
**GB02.fasta**	GCF_039565925.1_ASM3956592v1_genomic.fna	78,5566	547	2188
**GB02.fasta**	GCF_053555925.1_ASM5355592v1_genomic.fna	78,5489	502	2188
**GB02.fasta**	GCF_050321885.1_ASM5032188v1_genomic.fna	78,5434	540	2188
**GB02.fasta**	GCF_050321985.1_ASM5032198v1_genomic.fna	78,5185	493	2188
**GB02.fasta**	GCF_042888185.1_ASM4288818v1_genomic.fna	78,5173	497	2188
**GB02.fasta**	GCF_019835485.1_ASM1983548v1_genomic.fna	78,5161	513	2188
**GB02.fasta**	GCF_050321905.1_ASM5032190v1_genomic.fna	78,5148	547	2188
**GB02.fasta**	GCF_053588355.1_ASM5358835v1_genomic.fna	78,5083	504	2188
**GB02.fasta**	GCF_053588375.1_ASM5358837v1_genomic.fna	78,5050	502	2188
**GB02.fasta**	GCF_045488035.1_ASM4548803v1_genomic.fna	78,5044	536	2188
**GB02.fasta**	GCF_053604465.1_ASM5360446v1_genomic.fna	78,5016	488	2188
**GB02.fasta**	GCF_053588735.1_ASM5358873v1_genomic.fna	78,4986	492	2188
**GB02.fasta**	GCF_030979975.1_ASM3097997v1_genomic.fna	78,4956	493	2188
**GB02.fasta**	GCF_019835255.1_ASM1983525v1_genomic.fna	78,4838	521	2188
**GB02.fasta**	GCF_019835235.1_ASM1983523v1_genomic.fna	78,4837	518	2188
**GB02.fasta**	GCF_042681175.1_ASM4268117v1_genomic.fna	78,4829	550	2188
**GB02.fasta**	GCF_045516015.1_ASM4551601v1_genomic.fna	78,4770	557	2188
**GB02.fasta**	GCF_053764755.1_ASM5376475v1_genomic.fna	78,4739	537	2188

**GB02.fasta**	GCF_042729375.1_ASM4272937v1_genomic.fna	78,4703	504	2188
**GB02.fasta**	GCF_030506605.1_ASM3050660v1_genomic.fna	78,4703	502	2188
**GB02.fasta**	GCF_019835285.1_ASM1983528v1_genomic.fna	78,4692	517	2188
**GB02.fasta**	GCF_019834995.1_ASM1983499v1_genomic.fna	78,4685	520	2188
**GB02.fasta**	GCF_019835425.1_ASM1983542v1_genomic.fna	78,4676	516	2188
**GB02.fasta**	GCF_029457455.1_ASM2945745v1_genomic.fna	78,4673	518	2188
**GB02.fasta**	GCF_049538275.1_ASM4953827v1_genomic.fna	78,4659	454	2188
**GB02.fasta**	GCF_037327425.1_ASM3732742v1_genomic.fna	78,4656	432	2188
**GB02.fasta**	GCF_019835385.1_ASM1983538v1_genomic.fna	78,4656	516	2188
**GB02.fasta**	GCF_033142445.1_ASM3314244v1_genomic.fna	78,4651	443	2188
**GB02.fasta**	GCF_030057105.1_ASM3005710v1_genomic.fna	78,4651	494	2188
**GB02.fasta**	GCF_040636045.1_ASM4063604v1_genomic.fna	78,4646	479	2188
**GB02.fasta**	GCF_900478115.1_50618_D02_genomic.fna	78,4592	423	2188
**GB02.fasta**	GCF_030818285.1_ASM3081828v1_genomic.fna	78,4552	402	2188
**GB02.fasta**	GCF_044227165.1_ASM4422716v1_genomic.fna	78,4521	541	2188
**GB02.fasta**	GCF_019835365.1_ASM1983536v1_genomic.fna	78,4511	521	2188
**GB02.fasta**	GCF_054960675.1_ASM5496067v1_genomic.fna	78,4493	431	2188
**GB02.fasta**	GCF_036363425.1_ASM3636342v1_genomic.fna	78,4490	559	2188
**GB02.fasta**	GCF_019835445.1_ASM1983544v1_genomic.fna	78,4443	520	2188
**GB02.fasta**	GCF_054949225.1_ASM5494922v1_genomic.fna	78,4424	551	2188
**GB02.fasta**	GCF_019835215.1_ASM1983521v1_genomic.fna	78,4421	516	2188
**GB02.fasta**	GCF_019834845.1_ASM1983484v1_genomic.fna	78,4417	518	2188
**GB02.fasta**	GCF_019835305.1_ASM1983530v1_genomic.fna	78,4410	517	2188
**GB02.fasta**	GCF_036173065.1_ASM3617306v1_genomic.fna	78,4402	399	2188
**GB02.fasta**	GCF_019835525.1_ASM1983552v1_genomic.fna	78,4399	519	2188
**GB02.fasta**	GCF_054353335.1_ASM5435333v1_genomic.fna	78,4384	543	2188
**GB02.fasta**	GCF_030057035.1_ASM3005703v1_genomic.fna	78,4346	544	2188
**GB02.fasta**	GCF_030819335.1_ASM3081933v1_genomic.fna	78,4334	397	2188
**GB02.fasta**	GCF_019835325.1_ASM1983532v1_genomic.fna	78,4332	520	2188
**GB02.fasta**	GCF_036163995.1_ASM3616399v1_genomic.fna	78,4332	407	2188
**GB02.fasta**	GCF_036363445.1_ASM3636344v1_genomic.fna	78,4312	562	2188
**GB02.fasta**	GCF_050321865.1_ASM5032186v1_genomic.fna	78,4307	429	2188
**GB02.fasta**	GCF_019835545.1_ASM1983554v1_genomic.fna	78,4255	524	2188
**GB02.fasta**	GCF_019835405.1_ASM1983540v1_genomic.fna	78,4245	522	2188
**GB02.fasta**	GCF_040656415.1_ASM4065641v1_genomic.fna	78,4242	403	2188
**GB02.fasta**	GCF_019835335.1_ASM1983533v1_genomic.fna	78,4156	517	2188
**GB02.fasta**	GCF_053604485.1_ASM5360448v1_genomic.fna	78,4094	549	2188
**GB02.fasta**	GCF_051172915.1_ASM5117291v1_genomic.fna	78,4084	567	2188
**GB02.fasta**	GCF_977109115.1_m2n_genomic.fna	78,4074	467	2188
**GB02.fasta**	GCF_030550835.1_ASM3055083v1_genomic.fna	78,4060	557	2188
**GB02.fasta**	GCF_053602315.1_ASM5360231v1_genomic.fna	78,4055	529	2188
**GB02.fasta**	GCF_900177695.1_IMG-taxon_2527291702_annotated_assembly_genomic.fna	78,4050	510	2188
**GB02.fasta**	GCF_053764735.1_ASM5376473v1_genomic.fna	78,4035	505	2188
**GB02.fasta**	GCF_030490385.1_ASM3049038v1_genomic.fna	78,3974	550	2188
**GB02.fasta**	GCF_047782425.1_ASM4778242v1_genomic.fna	78,3899	448	2188

**GB02.fasta**	GCF_036363005.1_ASM3636300v1_genomic.fna	78,3891	563	2188
**GB02.fasta**	GCF_042719255.1_ASM4271925v1_genomic.fna	78,3871	508	2188
**GB02.fasta**	GCF_038853135.1_ASM3885313v1_genomic.fna	78,3850	527	2188
**GB02.fasta**	GCF_021441945.1_ASM2144194v1_genomic.fna	78,3778	526	2188
**GB02.fasta**	GCF_042879035.1_ASM4287903v1_genomic.fna	78,3767	571	2188
**GB02.fasta**	GCF_053588715.1_ASM5358871v1_genomic.fna	78,3749	538	2188
**GB02.fasta**	GCF_019835725.1_ASM1983572v1_genomic.fna	78,3746	391	2188
**GB02.fasta**	GCF_027270735.1_ASM2727073v1_genomic.fna	78,3730	436	2188
**GB02.fasta**	GCF_019835985.1_ASM1983598v1_genomic.fna	78,3597	387	2188
**GB02.fasta**	GCF_030916465.1_ASM3091646v1_genomic.fna	78,3597	537	2188
**GB02.fasta**	GCF_019835665.1_ASM1983566v1_genomic.fna	78,3583	391	2188
**GB02.fasta**	GCF_042873755.1_ASM4287375v1_genomic.fna	78,3575	549	2188
**GB02.fasta**	GCF_040655995.1_ASM4065599v1_genomic.fna	78,3539	417	2188
**GB02.fasta**	GCF_036364245.1_ASM3636424v1_genomic.fna	78,3504	559	2188
**GB02.fasta**	GCF_053589115.1_ASM5358911v1_genomic.fna	78,3500	547	2188
**GB02.fasta**	GCF_050848365.1_ASM5084836v1_genomic.fna	78,3401	525	2188
**GB02.fasta**	GCF_033042295.1_ASM3304229v1_genomic.fna	78,3374	558	2188
**GB02.fasta**	GCF_053600935.1_ASM5360093v1_genomic.fna	78,3357	534	2188
**GB02.fasta**	GCF_019835805.1_ASM1983580v1_genomic.fna	78,3342	396	2188
**GB02.fasta**	GCF_042750145.1_ASM4275014v1_genomic.fna	78,3290	558	2188
**GB02.fasta**	GCF_019835855.1_ASM1983585v1_genomic.fna	78,3209	389	2188
**GB02.fasta**	GCF_019835945.1_ASM1983594v1_genomic.fna	78,3209	389	2188
**GB02.fasta**	GCF_019835495.1_ASM1983549v1_genomic.fna	78,3209	389	2188
**GB02.fasta**	GCF_030208955.1_ASM3020895v1_genomic.fna	78,3192	385	2188
**GB02.fasta**	GCF_040678935.1_ASM4067893v1_genomic.fna	78,3183	418	2188
**GB02.fasta**	GCF_019835645.1_ASM1983564v1_genomic.fna	78,3181	396	2188
**GB02.fasta**	GCF_019836175.1_ASM1983617v1_genomic.fna	78,3174	406	2188
**GB02.fasta**	GCF_042729855.1_ASM4272985v1_genomic.fna	78,3164	455	2188
**GB02.fasta**	GCF_019835765.1_ASM1983576v1_genomic.fna	78,3156	392	2188
**GB02.fasta**	GCF_019835555.1_ASM1983555v1_genomic.fna	78,3119	390	2188
**GB02.fasta**	GCF_047782815.1_Unicycler_genomic.fna	78,3114	529	2188
**GB02.fasta**	GCF_031456475.1_ASM3145647v1_genomic.fna	78,3098	452	2188
**GB02.fasta**	GCF_019835925.1_ASM1983592v1_genomic.fna	78,3066	393	2188
**GB02.fasta**	GCF_019835825.1_ASM1983582v1_genomic.fna	78,3066	393	2188
**GB02.fasta**	GCF_041365675.1_ASM4136567v1_genomic.fna	78,3004	473	2188
**GB02.fasta**	GCF_019835615.1_ASM1983561v1_genomic.fna	78,2943	392	2188
**GB02.fasta**	GCF_053602415.1_ASM5360241v1_genomic.fna	78,2921	548	2188
**GB02.fasta**	GCF_019835995.1_ASM1983599v1_genomic.fna	78,2891	394	2188
**GB02.fasta**	GCF_019835685.1_ASM1983568v1_genomic.fna	78,2891	394	2188
**GB02.fasta**	GCF_019835585.1_ASM1983558v1_genomic.fna	78,2891	394	2188
**GB02.fasta**	GCF_019835965.1_ASM1983596v1_genomic.fna	78,2891	394	2188
**GB02.fasta**	GCF_044459205.1_ASM4445920v1_genomic.fna	78,2883	419	2188
**GB02.fasta**	GCF_019835905.1_ASM1983590v1_genomic.fna	78,2841	397	2188
**GB02.fasta**	GCF_019835745.1_ASM1983574v1_genomic.fna	78,2841	397	2188
**GB02.fasta**	GCF_048957735.1_ASM4895773v1_genomic.fna	78,2814	474	2188

**GB02.fasta**	GCF_030505055.1_ASM3050505v1_genomic.fna	78,2752	533	2188
**GB02.fasta**	GCF_019835785.1_ASM1983578v1_genomic.fna	78,2744	393	2188
**GB02.fasta**	GCF_044457185.1_ASM4445718v1_genomic.fna	78,2737	431	2188
**GB02.fasta**	GCF_054889305.1_ASM5488930v1_genomic.fna	78,2683	538	2188
**GB02.fasta**	GCF_019835885.1_ASM1983588v1_genomic.fna	78,2672	398	2188
**GB02.fasta**	GCF_019835605.1_ASM1983560v1_genomic.fna	78,2672	398	2188
**GB02.fasta**	GCF_019835845.1_ASM1983584v1_genomic.fna	78,2672	398	2188
**GB02.fasta**	GCF_042717545.1_ASM4271754v1_genomic.fna	78,1787	424	2188
**GB02.fasta**	GCF_965342405.1_BGI_Ryan_01_genomic.fna	78,1675	417	2188
**GB02.fasta**	GCF_050320385.1_ASM5032038v1_genomic.fna	78,1623	437	2188
**GB02.fasta**	GCF_049844875.1_ASM4984487v1_genomic.fna	78,1578	412	2188
**GB02.fasta**	GCF_042730335.1_ASM4273033v1_genomic.fna	78,1109	395	2188
**GB02.fasta**	GCF_030056795.1_ASM3005679v1_genomic.fna	78,0908	417	2188
**GB02.fasta**	GCF_042667435.1_ASM4266743v1_genomic.fna	78,0752	434	2188
**GB02.fasta**	GCF_051027685.1_Rhodococcus_sp._BGS-1C_genomic.fna	78,0666	428	2188
**GB02.fasta**	GCF_054979605.1_ASM5497960v1_genomic.fna	78,0504	306	2188
**GB02.fasta**	GCF_051918125.1_ASM5191812v1_genomic.fna	78,0319	439	2188
**GB02.fasta**	GCF_040643435.1_ASM4064343v1_genomic.fna	78,0224	424	2188
**GB02.fasta**	GCF_053750035.1_ASM5375003v1_genomic.fna	78,0023	445	2188
**GB02.fasta**	GCF_055193735.1_ASM5519373v1_genomic.fna	77,9923	399	2188
**GB02.fasta**	GCF_913060425.1_SRR3965585_bin.11_MetaBAT_v2.12.1_MAG_genomic.fna	77,9916	420	2188
**GB02.fasta**	GCF_023015605.1_ASM2301560v1_genomic.fna	77,9867	432	2188
**GB02.fasta**	GCF_950061265.1_SRR3963456_bin.3_MetaWRAP_v1.3_MAG_genomic.fna	77,9764	418	2188
**GB02.fasta**	GCF_950062745.1_SRR3961936_bin.1_MetaWRAP_v1.3_MAG_genomic.fna	77,9603	416	2188
**GB02.fasta**	GCF_030063405.1_ASM3006340v1_genomic.fna	77,9571	366	2188
**GB02.fasta**	GCF_950063375.1_SRR3962777_bin.2_MetaWRAP_v1.3_MAG_genomic.fna	77,9032	420	2188
**GB02.fasta**	GCF_055230935.1_ASM5523093v1_genomic.fna	77,8862	399	2188
**GB02.fasta**	GCF_036327795.1_ASM3632779v1_genomic.fna	77,8643	476	2188
**GB02.fasta**	GCF_040547235.1_ASM4054723v1_genomic.fna	77,8339	371	2188
**GB02.fasta**	GCF_040547225.1_ASM4054722v1_genomic.fna	77,7957	368	2188
GB02.fasta	**GCF_027270535.1_ASM2727053v1_genomic.fna ****	**98,1124**	**1911**	**2188**
**GB02.fasta**	GCF_030056735.1_ASM3005673v1_genomic.fna	94,5243	1743	2188
**GB02.fasta**	GCF_027270815.1_ASM2727081v1_genomic.fna	94,2910	1888	2188
**GB02.fasta**	GCF_027270845.1_ASM2727084v1_genomic.fna	94,2207	1869	2188
**GB02.fasta**	GCF_024438075.1_ASM2443807v1_genomic.fna	94,2038	1880	2188
**GB02.fasta**	GCF_027854095.1_ASM2785409v1_genomic.fna	94,1655	1883	2188
**GB02.fasta**	GCF_024436645.1_ASM2443664v1_genomic.fna	94,1359	1869	2188
**GB02.fasta**	GCF_030056745.1_ASM3005674v1_genomic.fna	94,1347	1840	2188
**GB02.fasta**	GCF_021352185.1_ASM2135218v1_genomic.fna	94,1012	1809	2188
**GB02.fasta**	GCF_025370975.1_ASM2537097v1_genomic.fna	94,0936	1816	2188
**GB02.fasta**	GCF_025961405.1_ASM2596140v1_genomic.fna	94,0891	1822	2188
**GB02.fasta**	GCF_024809795.1_ASM2480979v1_genomic.fna	94,0695	1813	2188
**GB02.fasta**	GCF_020809065.1_ASM2080906v1_genomic.fna	94,0675	1794	2188
**GB02.fasta**	GCF_030063525.1_ASM3006352v1_genomic.fna	94,0547	1782	2188
**GB02.fasta**	GCF_021457505.1_ASM2145750v1_genomic.fna	94,0517	1832	2188

**GB02.fasta**	GCF_022134675.1_ASM2213467v1_genomic.fna	94,0438	1808	2188
**GB02.fasta**	GCF_020782825.1_ASM2078282v1_genomic.fna	94,0339	1851	2188
**GB02.fasta**	GCF_026074015.1_ASM2607401v1_genomic.fna	94,0294	1889	2188
**GB02.fasta**	GCF_024438095.1_ASM2443809v1_genomic.fna	94,0271	1805	2188
**GB02.fasta**	GCF_024498635.1_ASM2449863v1_genomic.fna	94,0216	1854	2188
**GB02.fasta**	GCF_020535445.1_ASM2053544v1_genomic.fna	94,0141	1800	2188
**GB02.fasta**	GCF_022059865.1_ASM2205986v1_genomic.fna	94,0129	1849	2188
**GB02.fasta**	GCF_024349805.1_ASM2434980v1_genomic.fna	93,9525	1824	2188
**GB02.fasta**	GCF_023221595.1_ASM2322159v1_genomic.fna	93,9479	1883	2188
**GB02.fasta**	GCF_021165975.1_ASM2116597v1_genomic.fna	93,9394	1876	2188
**GB02.fasta**	GCF_021026075.1_ASM2102607v1_genomic.fna	93,9303	1817	2188
**GB02.fasta**	GCF_029076895.1_ASM2907689v1_genomic.fna	93,9154	1845	2188
**GB02.fasta**	GCF_030056975.1_ASM3005697v1_genomic.fna	93,8145	1793	2188
**GB02.fasta**	GCF_021497645.1_ASM2149764v1_genomic.fna	93,6183	1820	2188
**GB02.fasta**	GCF_026062495.1_ASM2606249v1_genomic.fna	79,3162	767	2188
**GB02.fasta**	GCF_026810085.1_ASM2681008v1_genomic.fna	79,3068	728	2188
**GB02.fasta**	GCF_022985635.2_ASM2298563v2_genomic.fna	79,2903	761	2188
**GB02.fasta**	GCF_019856255.1_ASM1985625v1_genomic.fna	79,2733	752	2188
**GB02.fasta**	GCF_020542785.2_ASM2054278v2_genomic.fna	79,2020	763	2188
**GB02.fasta**	GCF_022655485.1_ASM2265548v1_genomic.fna	79,1998	759	2188
**GB02.fasta**	GCF_023667485.2_MSC16_LR_genomic.fna	79,1395	618	2188
**GB02.fasta**	GCF_022810405.1_ASM2281040v1_genomic.fna	79,0107	453	2188
**GB02.fasta**	GCF_024438035.1_ASM2443803v1_genomic.fna	78,8217	611	2188
**GB02.fasta**	GCF_027270595.1_ASM2727059v1_genomic.fna	78,8010	580	2188
**GB02.fasta**	GCF_020885655.1_ASM2088565v1_genomic.fna	78,6498	513	2188
**GB02.fasta**	GCF_021165995.1_ASM2116599v1_genomic.fna	78,6390	495	2188
**GB02.fasta**	GCF_026802115.1_ASM2680211v1_genomic.fna	78,6094	506	2188
**GB02.fasta**	GCF_023100785.1_ASM2310078v1_genomic.fna	78,5731	502	2188
**GB02.fasta**	GCF_005503035.1_ASM550303v1_genomic.fna	78,5623	540	2188
**GB02.fasta**	GCF_021026105.1_ASM2102610v1_genomic.fna	78,5522	477	2188
**GB02.fasta**	GCF_024760325.1_ASM2476032v1_genomic.fna	78,5388	507	2188
**GB02.fasta**	GCF_025722975.1_ASM2572297v1_genomic.fna	78,5238	554	2188
**GB02.fasta**	GCF_024321955.1_ASM2432195v1_genomic.fna	78,5206	496	2188
**GB02.fasta**	GCF_021852985.1_ASM2185298v1_genomic.fna	78,4997	544	2188
**GB02.fasta**	GCF_023278385.1_ASM2327838v1_genomic.fna	78,4995	540	2188
**GB02.fasta**	GCF_021128935.1_ASM2112893v1_genomic.fna	78,4927	486	2188
**GB02.fasta**	GCF_023822085.1_ASM2382208v1_genomic.fna	78,4640	484	2188
**GB02.fasta**	GCF_024723275.1_ASM2472327v1_genomic.fna	78,4609	502	2188
**GB02.fasta**	GCF_021165915.1_ASM2116591v1_genomic.fna	78,4545	551	2188
**GB02.fasta**	GCF_020637575.2_ASM2063757v2_genomic.fna	78,4423	524	2188
**GB02.fasta**	GCF_023238485.1_ASM2323848v1_genomic.fna	78,4311	502	2188
**GB02.fasta**	GCF_023704055.1_ASM2370405v1_genomic.fna	78,4200	549	2188
**GB02.fasta**	GCF_040149455.1_ASM4014945v1_genomic.fna	78,4070	513	2188
**GB02.fasta**	GCF_029691645.1_ASM2969164v1_genomic.fna	78,4013	567	2188
**GB02.fasta**	GCF_019836225.1_ASM1983622v1_genomic.fna	78,3942	398	2188

**GB02.fasta**	GCF_019836065.1_ASM1983606v1_genomic.fna	78,3802	395	2188
**GB02.fasta**	GCF_024266895.1_ASM2426689v1_genomic.fna	78,3772	509	2188
**GB02.fasta**	GCF_019835705.1_ASM1983570v1_genomic.fna	78,3746	391	2188
**GB02.fasta**	GCF_019836165.1_ASM1983616v1_genomic.fna	78,3638	401	2188
**GB02.fasta**	GCF_025837095.1_ASM2583709v1_genomic.fna	78,3626	485	2188
**GB02.fasta**	GCF_019836315.1_ASM1983631v1_genomic.fna	78,3464	405	2188
**GB02.fasta**	GCF_019836045.1_ASM1983604v1_genomic.fna	78,3427	391	2188
**GB02.fasta**	GCF_019836085.1_ASM1983608v1_genomic.fna	78,3406	402	2188
**GB02.fasta**	GCF_019836265.1_ASM1983626v1_genomic.fna	78,3388	398	2188
**GB02.fasta**	GCF_024362245.1_ASM2436224v1_genomic.fna	78,3357	472	2188
**GB02.fasta**	GCF_019836025.1_ASM1983602v1_genomic.fna	78,3234	393	2188
**GB02.fasta**	GCF_019836245.1_ASM1983624v1_genomic.fna	78,3217	398	2188
**GB02.fasta**	GCF_019836125.1_ASM1983612v1_genomic.fna	78,3145	401	2188
**GB02.fasta**	GCF_019836105.1_ASM1983610v1_genomic.fna	78,3125	395	2188
**GB02.fasta**	GCF_019836205.1_ASM1983620v1_genomic.fna	78,2991	403	2188
**GB02.fasta**	GCF_019836145.1_ASM1983614v1_genomic.fna	78,1987	402	2188
**GB02.fasta**	GCF_022554085.1_ASM2255408v1_genomic.fna	78,1136	428	2188
**GB02.fasta**	GCF_020889545.1_ASM2088954v1_genomic.fna	78,0387	420	2188
**GB02.fasta**	GCF_027158005.1_ASM2715800v1_genomic.fna	77,6967	360	2188

Note: Genomes highlighted in yellow exhibit ANI values above the 95–96% species-level threshold, indicating the closest genomic relatives of *Rhodococcus* sp. GB-02. * *Rhodococcus* sp. IEGM 1318 (NCBI RefSeq assembly: GCF_033142465.1; Genome assembly: ASM3314246v1), isolated from bottom sediments of a stream in Mys Sakhalin, Novaya Zemlya, Arkhangelsk Region, Russia.** *Rhodococcus erythropolis* IEGM 1415 (NCBI RefSeq assembly: GCF_027270535.1; Genome assembly: ASM2727053v1), isolated from oil-polluted soil in Norilsk, Krasnoyarsk Region, Russia.

Table S2. Predicted metabolic modules identified in the genome of *Rhodococcus* sp. GB-02, including module completeness, detected and missing KEGG Orthologs (KOs), and associated open reading frames (ORFs).

ID	Metabolic pathway	Module	Completeness (%)	Category	Detected KOs	Missing KOs	Total ORFs
**3885**	N-glycosylation by oligosaccharyltransferase	M00072	100	Complete	6	0	69
**3889**	Dermatan sulfate degradation	M00076	80	Partial	5	1	35
**3890**	Chondroitin sulfate degradation	M00077	75	Partial	4	1	32
**3891**	Keratan sulfate degradation	M00079	75	Partial	4	1	22
**3892**	NADH:ubiquinone oxidoreductase, mitochondria	M00142	100	Complete	7	0	112
**3893**	NADH dehydrogenase (ubiquinone) Fe-S protein/flavoprotein complex, mitochondria	M00143	100	Complete	10	0	252
**3894**	NADH:quinone oxidoreductase, prokaryotes	M00144	100	Complete	16	0	543
**3895**	NAD(P)H:quinone oxidoreductase, chloroplasts and cyanobacteria	M00145	100	Complete	11	0	221
**3898**	Succinate dehydrogenase (ubiquinone)	M00148	100	Complete	5	0	63
**3899**	Succinate dehydrogenase, prokaryotes	M00149	100	Complete	6	0	127
**3900**	Fumarate reductase, prokaryotes	M00150	100	Complete	4	0	29
**3901**	Cytochrome bc1 complex respiratory unit	M00151	100	Complete	10	0	83
**3902**	Cytochrome bc1 complex	M00152	100	Complete	10	0	169
**3903**	Cytochrome bd ubiquinol oxidase	M00153	100	Complete	4	0	38

**3905**	Cytochrome c oxidase, prokaryotes	M00155	100	Complete	4	0	68
**3907**	F-type ATPase, prokaryotes and chloroplasts	M00157	100	Complete	8	0	392
**3908**	F-type ATPase, eukaryotes	M00158	100	Complete	18	0	305
**3909**	V/A-type ATPase, prokaryotes	M00159	100	Complete	9	0	246
**3910**	V-type ATPase, eukaryotes	M00160	100	Complete	14	0	253
**3911**	Photosystem II	M00161	100	Complete	6	0	20
**3914**	EHEC pathogenicity signature, Shiga toxin	M00363	100	Complete	2	0	36
**3915**	Fatty acid elongation in endoplasmic reticulum	M00415	75	Partial	13	1	64
**3916**	Cytochrome aa3-600 menaquinol oxidase	M00416	100	Complete	4	0	39
**3918**	EHEC/EPEC pathogenicity signature, T3SS and effectors	M00542	87,5	Partial	17	1	124
**3920**	Pertussis pathogenicity signature, pertussis toxin	M00574	75	Partial	4	1	28
**3921**	Pertussis pathogenicity signature, T1SS	M00575	100	Complete	5	0	141
**3926**	beta-Lactam resistance, Bla system	M00627	100	Complete	4	0	259
**3931**	Multidrug resistance, efflux pump AdeABC	M00649	100	Complete	4	0	80
**3934**	Xanthomonas spp. pathogenicity signature, T3SS and effectors	M00660	75	Partial	18	2	92
**3939**	Multidrug resistance, efflux pump AbcA	M00700	100	Complete	3	0	34
**3940**	Multidrug resistance, efflux pump NorB	M00702	100	Complete	3	0	26
**3942**	Multidrug resistance, efflux pump MepA	M00705	100	Complete	2	0	17
**3943**	Multidrug resistance, efflux pump QacA	M00714	100	Complete	2	0	21
**3944**	Multidrug resistance, efflux pump MexAB-OprM	M00718	100	Complete	4	0	31
**3948**	Cationic antimicrobial peptide (CAMP) resistance, protease PgtE	M00744	100	Complete	3	0	80
**3949**	Imipenem resistance, repression of porin OprD	M00745	100	Complete	6	0	156
**3955**	Carbapenem resistance	M00851	100	Complete	21	0	357
**3960**	Bacillus anthracis pathogenicity signature, anthrax toxin	M00859	100	Complete	3	0	65
**3961**	Bacillus anthracis pathogenicity signature, polyglutamic acid capsule biosynthesis	M00860	71,4	Partial	7	2	14
**3963**	Fatty acid biosynthesis in mitochondria, animals	M00873	100	Complete	8	0	152
**3964**	Fatty acid biosynthesis in mitochondria, fungi	M00874	100	Complete	7	0	152
**2876**	Glycolysis (Embden-Meyerhof pathway), glucose => pyruvate	M00001	100	Complete	31	0	208
**139**	Glycolysis, core module involving three-carbon compounds	M00002	100	Complete	12	0	117
**2091**	Gluconeogenesis, oxaloacetate => fructose-6P	M00003	100	Complete	20	0	81
**2458**	Pentose phosphate pathway (Pentose phosphate cycle)	M00004	100	Complete	15	0	91
**3516**	PRPP biosynthesis, ribose 5P => PRPP	M00005	100	Complete	1	0	2
**838**	Pentose phosphate pathway, oxidative phase, glucose 6P => ribulose 5P	M00006	100	Complete	6	0	31
**2062**	Pentose phosphate pathway, non-oxidative phase, fructose 6P => ribose 5P	M00007	100	Complete	6	0	33
**258**	Entner-Doudoroff pathway, glucose-6P => glyceraldehyde-3P + pyruvate	M00008	100	Complete	5	0	32
**3508**	Citrate cycle (TCA cycle, Krebs cycle)	M00009	100	Complete	36	0	312
**2349**	Citrate cycle, first carbon oxidation, oxaloacetate => 2-oxoglutarate	M00010	100	Complete	6	0	85

**2392**	Citrate cycle, second carbon oxidation, 2-oxoglutarate => oxaloacetate	M00011	100	Complete	30	0	227
**1057**	Glyoxylate cycle	M00012	100	Complete	9	0	139
**3234**	Malonate semialdehyde pathway, propanoyl-CoA => acetyl-CoA	M00013	100	Complete	7	0	48
**2418**	Glucuronate pathway (uronate pathway)	M00014	85,7	Partial	12	1	71
**1921**	Proline biosynthesis, glutamate => proline	M00015	100	Complete	4	0	17
**1451**	Lysine biosynthesis, succinyl-DAP pathway, aspartate => lysine	M00016	100	Complete	13	0	65
**912**	Methionine biosynthesis, aspartate => homoserine => methionine	M00017	100	Complete	13	0	35
**884**	Threonine biosynthesis, aspartate => homoserine => threonine	M00018	100	Complete	10	0	40
**2496**	Valine/isoleucine biosynthesis, pyruvate => valine / 2-oxobutanoate => isoleucine	M00019	100	Complete	6	0	172
**1818**	Serine biosynthesis, glycerate-3P => serine	M00020	100	Complete	6	0	27
**1707**	Cysteine biosynthesis, serine => cysteine	M00021	100	Complete	5	0	42
**1088**	Shikimate pathway, phosphoenolpyruvate + erythrose-4P => chorismate	M00022	100	Complete	14	0	81
**1717**	Tryptophan biosynthesis, chorismate => tryptophan	M00023	100	Complete	15	0	75
**1849**	Phenylalanine biosynthesis, chorismate => phenylpyruvate => phenylalanine	M00024	100	Complete	13	0	95
**409**	Tyrosine biosynthesis, chorismate => HPP => tyrosine	M00025	100	Complete	12	0	63
**3043**	Histidine biosynthesis, PRPP => histidine	M00026	100	Complete	17	0	39
**1305**	GABA (gamma-Aminobutyrate) shunt	M00027	100	Complete	8	0	58
**1366**	Ornithine biosynthesis, glutamate => ornithine	M00028	100	Complete	14	0	136
**2301**	Urea cycle	M00029	100	Complete	6	0	49
**238**	Lysine biosynthesis, AAA pathway, 2-oxoglutarate => 2-aminoadipate => lysine	M00030	87,5	Partial	9	1	26
**3431**	Lysine biosynthesis, mediated by LysW, 2-aminoadipate => lysine	M00031	83,3	Partial	6	1	27
**76**	Ectoine biosynthesis, aspartate => ectoine	M00033	80	Partial	5	1	7
**1065**	Methionine salvage pathway	M00034	100	Complete	20	0	116
**75**	Methionine degradation	M00035	100	Complete	8	0	289
**3328**	Leucine degradation, leucine => acetoacetate + acetyl-CoA	M00036	100	Complete	13	0	227
**1883**	Tryptophan metabolism, tryptophan => kynurenine => 2-aminomuconate	M00038	85,7	Partial	11	1	47
**2103**	Tyrosine biosynthesis, chorismate => arogenate => tyrosine	M00040	100	Complete	8	0	20
**2439**	Thyroid hormone biosynthesis, tyrosine => triiodothyronine/thyroxine	M00043	100	Complete	1	0	3
**1471**	Tyrosine degradation, tyrosine => homogentisate	M00044	100	Complete	9	0	36
**1605**	Histidine degradation, histidine => N-formiminoglutamate => glutamate	M00045	100	Complete	8	0	35
**1591**	De novo purine biosynthesis, PRPP + glutamine => IMP	M00048	87,5	Partial	24	1	94
**2065**	Adenine ribonucleotide biosynthesis, IMP => ADP,ATP	M00049	100	Complete	7	0	53

**623**	Guanine ribonucleotide biosynthesis, IMP => GDP,GTP	M00050	100	Complete	5	0	43
**2767**	De novo pyrimidine biosynthesis, glutamine (+ PRPP) => UMP	M00051	100	Complete	15	0	61
**2549**	Pyrimidine ribonucleotide biosynthesis, UMP => UDP/UTP,CDP/CTP	M00052	100	Complete	6	0	25
**2199**	Deoxyribonucleotide biosynthesis, ADP/GDP/CDP/UDP => dATP/dGTP/dCTP/dUTP	M00053	100	Complete	6	0	51
**469**	Glycosaminoglycan biosynthesis, heparan sulfate backbone	M00059	80	Partial	10	1	24
**2270**	D-Glucuronate degradation, D-glucuronate => pyruvate + D-glyceraldehyde 3P	M00061	80	Partial	7	1	81
**2233**	ADP-L-glycero-D-manno-heptose biosynthesis	M00064	100	Complete	6	0	16
**110**	Sulfoglycolipids biosynthesis, ceramide/1-alkyl-2-acylglycerol => sulfatide/seminolipid	M00067	100	Complete	2	0	2
**2446**	Glycosphingolipid biosynthesis, globo-series, LacCer => Gb4Cer	M00068	100	Complete	2	0	11
**1012**	Heparan sulfate degradation	M00078	75	Partial	9	2	25
**1324**	Pectin degradation	M00081	100	Complete	3	0	20
**1473**	Fatty acid biosynthesis, initiation	M00082	100	Complete	14	0	988
**2140**	Fatty acid biosynthesis, elongation	M00083	100	Complete	14	0	318
**2796**	Fatty acid elongation in mitochondria	M00085	100	Complete	6	0	92
**793**	beta-Oxidation, acyl-CoA synthesis	M00086	100	Complete	2	0	120
**2499**	beta-Oxidation	M00087	100	Complete	19	0	371
**1657**	Ketone body biosynthesis, acetyl-CoA => acetoacetate/3-hydroxybutyrate/acetone	M00088	80	Partial	5	1	97
**2895**	Triacylglycerol biosynthesis	M00089	100	Complete	22	0	209
**572**	Phosphatidylcholine (PC) biosynthesis, choline => PC	M00090	100	Complete	5	0	21
**3176**	Phosphatidylcholine (PC) biosynthesis, PE => PC	M00091	100	Complete	4	0	15
**1436**	Phosphatidylethanolamine (PE) biosynthesis, ethanolamine => PE	M00092	100	Complete	5	0	8
**1724**	Phosphatidylethanolamine (PE) biosynthesis, PA => PS => PE	M00093	100	Complete	4	0	171
**318**	Ceramide biosynthesis	M00094	100	Complete	8	0	75
**1996**	C5 isoprenoid biosynthesis, mevalonate pathway	M00095	71,4	Partial	8	2	39
**2445**	C5 isoprenoid biosynthesis, non-mevalonate pathway	M00096	100	Complete	9	0	66
**2146**	beta-Carotene biosynthesis, GGAP => beta-carotene	M00097	83,3	Partial	6	1	20
**3017**	Acylglycerol degradation	M00098	100	Complete	16	0	354
**252**	Sphingosine biosynthesis	M00099	100	Complete	11	0	111
**2486**	Sphingosine degradation	M00100	100	Complete	2	0	13
**295**	Conjugated bile acid biosynthesis, cholate => taurocholate/glycocholate	M00106	100	Complete	2	0	5
**2107**	C21-Steroid hormone biosynthesis, progesterone => corticosterone/aldosterone	M00108	100	Complete	3	0	7
**442**	C21-Steroid hormone biosynthesis, progesterone => cortisol/cortisone	M00109	75	Partial	5	1	10
**2897**	C19/C18-Steroid hormone biosynthesis, pregnenolone => androstenedione => estrone	M00110	100	Complete	3	0	13

**713**	Tocopherol/tocotorienol biosynthesis, homogentisate + phytyl/geranylgeranyl-PP => tocopherol/tocotorienol	M00112	100	Complete	5	0	10
**833**	Ascorbate biosynthesis, plants, fructose-6P => ascorbate	M00114	75	Partial	8	2	21
**3067**	NAD biosynthesis, aspartate => quinolinate => NAD	M00115	100	Complete	8	0	53
**2410**	Menaquinone biosynthesis, chorismate (+ polyprenyl-PP) => menaquinol	M00116	100	Complete	9	0	101
**1350**	Ubiquinone biosynthesis, prokaryotes, chorismate (+ polyprenyl-PP) => ubiquinol	M00117	77,8	Partial	11	2	52
**211**	Glutathione biosynthesis, glutamate => glutathione	M00118	100	Complete	5	0	35
**2536**	Pantothenate biosynthesis, valine/L-aspartate => pantothenate	M00119	100	Complete	6	0	19
**2211**	Coenzyme A biosynthesis, pantothenate => CoA	M00120	100	Complete	12	0	44
**1187**	Heme biosynthesis, plants and bacteria, glutamate => heme	M00121	80	Partial	15	2	125
**3521**	Cobalamin biosynthesis, cobyrinate a,c-diamide => cobalamin	M00122	100	Complete	10	0	130
**2841**	Biotin biosynthesis, pimeloyl-ACP/CoA => biotin	M00123	100	Complete	6	0	18
**1596**	Riboflavin biosynthesis, plants and bacteria, GTP => riboflavin/FMN/FAD	M00125	100	Complete	17	0	81
**1531**	Tetrahydrofolate biosynthesis, GTP => THF	M00126	100	Complete	17	0	92
**3166**	Thiamine biosynthesis, prokaryotes, AIR (+ DXP/tyrosine) => TMP/TPP	M00127	71,4	Partial	11	2	483
**1174**	Ascorbate biosynthesis, animals, glucose-1P => ascorbate	M00129	100	Complete	7	0	87
**1557**	Inositol phosphate metabolism, PI=> PIP2 => Ins(1,4,5)P3 => Ins(1,3,4,5)P4	M00130	100	Complete	12	0	266
**2379**	Inositol phosphate metabolism, Ins(1,3,4,5)P4 => Ins(1,3,4)P3 => myo-inositol	M00131	75	Partial	4	1	24
**2302**	Polyamine biosynthesis, arginine => agmatine => putrescine => spermidine	M00133	100	Complete	7	0	63
**963**	Polyamine biosynthesis, arginine => ornithine => putrescine	M00134	100	Complete	2	0	16
**3344**	GABA biosynthesis, eukaryotes, putrescine => GABA	M00135	100	Complete	5	0	113
**1861**	GABA biosynthesis, prokaryotes, putrescine => GABA	M00136	100	Complete	4	0	45
**836**	Flavanone biosynthesis, phenylalanine => naringenin	M00137	80	Partial	5	1	37
**2548**	C1-unit interconversion, prokaryotes	M00140	100	Complete	5	0	13
**1998**	C1-unit interconversion, eukaryotes	M00141	100	Complete	4	0	16
**3283**	Reductive pentose phosphate cycle (Calvin cycle)	M00165	90,9	Partial	18	1	89
**1275**	CAM (Crassulacean acid metabolism), dark	M00168	100	Complete	4	0	38
**3041**	CAM (Crassulacean acid metabolism), light	M00169	100	Complete	2	0	22
**1267**	C4-dicarboxylic acid cycle, phosphoenolpyruvate carboxykinase type	M00170	100	Complete	4	0	35
**3243**	C4-dicarboxylic acid cycle, NAD - malic enzyme type	M00171	85,7	Partial	9	1	44
**1824**	C4-dicarboxylic acid cycle, NADP - malic enzyme type	M00172	100	Complete	4	0	35

**2262**	Reductive citrate cycle (Arnon-Buchanan cycle)	M00173	80	Partial	35	2	142
**112**	Methane oxidation, methanotroph, methane => formaldehyde	M00174	100	Complete	11	0	13
**3496**	Assimilatory sulfate reduction, sulfate => H2S	M00176	100	Complete	11	0	85
**1089**	Pyruvate oxidation, pyruvate => acetyl-CoA	M00307	100	Complete	11	0	55
**2055**	Semi-phosphorylative Entner-Doudoroff pathway, gluconate => glycerate-3P	M00308	75	Partial	7	1	130
**799**	Cysteine biosynthesis, homocysteine + serine => cysteine	M00338	100	Complete	3	0	10
**749**	Formaldehyde assimilation, xylulose monophosphate pathway	M00344	100	Complete	4	0	11
**2368**	Formaldehyde assimilation, ribulose monophosphate pathway	M00345	100	Complete	8	0	28
**3407**	Formaldehyde assimilation, serine pathway	M00346	88,9	Partial	10	1	67
**2612**	Coenzyme M biosynthesis	M00358	75	Partial	5	1	14
**3065**	C10-C20 isoprenoid biosynthesis, bacteria	M00364	100	Complete	4	0	6
**2274**	C10-C20 isoprenoid biosynthesis, archaea	M00365	100	Complete	2	0	3
**1706**	C10-C20 isoprenoid biosynthesis, plants	M00366	100	Complete	4	0	10
**1740**	C10-C20 isoprenoid biosynthesis, non-plant eukaryotes	M00367	100	Complete	3	0	7
**493**	Ethylmalonyl pathway	M00373	91,7	Partial	13	1	173
**2430**	Dicarboxylate-hydroxybutyrate cycle	M00374	84,6	Partial	22	2	125
**1978**	Hydroxypropionate-hydroxybutylate cycle	M00375	92,9	Partial	19	1	156
**2371**	3-Hydroxypropionate bi-cycle	M00376	100	Complete	21	0	857
**1188**	F420 biosynthesis, archaea	M00378	100	Complete	5	0	106
**3177**	Leucine biosynthesis, 2-oxoisovalerate => 2-oxoisocaproate	M00432	100	Complete	5	0	45
**2817**	Lysine biosynthesis, acetyl-DAP pathway, aspartate => lysine	M00525	88,9	Partial	9	1	25
**413**	Lysine biosynthesis, DAP dehydrogenase pathway, aspartate => lysine	M00526	83,3	Partial	9	1	37
**3439**	Lysine biosynthesis, DAP aminotransferase pathway, aspartate => lysine	M00527	100	Complete	10	0	46
**997**	Denitrification, nitrate => nitrogen	M00529	75	Partial	10	1	42
**1014**	Assimilatory nitrate reduction, nitrate => ammonia	M00531	100	Complete	8	0	28
**923**	Photorespiration	M00532	90	Partial	14	1	257
**2674**	Homoprotocatechuate degradation, homoprotocatechuate => 2-oxohept-3-enedioate	M00533	75	Partial	4	1	35
**720**	Isoleucine biosynthesis, pyruvate => 2-oxobutanoate	M00535	100	Complete	4	0	40
**1138**	Benzoate degradation, cyclohexanecarboxylic acid =>pimeloyl-CoA	M00540	100	Complete	5	0	105
**3204**	Trans-cinnamate degradation, trans-cinnamate => acetyl-CoA	M00545	83,3	Partial	11	1	139
**2463**	Purine degradation, xanthine => urea	M00546	100	Complete	20	0	103
**2608**	Nucleotide sugar biosynthesis, glucose => UDP-glucose	M00549	100	Complete	10	0	88
**90**	Ascorbate degradation, ascorbate => D-xylulose-5P	M00550	100	Complete	7	0	49
**99**	Betaine biosynthesis, choline => betaine	M00555	100	Complete	6	0	66

**1687**	Trehalose biosynthesis, D-glucose 1P => trehalose	M00565	100	Complete	7	0	34
**2474**	Catechol ortho-cleavage, catechol => 3-oxoadipate	M00568	100	Complete	5	0	82
**2774**	Catechol meta-cleavage, catechol => acetyl-CoA / 4-methylcatechol => propanoyl-CoA	M00569	80	Partial	12	1	71
**2482**	Isoleucine biosynthesis, threonine => 2-oxobutanoate => isoleucine	M00570	100	Complete	8	0	193
**1253**	Pimeloyl-ACP biosynthesis, BioC-BioH pathway, malonyl-ACP => pimeloyl-ACP	M00572	83,3	Partial	10	1	209
**521**	Biotin biosynthesis, BioI pathway, long-chain-acyl-ACP => pimeloyl-ACP => biotin	M00573	100	Complete	5	0	18
**1606**	Biotin biosynthesis, BioW pathway, pimelate => pimeloyl-CoA => biotin	M00577	100	Complete	6	0	24
**223**	Phosphate acetyltransferase-acetate kinase pathway, acetyl-CoA => acetate	M00579	100	Complete	4	0	24
**3048**	Pentose phosphate pathway, archaea, fructose 6P => ribose 5P	M00580	100	Complete	5	0	20
**461**	2-Oxocarboxylic acid chain extension, 2-oxoglutarate => 2-oxoadipate => 2-oxopimelate => 2-oxosuberate	M00608	100	Complete	4	0	33
**1365**	Incomplete reductive citrate cycle, acetyl-CoA => oxoglutarate	M00620	71,4	Partial	17	2	46
**906**	D-Galacturonate degradation (fungi), D-galacturonate => glycerol	M00630	75	Partial	5	1	8
**357**	Galactose degradation, Leloir pathway, galactose => alpha-D-glucose-1P	M00632	75	Partial	4	1	18
**2511**	Semi-phosphorylative Entner-Doudoroff pathway, gluconate/galactonate => glycerate-3P	M00633	75	Partial	4	1	27
**895**	Anthranilate degradation, anthranilate => catechol	M00637	100	Complete	7	0	11
**1897**	Methylaspartate cycle	M00740	90,9	Partial	12	1	79
**1254**	Propanoyl-CoA metabolism, propanoyl-CoA => succinyl-CoA	M00741	100	Complete	13	0	117
**2157**	Ornithine biosynthesis, mediated by LysW, glutamate => ornithine	M00763	83,3	Partial	6	1	27
**2869**	Dihydrokalafungin biosynthesis, octaketide => dihydrokalafungin	M00779	75	Partial	8	1	9
**355**	dTDP-L-rhamnose biosynthesis	M00793	100	Complete	5	0	29
**922**	Nicotine degradation, pyridine pathway, nicotine => 2,6-dihydroxypyridine/succinate semialdehyde	M00810	75	Partial	9	1	11
**2968**	C-1027 beta-amino acid moiety biosynthesis, tyrosine => 3-chloro-4,5-dihydroxy-beta-phenylalanyl-PCP	M00827	83,3	Partial	6	1	25
**368**	Pyocyanine biosynthesis, chorismate => pyocyanine	M00835	71,4	Partial	7	2	14
**1965**	Tetrahydrobiopterin biosynthesis, GTP => BH4	M00842	100	Complete	3	0	14
**2545**	L-threo-Tetrahydrobiopterin biosynthesis, GTP => L-threo-BH4	M00843	100	Complete	3	0	68
**2036**	Arginine biosynthesis, ornithine => arginine	M00844	100	Complete	4	0	40
**3138**	Arginine biosynthesis, glutamate => acetylcitrulline => arginine	M00845	85,7	Partial	7	1	50

**735**	Siroheme biosynthesis, glutamyl-tRNA => siroheme	M00846	83,3	Partial	14	1	67
**1934**	C5 isoprenoid biosynthesis, mevalonate pathway, archaea	M00849	100	Complete	15	0	69
**2414**	Glycogen biosynthesis, glucose-1P => glycogen/starch	M00854	100	Complete	11	0	59
**1743**	Glycogen degradation, glycogen => glucose-6P	M00855	100	Complete	10	0	102
**3073**	beta-Oxidation, peroxisome, VLCFA	M00861	100	Complete	4	0	29
**1807**	KDO2-lipid A modification pathway	M00867	80	Partial	6	1	31
**2444**	Heme biosynthesis, animals and fungi, glycine => heme	M00868	87,5	Partial	8	1	19
**2475**	O-glycan biosynthesis, mannose type (core M3)	M00872	70	Partial	10	3	36
**828**	Staphyloferrin B biosynthesis, L-serine => staphyloferrin B	M00875	71,4	Partial	7	2	9
**2029**	Phenylacetate degradation, phenylaxetate => acetyl-CoA/succinyl-CoA	M00878	85,7	Partial	11	1	226
**1303**	Molybdenum cofactor biosynthesis, GTP => molybdenum cofactor	M00880	100	Complete	9	0	54
**948**	Lipoic acid biosynthesis, plants and bacteria, octanoyl-ACP => dihydrolipoyl-E2/H	M00881	100	Complete	2	0	2
**1563**	Lipoic acid biosynthesis, eukaryotes, octanoyl-ACP => dihydrolipoyl-H	M00882	100	Complete	2	0	2
**1619**	Lipoic acid biosynthesis, animals and bacteria, octanoyl-ACP => dihydrolipoyl-H => dihydrolipoyl-E2	M00883	100	Complete	5	0	7
**2193**	UDP-N-acetyl-D-glucosamine biosynthesis, eukaryotes, glucose => UDP-GlcNAc	M00892	100	Complete	8	0	58
**2390**	Thiamine biosynthesis, archaea, AIR (+ NAD+) => TMP/TPP	M00896	75	Partial	7	1	89
**2162**	Thiamine salvage pathway, HMP/HET => TMP	M00899	100	Complete	5	0	86
**2057**	UDP-N-acetyl-D-glucosamine biosynthesis, prokaryotes, glucose => UDP-GlcNAc	M00909	100	Complete	8	0	62
**1119**	Phenylalanine biosynthesis, chorismate => arogenate => phenylalanine	M00910	100	Complete	3	0	13
**2622**	Riboflavin biosynthesis, fungi, GTP => riboflavin/FMN/FAD	M00911	87,5	Partial	8	1	20
**449**	NAD biosynthesis, tryptophan => quinolinate => NAD	M00912	87,5	Partial	13	1	61
**652**	Pantothenate biosynthesis, 2-oxoisovalerate/spermine => pantothenate	M00913	100	Complete	5	0	59
**1686**	Coenzyme A biosynthesis, archaea, 2-oxoisovalerate => 4-phosphopantoate => CoA	M00914	85,7	Partial	7	1	18
**1178**	Pyridoxal-P biosynthesis, R5P + glyceraldehyde-3P + glutamine => pyridoxal-P	M00916	100	Complete	2	0	7
**2333**	Ectoine degradation, ectoine => aspartate	M00919	75	Partial	4	1	7
**3026**	Cobalamin biosynthesis, anaerobic, uroporphyrinogen III => sirohydrochlorin => cobyrinate a,c-diamide	M00924	90,9	Partial	21	1	68
**2713**	Cobalamin biosynthesis, aerobic, uroporphyrinogen III => precorrin 2 => cobyrinate a,c-diamide	M00925	90,9	Partial	16	1	126
**2807**	Gibberellin A4/A1 biosynthesis, GA12/GA53 => GA4/GA1	M00928	100	Complete	2	0	3

**3275**	Menaquinone biosynthesis, futalosine pathway	M00930	100	Complete	7	0	15
**880**	Menaquinone biosynthesis, modified futalosine pathway	M00931	80	Partial	5	1	8
**1615**	Phylloquinone biosynthesis, chorismate (+ phytyl-PP) => phylloquinol	M00932	85,7	Partial	13	1	135
**3064**	Plastoquinone biosynthesis, homogentisate + solanesyl-PP => plastoquinol	M00933	100	Complete	2	0	3
**2784**	Melatonin biosynthesis, plants, tryptophan => serotonin => melatonin	M00936	75	Partial	5	1	19

Table S3. KEGG modules uniquely identified in the genome of *Rhodococcus* sp. GB-02 compared with *Rhodococcus erythropolis* IEGM 1415 (GCF_027270535.1) and *Rhodococcus* sp. IEGM 1318 (GCF_033142465.1).

Module	Metabolic pathway
**M00059**	Glycosaminoglycan biosynthesis, heparan sulfate backbone
**M00067**	Sulfoglycolipids biosynthesis, ceramide/1-alkyl-2-acylglycerol ⇒ sulfatide/seminolipid
**M00068**	Glycosphingolipid biosynthesis, globo-series, LacCer ⇒ Gb4Cer
**M00090**	Phosphatidylcholine (PC) biosynthesis, choline ⇒ phosphatidylcholine
**M00097**	β-Carotene biosynthesis, GGPP ⇒ β-carotene
**M00108**	C21-steroid hormone biosynthesis, progesterone ⇒ corticosterone/aldosterone
**M00109**	C21-steroid hormone biosynthesis, progesterone ⇒ cortisol/cortisone
**M00118**	Glutathione biosynthesis, glutamate ⇒ glutathione
**M00137**	Flavanone biosynthesis, phenylalanine ⇒ naringenin
**M00140**	C1-unit interconversion, prokaryotes
**M00152**	Cytochrome bc1 complex
**M00161**	Photosystem II
**M00649**	Multidrug resistance, efflux pump AdeABC
**M00660**	*Xanthomonas* spp. pathogenicity signature, Type III secretion system (T3SS) and effectors
**M00718**	Multidrug resistance, efflux pump MexAB-OprM
**M00744**	Cationic antimicrobial peptide (CAMP) resistance, protease PgtE
**M00810**	Nicotine degradation, pyridine pathway, nicotine ⇒ 2,6-dihydroxypyridine/succinate semialdehyde
**M00827**	C-1027 β-amino acid moiety biosynthesis, tyrosine ⇒ 3-chloro-4,5-dihydroxy-β-phenylalanyl-PCP
**M00835**	Pyocyanine biosynthesis, chorismate ⇒ pyocyanine
**M00849**	C5 isoprenoid biosynthesis, mevalonate pathway (archaea)
**M00860**	*Bacillus anthracis* pathogenicity signature, polyglutamic acid capsule biosynthesis
**M00875**	Staphyloferrin B biosynthesis, L-serine ⇒ staphyloferrin B
**M00896**	Thiamine biosynthesis, archaea, AIR (+ NAD^+) ⇒ TMP/TPP
**M00928**	Gibberellin A4/A1 biosynthesis, GA12/GA53 ⇒ GA4/GA1
**M00930**	Menaquinone biosynthesis, futalosine pathway
**M00931**	Menaquinone biosynthesis, modified futalosine pathway

Table S4. Distribution of biosynthetic gene cluster (BGC) subclasses across GB-02 and *Rhodococcus* genomes.

**Genomes**	**Total count**	**CDPS**	**HR-T2PKS**	**NAPAA**	**NRPS**	**NRPS-like**	**RRE-containing**	**RiPP-like**	**T1PKS**	**T2PKS**	**T3PKS**	**aminopolycarboxylic-acid**	**arylpolyene**	**azole-containing-RiPP**	**betalactone**	**bottromycin**	**butyrolactone**	**ectoine**	**furan**	**hglE-KS**	**hserlactone**	**lanthipeptide-class-iii**	**lanthipeptide-class-iv**	**lanthipeptide-class-v**	**lassopeptide**	**nucleoside**	**other**	**phosphonate**	**polyhalogenated-pyrrole**	**redox-cofactor**	**terpene**	**terpene-precursor**	**triceptide**	**hybrid**
***R. wratislaviensis* NCTC 13229**	32	0	0	1	15	0	0	2	2	0	0	0	0	0	3	0	2	1	0	0	0	0	0	0	0	0	0	0	0	1	2	0	0	3
***Rhodococcus* sp. NJ-530**	19	0	0	2	3	1	0	1	2	0	0	0	0	1	0	0	1	1	0	0	0	1	0	0	0	0	0	0	0	1	1	0	0	4
***R. pseudokoreensis* R79T**	30	0	0	1	13	0	0	2	2	0	0	0	0	0	4	0	1	1	0	0	0	0	0	0	0	1	0	0	0	1	1	0	0	3
***R. navarretei* EXRC-4A-4T**	16	0	0	1	4	1	0	0	2	0	0	0	0	0	1	0	1	1	0	0	0	0	0	0	0	0	0	0	0	0	2	0	0	3
***R. trifolii* CCM 7905**	19	0	0	2	5	1	0	1	1	0	1	0	1	0	1	0	0	1	0	0	0	0	0	0	0	0	0	0	0	1	2	0	0	2
***R. triatomae* DSM 44892**	15	0	0	1	1	1	0	2	1	0	1	0	0	0	0	0	1	1	0	0	0	0	0	0	0	0	0	0	0	1	2	0	0	3
***R. spelaei* C9-5**	15	0	0	2	3	1	0	1	1	0	0	0	0	0	1	0	0	1	0	0	0	0	0	0	0	0	1	0	0	1	2	0	0	1
***Rhodococcus* sp. UYP9**	19	0	0	1	8	3	0	0	1	0	0	0	0	0	0	0	0	1	0	0	0	0	0	0	0	0	0	0	0	1	2	0	0	2
***R. parequi* PAM 2766**	17	0	0	1	6	0	0	1	1	0	0	0	0	0	0	0	1	1	0	0	0	0	0	0	0	0	0	0	0	1	3	0	0	2
***R. coprophilus* NCTC 10994**	12	0	0	1	2	0	0	0	1	0	0	0	0	0	1	0	0	1	0	0	0	0	0	0	0	0	0	0	0	0	3	1	0	2
***Rhodococcus* sp. WY5**	19	0	0	1	3	1	0	1	2	0	0	0	0	1	0	0	1	1	0	0	0	1	0	0	0	0	0	0	0	1	1	0	0	5
***R. kroppenstedtii* DSM 44908**	13	0	0	1	3	2	0	0	1	0	1	0	0	0	0	0	0	1	0	0	0	0	0	0	0	0	0	0	0	0	2	0	0	2
***R. jostii* DSM 44719**	27	0	0	1	10	0	0	1	1	0	1	0	0	0	1	0	2	1	0	1	0	0	0	0	0	0	0	0	0	1	2	0	0	5
***R. kyotonense* JCM 23211**	25	0	0	1	7	1	0	1	2	0	0	0	1	1	2	0	0	1	0	0	0	0	0	0	0	0	0	0	0	1	2	0	0	5

R. equi **DSM 20307**	19	0	0	1	7	0	0	1	1	0	0	0	0	0	0	0	1	1	0	0	0	0	0	0	0	0	0	0	0	1	4	0	0	2
**GB-02**	20	0	0	1	5	1	0	1	1	0	0	0	0	1	0	0	1	1	0	1	0	1	0	0	0	0	0	0	0	1	1	0	0	4
Rhodococcus **sp. PAMC28705**	12	0	0	1	3	1	0	0	1	0	0	0	0	0	0	0	1	1	0	0	0	0	0	0	0	0	0	0	0	0	2	0	0	2
R. yananensis **FBM22-1**	19	0	0	1	9	0	0	1	1	0	0	0	0	0	1	0	1	1	0	0	0	0	0	0	0	0	0	0	0	1	2	0	0	1
R. aerolatus **JCM 19485**	6	0	0	0	0	0	0	0	1	0	1	0	0	0	0	0	0	0	0	0	0	0	0	0	0	0	0	0	0	1	3	0	0	0
R. kronopolitis **CGMCC 4.7145**	15	0	0	1	5	1	0	0	1	0	0	0	0	0	0	0	0	2	0	0	0	0	0	0	0	0	0	0	0	1	2	0	0	2
Rhodococcus **sp. ANT H53B**	15	0	0	1	4	2	0	0	1	0	0	0	1	0	0	0	1	1	0	0	0	0	0	0	0	0	0	0	0	1	2	0	0	1
R. opacus **DSM 43205**	30	0	0	1	14	1	0	3	2	0	0	0	0	0	1	0	2	1	0	0	0	0	0	0	0	0	0	0	0	1	3	0	0	1
R. qingshengii **Ray**	18	0	0	1	3	1	0	1	2	0	0	0	0	1	0	0	1	1	0	0	0	1	0	0	0	0	0	0	0	1	1	0	0	4
R. xishaensis **LHW51113**	20	0	0	1	8	2	0	1	2	0	0	0	0	0	0	0	0	0	0	0	0	0	0	0	0	0	0	0	0	1	3	0	0	2
R. subtropicus **C9-28**	13	0	0	1	4	0	0	1	1	0	0	0	0	0	0	1	0	1	0	0	0	0	0	0	0	0	0	0	0	0	3	0	0	1
R. gordoniae **NCTC 13296**	13	0	0	1	2	1	0	0	1	0	0	0	0	0	1	0	0	1	0	0	0	0	0	0	0	0	0	0	0	0	3	0	0	3
R. maanshanensis **DSM 44675**	20	0	0	2	6	1	0	1	3	0	0	0	0	0	0	0	2	1	0	0	0	0	0	0	0	0	0	0	0	1	2	0	0	1
R. erythropolis **IEGM 1415**	23	0	0	1	5	2	0	1	1	0	0	0	0	1	1	0	1	1	0	1	0	1	1	0	1	0	0	0	0	1	1	0	0	3
R. yunnanensis **NBRC 103083**	27	1	0	1	10	1	0	2	2	0	0	0	1	0	1	0	0	1	0	0	0	0	0	0	0	0	0	0	0	0	2	0	0	5
R. agglutinans **CFHS 0262**	21	0	0	2	7	2	0	2	1	0	0	0	0	0	1	0	0	1	0	0	0	0	0	0	0	0	0	0	0	2	3	0	0	0
R. spongiicola **LHW50502**	22	0	0	1	9	3	0	1	2	0	0	0	0	0	0	0	0	0	0	0	0	0	0	0	0	0	0	0	0	1	3	0	0	2
R. ruber **NBRC 15591**	21	0	0	1	6	1	0	0	2	0	0	0	0	0	2	0	1	1	0	0	0	0	0	0	0	0	0	0	0	1	4	0	0	2
Rhodococcus **sp. PAMC28707**	13	0	0	1	3	1	0	0	1	0	0	0	0	0	0	0	1	1	0	0	0	0	0	0	0	0	0	0	0	0	2	0	0	3
R. sacchari **Z13**	15	0	0	1	3	0	0	1	2	0	0	0	0	0	1	0	0	1	0	0	0	0	0	0	0	0	0	0	0	0	3	0	0	3

***R. aetherivorans* DSM 44752**	28	0	0	1	11	2	1	0	1	0	1	0	0	0	0	0	1	1	0	0	0	0	0	0	1	0	0	0	0	0	4	0	0	4
***Rhodococcus* sp. KRD197**	23	0	0	1	9	1	0	0	1	1	0	0	0	1	0	0	0	1	0	1	0	0	0	0	0	0	0	0	0	1	2	0	0	4
***R. tukisamuensis* JCM 11308**	19	0	0	1	8	1	0	1	1	0	0	0	0	0	0	0	1	1	0	0	0	0	0	0	0	0	0	0	0	1	2	0	0	2
***Rhodococcus* sp. IEGM 1318**	20	0	0	1	5	2	0	1	1	0	0	0	0	1	0	0	1	1	0	1	0	1	0	0	0	0	0	0	0	1	1	0	0	3
***Rhodococcus* sp. H29-C3**	25	0	0	1	8	3	0	0	1	2	0	0	0	0	0	0	1	1	0	1	0	0	0	0	0	0	0	0	0	0	2	0	0	5
***R. defluvii* Ca11**	14	0	0	1	5	0	0	1	1	0	0	0	0	0	1	0	0	1	0	0	0	0	0	0	0	0	0	0	0	1	2	0	0	1
***R. rhodnii* ATCC 35071**	13	0	0	1	1	0	0	2	2	0	0	0	0	0	0	0	1	1	0	0	0	0	0	0	0	0	0	0	0	0	2	0	0	3
***Rhodococcus* sp. G-MC3**	23	0	0	1	5	3	0	1	2	0	0	0	0	0	1	0	1	1	1	1	0	0	0	1	0	0	0	0	0	0	2	0	0	3
***Rhodococcus* sp. UYP5**	22	0	0	1	9	4	0	0	1	0	0	0	0	0	0	0	0	2	0	0	0	0	0	0	0	0	0	0	0	1	2	0	0	2
***R. gannanensis* CGMCC 1.15992**	17	0	0	2	4	1	0	0	1	0	1	0	0	0	0	0	0	2	0	0	0	0	0	0	0	0	0	0	0	1	2	0	0	3
***R. marinonascens* NBRC 14363**	26	0	0	1	17	2	0	0	2	0	0	0	0	0	0	0	0	1	0	0	0	0	0	0	0	0	0	0	0	1	1	0	0	1
***R. erythropolis* JCM 3201**	19	0	0	1	3	1	0	1	2	0	0	0	0	1	0	0	1	1	0	0	0	1	0	0	0	0	0	0	0	1	1	0	0	5
***Rhodococcus* sp. ADH**	26	0	0	2	9	2	0	1	2	0	0	0	0	1	0	0	1	1	0	0	0	1	0	0	0	0	0	0	0	1	1	0	0	4
***R. artemisiae* YIM65754**	17	0	0	1	5	0	0	1	1	0	0	0	0	0	0	0	1	1	0	0	0	0	0	0	0	0	0	0	0	1	2	0	0	4
***R. pyridinivorans* DSM 44555**	12	0	0	1	3	1	0	0	1	0	0	0	0	0	0	0	0	1	0	0	0	0	0	0	0	0	0	0	0	0	2	0	0	3
***R. zopfii* NBRC 100606**	21	0	0	1	7	2	0	0	1	0	0	0	0	0	1	0	0	1	0	0	0	0	0	1	0	0	0	0	0	1	2	0	0	4
***R. olei* JCM 32206**	16	0	0	1	6	1	0	0	1	0	0	0	0	0	1	0	0	1	0	0	0	0	0	0	0	0	0	0	0	1	2	0	0	2
***R. fascians* NBRC 12155**	22	0	0	1	4	3	0	1	2	0	0	0	1	1	1	0	2	1	0	0	0	0	0	0	0	0	0	1	0	0	2	0	0	2
***Rhodococcus* sp. BP22**	16	0	0	1	7	2	0	0	1	0	0	0	0	0	0	0	0	1	0	0	0	0	0	0	0	0	0	0	0	0	2	0	0	2
***R. globerulus* NBRC 14531**	21	0	0	2	5	1	0	1	1	0	1	0	0	0	1	0	0	1	0	0	0	0	0	0	0	0	0	0	0	1	1	1	0	5

***R. rhodochrous* NCTC 10210**	15	0	0	1	5	0	0	0	1	0	0	0	0	0	1	0	1	1	0	0	0	0	0	0	0	0	0	0	0	0	3	0	0	2
***Rhodococcus* sp. KRD162**	20	0	1	1	5	4	0	0	1	0	0	0	0	0	0	0	1	1	0	0	0	0	0	0	1	0	0	0	0	1	2	0	0	2
***Rhodococcus* sp. AQ5-07**	19	0	0	1	3	1	0	1	2	0	0	0	0	1	0	0	1	1	0	0	0	1	0	0	0	0	0	0	0	1	1	0	0	5
***R. koreensis* DSM 44498**	35	0	0	1	15	0	0	2	2	0	1	0	0	0	3	0	2	1	1	0	0	0	0	0	0	0	0	0	1	1	2	0	1	2
***R. oxybenzonivorans* S2-17**	25	0	0	1	11	1	0	1	2	0	0	1	0	0	2	0	0	1	0	0	1	0	0	0	0	0	0	0	0	1	1	0	0	2
***R. qingshengii* A29-k1**	18	0	0	1	3	1	0	1	2	0	0	0	0	1	0	0	1	1	0	0	0	1	0	0	0	0	0	0	0	1	1	0	0	4
***R. soli* DSM 46662**	19	0	0	1	7	1	0	1	1	0	0	0	0	0	1	0	0	1	0	0	0	0	0	0	0	0	0	0	0	1	3	0	0	2
***R. chondri* CC-R104**	25	0	0	1	11	3	0	1	1	0	0	0	0	0	0	0	0	1	0	0	0	0	0	0	0	0	0	0	0	1	4	0	0	2
***R. antarcticus* 75**	18	0	0	1	3	1	0	1	2	0	0	0	0	1	0	0	1	1	0	0	0	1	0	0	0	0	0	0	0	1	1	0	0	4
***R. phenolicus* DSM 44812**	24	0	0	1	11	2	0	1	2	0	0	0	0	0	0	0	0	1	0	0	0	0	0	0	0	0	0	0	0	1	2	0	0	3
***Rhodococcus* sp. H36-A4**	15	0	0	1	5	2	0	0	0	0	0	0	0	0	0	0	0	1	0	0	0	0	0	0	0	0	0	0	0	0	2	0	0	4
***R. oryzae* NEAU-CX67T**	20	0	0	1	7	1	0	1	2	0	0	0	0	0	0	0	1	1	0	0	0	0	0	0	0	0	0	0	0	1	2	0	0	3
***R. indonesiensis* CSLK01-03**	32	0	0	1	14	4	0	0	2	0	0	0	0	0	1	0	1	1	0	0	0	0	0	0	0	0	0	0	0	1	4	0	0	3
***R. corynebacterioides* NBRC 14404**	12	0	0	1	2	2	0	0	1	0	1	0	0	0	0	0	0	1	0	0	0	0	0	0	0	0	0	0	0	0	2	0	0	2

Table S6. Regulatory genes identified within BGCs of strain GB-02 based on antiSMASH analysis

Region	BGC type	Regulatory genes	Location	Total (nt)	Best BLASTp hit	Identity (%)	NCBI accession
**1.1**	RiPP-like	*AsnC* family transcriptional regulator	276,711 - 277,181	471	Putative *AsnC* family transcriptional regulator	98.08	BAH36695.1
**1.2**	LAP	*LuxR* family DNA-binding response regulator	438,646 - 439,263	618	*LuxR* C-terminal-related transcriptional regulator	93.17	WP_113683935.1
		Sensor histidine kinase	439,260 - 440,462	1203	Sensor histidine kinase	96.74	WP_378505040.1
		TetR family transcriptional regulator	462,029 - 462,661	633	*TetR*/*AcrR* family transcriptional regulator	98.09	WP_182262313.1
**1.3**	T1PKS	*GntR* family transcriptional regulator	581,509 - 582,297<br>591,755 - 592,525	789<br>771	*GntR* family transcriptional regulator	98.85<br>97.66	WP_347875738.1<br>WP_387387079.1
		Autoinducer-binding transcriptional regulator	596,354 - 597,337	984	*LuxR* C-terminal-related transcriptional regulator	97.55	WP_166811757.1
		LysR family transcriptional regulator	598,316 - 599,215	900	*LysR* family transcriptional regulator	98.66	WP_210377933.1
**1.4**	NAPAA	*GntR* family transcriptional regulator	894,344 - 894,988	645	*GntR* family transcriptional regulator	96.73	WP_029254358.1
**1.5**	NRP-metallophore,NRPS	Response regulator	1,324,771 - 1,325,502	732	Two-component system, *OmpR* family, response regulator	98.77	SCC60063.1
		Sensor histidine kinase	1,325,499 - 1,326,950	1452	Sensor histidine kinase	97.93	WP_378547729.1
**1.6**	Redox-cofactor	*LuxR* family DNA-binding response regulator	2,113,168 - 2,113,851	684	*MadR* family response regulator transcription factor	99.12	WP_317552380.1
		TetR family transcriptional regulator	2,121,119 - 2,121,718	600	Mycofactocin system transcriptional regulator *MftR*	98.99	WP_003941984.1
**1.7**	NRPS	*TetR* family transcriptional regulator	2,161,811 - 2,162,410	600	SACE_7040 family transcriptional regulator	92.93	WP_092801702.1
**1.8**	NRPS-like	*TetR* family transcriptional regulator	2,673,011 - 2,673,610	600	*TetR*/*AcrR* family transcriptional regulator	100.00	WP_452624036.1
**2.1**	Butyrolactone	*TetR* family transcriptional regulator	1,296,601 - 1,297,245	645	*ScbR* family autoregulator-binding transcription factor	99.07	WP_378446112.1
		ArsR family transcriptional regulator	1,300,024 - 1,300,368	345	*ArsR*/*SmtB* family transcription factor	94.74	WP_138870742.1

**2.2**	Lanthipeptide-class-iii	*TetR* family transcriptional regulator	1,516,639 - 1,517,244	606	Hypothetical protein GCM10020255_034230	98.01	GAA3153997.1
**3.1**	NRPS	Sensor histidine kinase	135,120 - 136,247	1128	Sensor histidine kinase	98.67	WP_378648122.1
		LuxR family DNA-binding response regulator	136,305 - 136,964	660	Response regulator	92.69	WP_138868608.1
		ArsR family transcriptional regulator	177,426 - 178,223	798	*ArsR*/*SmtB* family transcription factor	94.72	WP_083057162.1
**3.2**	NRPS,terpene	*TetR* family transcriptional regulator	229,949 - 230,515	567	*TetR*/*AcrR* family transcriptional regulator	93.62	WP_358273986.1
**3.3**	NRP-metallophore, NRPS	RNA polymerase, sigma-24 subunit, ECF subfamily	603,259 - 603,843	585	RNA polymerase sigma factor	91.75	WP_214886392.1
		LysR family transcriptional regulator	613,562 - 614,554	993	*LysR* family transcriptional regulator	99.39	WP_269590771.1
		AraC family transcriptional regulator	646,452 - 647,240	789	*AraC* family transcriptional regulator	90.44	RAL31693.1
		Sigma-54 dependent trancsriptional regulator	648,550 - 650,370	1821	Sigma-54-dependent Fis family transcriptional regulator	99.83	WP_317828623.1
**4.1**	NRPS	Sensor histidine kinase	153,679 - 155,373	1695	Sensor histidine kinase	99.62	WP_317829220.1
		LuxR family DNA-binding response regulator	155,370 - 156,014	645	Response regulator transcription factor	100.00	WP_317829221.1
**4.2**	hglE-KS	*LysR* family transcriptional regulator	194,744 - 195,676	933	*LysR* family transcriptional regulator	96.45	WP_338890965.1
**4.3**	NRPS	*TetR* family transcriptional regulator	270,125 - 270,910	786	*TetR*/*AcrR* family transcriptional regulator	99.62	WP_347875785.1
			308,485 - 309,138	654	*TetR*/*AcrR* family transcriptional regulator	99.02	WP_411194328.1
		Sensor histidine kinase	293,370 - 294,605	1236	Sensor histidine kinase	96.60	WP_283569290.1
		Response regulator	294,602 - 295,345	744	Response regulator transcription factor	99.19	WP_347872467.1
		ArsR family transcriptional regulator	303,806 - 304,153	348	Transcriptional regulator	98.26	MDF2468936.1
**4.4**	NRPS	*AraC* family transcriptional regulator	322,730 - 323,623	894	*AraC* family transcriptional regulator	93.26	WP_378547923.1
		LysR family transcriptional regulator	360,744 - 361,652	909	*LysR* substrate-binding domain-containing protein	93.38	WP_387481597.1
		TetR family transcriptional regulator	366,423 - 367,025	603	*TetR*/*AcrR* family transcriptional regulator	97.99	WP_317554101.1

**4.5**	Terpene	Serine/threonine protein kinase	461,033 - 463,084	2052	Stk1 family PASTA domain-containing Ser/Thr kinase	97.95	WP_243617319.1
**4.6**	Arylpolyene	*TetR* family transcriptional regulator	571,664 - 572,236	573	*TetR/AcrR* family transcriptional regulator	99.44	WP_042445134.1
**4.7**	Ectoine	Transcriptional regulator, *MerR* family	766,818 - 767,639	822	*MerR* family transcriptional regulator	96.34	WP_084326339.1

Table S7. Antimicrobial activity of concentrated crude extracts from *Rhodococcus* sp. GB-02 against pathogenic microorganisms.

**Pathogenic microorganisms**	**MIC (mg/mL)**	**MBC (mg/mL)**	**Diameter of inhibition zones (mm)**				
			5 mg/mL	10 mg/mL	20 mg/mL	35 mg/mL	50 mg/mL
***Staphylococcus aureus* ATCC 25923**	0,625	5	-	-	8	17	19
***Escherichia coli* ATCC 25922**	5	20	-	-	-	5	12

MIC: Minimum Inhibitory Concentration; MBC: Minimum Bactericidal Concentration; ATCC: American Type Culture Collection; (−): no inhibitory effect

Exploratory molecular docking analyses were performed for peptides 3, 4, and 7 against 21 proteins associated with quorum sensing, virulence regulation, adhesion, and biofilm formation in representative ESKAPEE pathogens (full scores in Supplementary Table S7). These analyses are strictly hypothesis-generating and do not demonstrate antimicrobial activity or functional inhibition. Among the three candidates, peptide 3 showed the highest predicted affinity for the FsrA quorum-sensing regulator of Enterococcus faecium (score −316.19); peptide 4 for the FimH adhesin of Enterobacter cloacae (score −312.81); and peptide 7 for the LasR quorum-sensing regulator of *Pseudomonas aeruginosa* (score −313.71).

Table S8. Top docking scores obtained for AMPs and ESKAPEE bacteria genes.

Species	Protein (PDB ID/Alphafold Structure)	Function	Docking Score (Peptide 3)	Docking Score (Peptide 4)	Docking Score (Peptide 7)
Enterococcus faecium	LacI (A0A133MT58)	Increases biofilm growth in the presence of certain sugar molecules.	-248.51	-224.796	-208.40
	FsrA (C3VD16)	Modulates biofilm genes such as *gelE*, *sprE*, and *bopD*.	**-316.19**	-214.971	-305.75
	FsrB (C3VD17)	A precursor of QS signal molecule, to regulate virulence genes such as *gelE* and *sprE*	-274.56	-249.242	-222.23
Staphylococcus aureus	EsxA (2VRZ)	Establishes hostinfection and controls EsxB production and secretion	-206.17	-181.227	-210.14
	DHFR (2W9G)	Accelerates the formation of *de novo* glycine, purine, and other metabolites, as well as the formation of DNA precursors	-243.87	-205.741	-234.45
	SarX (5HS5)	Binds to the *agr* promoter region and activates the *ica ABCD*	-209.37	-198.496	-232.50

Klebsiella pneumoniae	MrkH (5KED)	Regulates type 3 fimbriae operon by activating *mrkA* promoter.	-277.38	-214.415	-301.79
	MrkA (A0A0H3GSS7)	The major subunit of type 1 fimbriae facilitates binding to abiotic surfaces and rapid biofilm formation	-208.52	-276.372	-203.64
	MrkB (A0A0H3H2G9)	Act as a chaperone together with MrkC and MrkE proteins, helps in the assembly of type 1 fimbriae	-231.90	-205.100	-228.19
Acinetobacter baumannii	AbaR (A0A013UPU7)	receptor for AHLs to trigger a chain reaction	-236.71	-229.361	-223.34
	AdeH (A0A235DA96)	Accelerates the synthesis and transport of AHLs for biofilm formation.	-291.03	-244.282	-266.19
	AbaI (A0A3R9GCQ3)	Acts as an auto-inducersynthase, producing AHL signal molecules, significant for quorum sensing	-196.46	-242.548	-219.48
Pseudomonas aeruginosa	LasI (1RO5)	Helps in LasI/LasR quorum sensing system by promoting the synthesis of PAI	-199.06	-213.362	-202.82
	LasR (2UV0)	Activates the transcription of *lasB* gene, regulates the expression of quorum sensing genes and also binds to PAI autoinducer	-278.35	-249.695	**-313.71**
	LasA (3IT7)	Involved in proteolysis, elastolysis activity.	-291.17	-287.355	-266.56
Enterobacter cloacae	FimA (A0A7H8UJS3)	The major subunit of the type 1 fimbriae, adhesion to host cells and biofilm formation	-182.50	-298.710	-302.75
	CsgD (A0A427KQ30)	Positive transcriptional regulator of the curli fimbriae production and its assembly, facilitating biofilm formation.	-220.11	-218.695	-228.33
	FimH (AF-Q9Z643-F1)	Forms the adhesin tipin type1 fimbriae,which mediates binding to mannose-containing receptors in hostcells and abiotic surfaces.	-233.13	**-312.807**	-224.70
Escherichia coli	DosC (4ZVF)	Likely involved in the synthesis of c-di-GMP, a key signaling molecule for biofilm formation.	-221.01	-195.735	-217.19
	BdcA (5Z2L)	Acts as a "switch" regulating motility and biofilm formation in response to second messengers.	-243.32	-213.816	-249.85
	McbR (4P9F)	A transcriptional repressor of the TetR family. Regulates methionine biosynthesis and biofilm formation, likely by controlling the production of signaling molecules or extracellular matrix components.	-256.48	-192.550	-250.15

Docking Scores are dimensionless values proportional to binding energy (kJ/mol), with more negative scores indicating stronger predicted peptide–protein interactions. LacI, lactose operon repressor; FsrA, response regulator of the Fsr quorum sensing system; FsrB, quorum sensing signal precursor-processing protein; QS, quorum sensing; EsxA/B, ESAT-6–like secretion-associated protein A/B; DHFR, dihydrofolate

reductase; SarX, staphylococcal accessory regulator X; MrkA/B/H, type 3 fimbrial proteins; AbaR, autoinducer receptor; AHLs, *N*-acyl homoserine lactones; AdeH, acyl-homoserine lactone synthase/transport protein; AbaI, autoinducer synthase; LasI, autoinducer synthase; LasR, transcriptional regulator of the Las quorum sensing system; LasA, elastase; PAI, plasminogen activator inhibitor-1 molecule; FimA/H, type 1 fimbrial proteins; CsgD, curli biosynthesis regulator; DosC, dormancy sensor kinase C; c-di-GMP, cyclic di-guanosine monophosphate; BdcA, biofilm dispersal protein A; McbR, transcriptional regulator of microcin B operon.
